# Multiscale predictors of small tree survival across a heterogeneous tropical landscape

**DOI:** 10.1371/journal.pone.0280322

**Published:** 2023-03-15

**Authors:** Eileen H. Helmer, Shannon Kay, Humfredo Marcano-Vega, Jennifer S. Powers, Tana E. Wood, Xiaolin Zhu, David Gwenzi, Thomas S. Ruzycki

**Affiliations:** 1 USDA Forest Service, International Institute of Tropical Forestry, Río Piedras, Puerto Rico, United States of America; 2 USDA Forest Service, Rocky Mountain Research Station Fort Collins, Fort Collins, Colorado, United States of America; 3 USDA Forest Service, Southern Research Station, Asheville, NC, United States of America; 4 Departments of Ecology, Evolution and Behavior and Plant and Microbial Biology, University of Minnesota, St. Paul, Minnesota, United States of America; 5 Department of Land Surveying and Geo-Informatics, The Hong Kong Polytechnic University, Hung Hom, Hong Kong; 6 Department of Environmental Science & Management, Cal Poly Humboldt State University, Arcata, California, United States of America; 7 Center for Environmental Management of Military Lands, Colorado State University, Fort Collins, Colorado, United States of America; Chinese Academy of Forestry, CHINA

## Abstract

Uncertainties about controls on tree mortality make forest responses to land-use and climate change difficult to predict. We tracked biomass of tree functional groups in tropical forest inventories across Puerto Rico and the U.S. Virgin Islands, and with random forests we ranked 86 potential predictors of small tree survival (young or mature stems 2.5–12.6 cm diameter at breast height). Forests span dry to cloud forests, range in age, geology and past land use and experienced severe drought and storms. When excluding species as a predictor, top predictors are tree crown ratio and height, two to three species traits and stand to regional factors reflecting local disturbance and the system state (widespread recovery, drought, hurricanes). Native species, and species with denser wood, taller maximum height, or medium typical height survive longer, but short trees and species survive hurricanes better. Trees survive longer in older stands and with less disturbed canopies, harsher geoclimates (dry, edaphically dry, *e*.*g*., serpentine substrates, and highest-elevation cloud forest), or in intervals removed from hurricanes. Satellite image phenology and bands, even from past decades, are top predictors, being sensitive to vegetation type and disturbance. Covariation between stand-level species traits and geoclimate, disturbance and neighboring species types may explain why most neighbor variables, including introduced *vs*. native species, had low or no importance, despite univariate correlations with survival. As forests recovered from a hurricane in 1998 and earlier deforestation, small trees of introduced species, which on average have lighter wood, died at twice the rate of natives. After hurricanes in 2017, the total biomass of trees ≥12.7 cm dbh of the introduced species *Spathodea campanulata* spiked, suggesting that more frequent hurricanes might perpetuate this light-wooded species commonness. If hurricane recovery favors light-wooded species while drought favors others, climate change influences on forest composition and ecosystem services may depend on the frequency and severity of extreme climate events.

## Introduction

The primary controls that determine the risk of tropical tree mortality are not well understood. This knowledge gap hinders our ability to predict future forest composition, resilience and consequences for carbon storage in response to ongoing changes in climate and land use. Climate change has increased tree mortality from heat, drought and increasingly dry air [1–12], and from wind or rain storms, flooding, landslides and fire [1, 4, 5, 13–18]. Multiple disturbances including forest fragmentation can cause further damage and may synergistically elevate tree mortality [2, 3, 18–22]. Tree mortality drives large changes in tropical forest carbon storage [23–27]. Although large trees are known to play a disproportionate role in tropical carbon cycling [28], small trees can comprise large portions of tree carbon storage in younger or dry forests [29]. Mortality rates are important to evaluating forest roles in Earth systems, climate change vulnerability, restoration success and sustainable management, and to understanding tree species coexistence [30–37]. A challenge, though, is that tree mortality predictions are highly uncertain, in part because the type, severity and frequency of disturbance, and more stable features like gradients in long-term climate, topography and soils, can all affect mortality [1, 8, 11, 34, 38–43].

Adding uncertainty to predicting tree mortality is that in many tropical regions hundreds or more tree species coexist and vary in traits, habitat preferences and species interactions [17, 44, 45]. Tree mortality rates are generally higher for more resource acquisitive species, or for trees within a stand that are smaller or damaged [19, 25, 40, 43–53]. Examples are early successional species, which tend to be shade intolerant and grow fast. However other factors may be more important when considering drought, or for forests frequently disturbed by storms, fire or browsing [10, 12, 41]. Neighboring species with different traits may differently affect mortality, and these effects may differ with climate conditions or species pool [52, 54–56]. For modeling carbon dynamics, functional groups of tree species can simplify this complexity [40, 51]. Unclear, though, is what functional groups or related traits, and what landscape factors, are most important when modeling tree demography across tropical landscapes encompassing widely ranging environments and disturbance histories. Also unclear is what remote sensing products can best support this large-scale modeling [34, 40]. Most knowledge of tropical tree mortality comes from intensely studied plots spanning either disturbance or climate gradients. More studies of tree mortality are needed across complex tropical landscapes to learn what remote sensing products, spatial data, model formulations, and functional groupings of species will improve tree mortality predictions [26, 28, 34], and what factors dominate tree mortality across landscapes over time.

Considering the many questions about the main factors driving tree mortality across diverse and complex tropical regions subject to varied disturbances, we address tropical small tree mortality and regional trends in biomass and basal area of species functional groups of potential neighbor trees, with a series of regional forest inventories from 2001–2019 across Puerto Rico (PR) and the U.S. Virgin Islands (USVI). The region encompasses tropical dry to cloud forests that vary in stand age, lithology and past land use. National forest inventories can reveal what trends and drivers dominate tree demography across a region [57–60]. They capture the net outcome of climate, land use, soil and topography gradients that are layered onto landscapes through a systematic sample of very large areas. Three major drivers of forest change, including land-use change, climate change and species introductions [13] are present in the study area. Current forests underwent widespread pre-1950s deforestation and recent severe drought and hurricanes; introduced species are common.

Our specific objectives are to 1) test potential individual, species trait, neighbor, stand and landscape variables, including from satellite imagery, and compare them as predictors of small tree survival (2.5–12.6 cm diameter at breast height, dbh) and 2) track average change in functional groupings of potential neighbors. To simplify forest modeling and increase sample size, forest models often bin species into functional groups [40, 51, 61, 62]. With this in mind, we limit the survival analysis to predicting individual mortality, rather than individual by species mortality, simultaneously analyzing individual, species-trait and landscape factors as predictors. Though species identity explains much variation tree mortality rates [45], we omitted species as a predictor. This step prevented the combination or interaction of species factors from being selected over the generalization of their individual parts. Which combinations of species traits and landscape factors can be used to accurately predict outcomes, when inventory data encompass both extreme climate events and varied landscapes, remains unclear. Among the landscape factors we test are widely available remote sensing metrics. Additionally, we test two individual tree factors that are not typically available in inventory data: individual tree crown ratio and height.

In all, we tested 86 possible individual, species trait, neighboring tree, site and regional predictors of small tree survival, including disturbance factors. Hypothetical relationships underlie all of the tested predictors (see Methods). We test overall relationships with Bonferroni-corrected univariate tests of survival rates based on the predictors. We then compare predictors of small tree survival with random forest (RF) classification models of survival and use marginal plots [63] to help interpret patterns of survival relative to each variable while accounting for interactions among variables. Limiting the survival analysis to small trees allowed us to test how neighborhood functional groups and conspecific vs. heterospecific neighboring small or large (≥12.7 cm dbh) trees influence small tree survival. To quantify trends in potential neighbors, we estimate average per hectare regional biomass of eight functional groups of small and large trees for each successive inventory. Finally, we discuss connections among predictors of small tree survival, regional events and forest trends.

## Methods

### Study area

Puerto Rico and the USVI are Caribbean Islands with steep environmental gradients. Over land areas of about 8,870 and 350 km^2^, respectively, annual rainfall ranges from 800–4500 mm [64, 65]. Forests in the study area range from xeric to semideciduous tropical dry forest to humid evergreen forest types. In addition to some coastal areas in the north and east, dry forests dominate the side of Puerto Rico’s Cordillera Central that is leeward to trade winds. Humid seasonal evergreen forest zones are the most extensive zones in Puerto Rico. Cloud forests dominate the highest elevations on Puerto Rico’s major cordilleras, where the tallest mountain reaches 1,340 m. Xeric to dry deciduous, semideciduous or coastal forest or woodland dominate lower elevations in the USVI, while seasonal evergreen forests dominate the highest elevations, which reach 470 m on St. Thomas Island. While mangrove forests occur around the islands, the inventory data include only a few mangrove plots, which we excluded here.

Most forest was previously cultivated or grazed, except in the most remote and least arable lands [66]. Forests in the study area underwent large-scale deforestation for agriculture that lasted until the last century for most of the study area. For example, forests covered about 18% of mainland Puerto Rico around 1950 [67]. In the USVI, St. John had some forest by the late 1800s but was the most forested of those islands then [68]. In contrast, recent land-cover maps based on satellite imagery [69] show that tree cover dominates two-thirds of the study area as of the year 2020.

Maps of forest attributes that were developed from the forest inventory data described below, and comparisons of those maps with a review of prior studies, show that the spatial patterns of tree functional traits and diversity reflect this history [70]. They show that nitrogen-fixing trees (all but one individual a legume), leaf traits, forest structure, and numbers of introduced, native and endemic species are related to forest age and past land use [66, 70–73], which in turn are related to topography, rainfall and geological substrate [66, 70, 72] (S1 File). The driest forests, forests on fast-draining serpentine substrates and karst hilltops, and cloud forests, have thicker and smaller leaves and more native and endemic species compared to other volcanic substrates and alluvial soils (S1 File) [70, 74–76]. In places with more fertile soils or that are most accessible, forests are younger, more deciduous, and have larger relative basal areas of nitrogen-fixing trees and introduced species [66, 73]. Deciduousness and N-fixing trees are also more common in drier zones [70].

### Forest inventories

Successive inventories were conducted jointly by the United States Department of Agriculture Forest Service (USDA FS) Southern Research Station, Forest Inventory and Analysis Program and the USDA FS International Institute of Tropical Forestry on a five-year cycle across Puerto Rico and the USVI. Each 5-yr cycle surveys a systematically distributed one-third of the Puerto Rico plots during each of the first three years of a cycle. Plots on the outlying islands of Puerto Rico, including Vieques, Culebra and Mona Island, and those on the USVI, are surveyed once every five years during years 3–4 of each cycle, though surveys on Mona and the USVI were not surveyed in the 2016–2019 cycle, and Mona was first surveyed in the year 2008. In all, the inventories included 9,616 observations of small tree survival or mortality from one inventory cycle to the next of 191 unique species. We spaced plots every 24 km^2^ on mainland Puerto Rico and every 2–4 km^2^ on other islands. Large trees ≥12.7 cm dbh are surveyed on four circular 0.016 ha subplots with centers spaced 36.6 m apart. Small trees 2.5–12.6 cm dbh are surveyed on 2.07-m radius circular microplots within each subplot. We only analyzed trees on fully forested microplots or subplots, defined as having >10% tree cover or, in earlier inventories, 10% of the density expected for a mature stand [77]. The inventories were conducted in the years 2001–2004 (t1, three to six years after Hurricane Georges in 1998), 2006–2009 (t2), 2010–2014 (t3), 2016–2017 (t4a, during and after the severe 2015–2016 drought), and 2017–2019 (t4b, after Hurricanes Irma and Maria). Changes to the roster of core inventory crew members during that time included only one of three core members. There were 724 fully forested subplots with observations of small tree survival or mortality from t1-t2, 869 from t2-t3, 342 from t3-t4a and 247 from t3-t4b. Resprouting downed stems are not counted as survivors. Resprouts are counted as recruits. Because of the major drought and hurricanes, we analyzed the intervals separately, and we analyzed an all-periods model.

### Temporal trends in functional groups

We estimated average per hectare aboveground live biomass for each time period of eight functional groups of tree species. The aim was to characterize overall trends through time in small tree neighborhoods and selected forest functional groups. The summaries rely on design-based estimation methods [78] that account for differences in plot spatial density, though we limited the estimates to mainland Puerto Rico. Seven functional groups were based on combinations of three traits after dropping one rare combination. These traits included species origin (Origin, I = Introduced, N = Native), Leaf Habit (E = Evergreen, D = Deciduous or Facultatively Deciduous) and Nitrogen fixing status (N = N-fixer, 0N = non-N-fixer). An example group is introduced evergreen non-N-fixing species (IE0N, excluding *Spathodea campanulata*, Boraginaceae). Introduced evergreen N-fixers (IEN) were rare and dropped. The eighth group was *S*. *campanula*, assigned its own functional group because it represents an introduced fast-growing pioneer taking advantage of degraded forest ecosystems [79] and competing with native vegetation. This niche has resulted in it either coexisting with or being a threat to native species [80–85]. Consequently, it represents a conspicuous species to monitor within various tropical insular landscapes, especially because its seedlings may have some shade tolerance [80].

### Small tree survival predictors

We tested 86 possible individual, species trait, neighboring tree, site, and regional predictors of small tree survival, including disturbance factors and remote sensing products (Tables 1–3). As described in the Introduction, we excluded species as a predictor to focus the analysis on functional traits, neighbors, landscape factors and regional extreme events.

**Table 1:** Individual, species trait or field-collected predictors and potential influence (PI) on survival.

Variable Name	Scale	Units	Description	PI +/-
**CCLCD_Beg**	Individual	Ordinal	Canopy layer, as indicated by crown class code at interval beginning (1 = Open grown, 2 = Dominant, 3 = Codominant, 4 = Intermediate, 5 = Overtopped)	+ if code is smaller
**CR_Beg**	Individual	Percent	Compacted crown ratio. The percent of the tree bole supporting live, healthy foliage	+/-
**DIA_Beg**	Individual	cm	Diameter in the last inventory	+
**HT_Beg**	Individual	m	Height in the last inventory	+
**Deciduous**	Species	Nominal	Leaf habit / deciduousness: E = Evergreen, D = Deciduous, DE = Deciduous or Evergreen	+/-
**Leaf_Thickness**	Species	Nominal	Leaf thickness class: Chartaceous, Chartaceous to Subcoriaceous, Subcoriaceous, Coriaceous, Succulent	+ if thicker
**Myco_group**	Species	Nominal	Mycorrhizal association group–arbuscular mycorrhizal (AM), ectomycorrhizal (EM), nonmycorrhizal (NM), No data, AM+EM, or AM+NM	+/- (- if AM)
**N-fixer**	Species	Binary	Nitrogen-fixing status: yes = nitrogen fixer, no = non-nitrogen fixer	+ if yes
**Origin**	Species	Binary	Origin: I = Introduced, N = Native	+ if Native
**Species_Ht**	Species	m	Tree species typical height from field guides and flora	+/-
**Species_Ht_Max**	Species	m	Maximum of Species_Ht or tallest individual in the PRVI inventory	+/-
**Wood_Density**	Species	unitless	Wood density from new field data, averages of new with online data, or online data averages (g dry mass/cm3 dry: see text)	+
**Canopy Cover**	Subplot	Percent	Tree canopy cover from field ocular method for t3, t4a and t4b and circa the year 2000 from remote sensing for t1 and t2	+
**Disturbance**	Subplot	Nominal	Disturbance type in the last five years	+/-
**Stand size**	Subplot	Ordinal	Large, medium, small	+/-
**t**	Inventory	Nominal	Inventory period end (t2: 2006–2010, t3: 2011–2014, t4a: 2016–2017 post-drought/pre-hurricanes Maria/Irma, t4b: 2017–2019 post-hurricanes Maria/Irma)	+/-

Individual and subplot-level variables were from the forest inventory data (canopy cover in t2-t3 is from remote sensing). Species-level traits (https://doi.org/10.2737/RDS-2023-0004) compiled or measured for this or a prior study [70]. PI +/- = Overall potential influence on survival is positive (+), negative (-), or dependent on time and place (+/-).

**Table 2:** Functional groups of potential neighbor predictor variables and influence on survival.

Functional Group^a^^-^^c^ (Figs 1 and 11)	Neighbor variable Name (Fig 2)	Neighbor conspecificity	Size	Species Origin^b^	N-fixing	Leaf Habit^c^	Potential Influence (PI) Type^d^	PI +/-
**ID0N**	conspLg_Tr1ID0Nba	conspecific	large	I	No	D	N, S	-
**IDN**	conspLg_Tr1IDNba	conspecific	large	I	Yes	D	N, S, Nutr	-
**IE0N**	conspLg_Tr1IE0Nba	conspecific	large	I	No	D	N, S	-
**ND0N**	conspLg_Tr1ND0Nba	conspecific	large	N	No	E	N, S	-
**NDN**	conspLg_Tr1NDNba	conspecific	large	N	Yes	D	N, S, Nutr	-
**NE0N**	conspLg_Tr1NE0Nba	conspecific	large	N	No	D	N, S	-
**NEN**	conspLg_Tr1NENba	conspecific	large	N	Yes	E	N, S, Nutr	-
**SPCA**	conspLg_Tr1SPCAba	conspecific	small	I	No	E	N, S	-
**ID0N**	consp_SmTr1ID0Nba	conspecific	small	I	No	D	N, S	-
**IDN**	consp_SmTr1IDNba	conspecific	small	I	Yes	D	N, S, Nutr	-
**IE0N**	consp_SmTr1IE0Nba	conspecific	small	I	No	D	N, S	-
**ND0N**	consp_SmTr1ND0Nba	conspecific	small	N	No	E	N, S	-
**NDN**	consp_SmTr1NDNba	conspecific	small	N	Yes	D	N, S, Nutr	-
**NE0N**	consp_SmTr1NE0Nba	conspecific	small	N	No	D	N, S	-
**NEN**	consp_SmTr1NENba	conspecific	small	N	Yes	E	N, S, Nutr	-
**SPCA**	consp_SmTr1SPCAba	conspecific	large	I	No	E	N, S	-
**ID0N**	hetspLg_Tr1ID0Nba	heterospecific	large	I	No	D	N, S	+/-
**IDN**	hetspLg_Tr1IDNba	heterospecific	large	I	Yes	D	N, S, Nutr	+/-
**IE0N**	hetspLg_Tr1IE0Nba	heterospecific	large	I	No	D	N, S	+/-
**ND0N**	hetspLg_Tr1IENba	heterospecific	large	I	Yes	E	N, S, Nutr	+/-
**NDN**	hetspLg_Tr1ND0Nba	heterospecific	large	N	No	E	N, S	+/-
**NE0N**	hetspLg_Tr1NDNba	heterospecific	large	N	Yes	D	N, S, Nutr	+/-
**NEN**	hetspLg_Tr1NE0Nba	heterospecific	large	N	No	D	N, S	+/-
**SPCA**	hetspLg_Tr1NENba	heterospecific	large	N	Yes	E	N, S, Nutr	+/-
**ID0N**	hetspLg_Tr1SPCAba	heterospecific	large	I	No	E	N, S	+/-
**ID0N**	hetsp_SmTr1ID0Nba	heterospecific	small	I	No	D	N, S	+/-
**IDN**	hetsp_SmTr1IDNba	heterospecific	small	I	Yes	D	N, S, Nutr	+/-
**IE0N**	hetsp_SmTr1IE0Nba	heterospecific	small	I	No	D	N, S	+/-
**ND0N**	hetsp_SmTr1ND0Nba	heterospecific	small	N	No	E	N, S	+/-
**NDN**	hetsp_SmTr1NDNba	heterospecific	small	N	Yes	D	N, S, Nutr	+/-
**NE0N**	hetsp_SmTr1NE0Nba	heterospecific	small	N	No	D	N, S	+/-
**NEN**	hetsp_SmTr1NENba	heterospecific	small	N	Yes	E	N, S, Nutr	+/-
**SPCA**	hetsp_SmTr1SPCAba	heterospecific	small	I	No	E	N, S	+/-

^a^Introduced evergreen nitrogen-fixing species were rare.

^b^I = Introduced species, N = Native species

^c^E = Evergreen species, D = Deciduous, Nearly deciduous or facultatively deciduous species.

^d^N = Neighbor contagion, immunity, competition, etc., S = Shade environment, Nutr = Nutrient availability

^e^+ = Hypothetical positive influence on individual survival, - = Hypothetical negative influence, +/- = Hypothetical negative/positive influence on species that are shade-intolerant/shade-tolerant.

**Table 3:** Potential spatial predictor variables may relate to shade, moisture, nutrients, or disturbance.

Variable	Units	Description^a^	Source	PI+/-^b^
**age**	Years	Stand age since disturbance	This study	+
**age_2001**	Years	Stand age in 2001 since disturbance	This study	+
**amplitude10_14**	Unitless	Landsat phenology maximum minus minimum Enhanced Vegetation Index (EVI) for 2010–2014	This study	+/-
**cloudfor**	Binary	1 = cloud forest zone,0 = not cloud forest zone	[86, 87]	+/-
**coffee77**	Binary	Whether a subplot was coffee in 1977 or not	[67]	-
**curvature**	Radians/distance	Topographic curvature(<0 = concave, 0 = flat, 0 = convex)	[88]	+/-
**depth2restrictive**	cm	Depth to restrictive layer	[89]	+
**depth2water_table**	cm	Depth to water table	[89]	+
**eastness**	Unitless	Topographic eastness facing west/east (-1/+1)	[88]	+/-
**elevation**	m	Topographic elevation—lower values are drier	[88]	+/-
**geoclimate**	Nominal	Geoclimatic zone (potential evapotranspiration to precipitation ratio <1 = humid or >1 = dry), three-class geology below, and cloud forest from [86]	[64, 70, 86, 87, 90, 91]	+/-
**geology_3class**	Nominal	Surficial geology generalized to 3 classes: karst, serpentine, or other (extrusive volcanic + sedimentary + alluvial)	[70, 90, 91]	+/-
**integrdryseas10_14**	EVI*day	Landsat phenology dry season integral, 2010–2014	This study	+/-
**life zone**	Nominal	Holdridge life zone	[92]	+/-
**island_group**	Nominal	PR = mainland Puerto Rico; MO = Mona; VC = Vieques and Culebra; VI = US Virgin Islands	-	-
**maximum.EVI10_14**	Unitless	Landsat phenology Maximum EVI for 2010–2014	This study	+/-
**minimum.EVI10_14**	Unitless	Landsat phenology Minimum EVI for 2010–2014	This study	+/-
**ndvi**	DN	Landsat ETM+/OLI ndvi	[93]	+/-
**ndvi_1980**	DN	Landsat MSS ndvi circa 1980	[70]	+
**ndvi_1985**	DN	Landsat MSS ndvi circa 1985	[70]	+
**ndvi_1990**	DN	Landsat TM ndvi circa 1990	[70]	+
**northness**	Unitless	Topographic Northness, facing north/south (-1/+1)	[88]	+
**pan_1995**	DN	SPOT Panchromatic composite circa 1995	[70]	-
**PET2Driest**	Unitless	Potential evapotranspiration to precipitation ratio of the driest four months from long-term climate	[64]	+/-
**PET2Pannual**	Unitless	Annual potential evapotranspiration to precipitation ratio from long-term climate	[64]	+/-
**r45**	DN	Landsat ETM+/OLI, near infrared:shortwave infrared ratio	[93]	+
**r45_1990**	DN	Landsat TM, rescaled near infrared:shortwave infrared ratio circa 1990	[70]	+
**radiation**	Unitless	Topographic radiation index	[88]	+
**red**	DN	Red band from Landsat ETM+/OLI	[93]	-
**red_1980**	DN	Landsat MSS red band circa 1980	[70]	-
**red_1985**	DN	Landsat MSS red band circa 1985	[70]	-
**red_1990**	DN	Landsat TM red band circa 1990	[70]	-
**serpentine**	Binary	Surficial geology is serpentine or not	[35, 49, 50]	+
**slope**	Degrees	Topographic slope in degrees	[88]	+/-
**swir1**	DN	Landsat ETM+/OLI rescaled ShortWave InfraRed	[93]	-
**swir1_1990**	DN	Landsat TM ShortWave InfraRed circa 1990	[70]	-

^a^MSS = Multispectral Scanner, TM = Thematic Mapper, ETM+ = Enhanced TM, OLI = Operational Land Imager, EVI = Enhanced Vegetation Index, ndvi = normalized difference vegetation index.

^b^+/- = Overall potential influence on survival is positive (+), negative (-), or dependent on time and place (+/-).

#### Individual tree size and shade environment

Individual tree measurements representing tree size and light environment included interval beginning dbh (DIA_Beg), height (HT_Beg), the percent of the tree stem supporting live foliage (compacted crown ratio, CR_Beg) and canopy position (CCLCD_Beg) (Table 1).

#### Species traits

A database of species functional groups and species stature, *i*.*e*., typical mature height (Species_Ht) was produced from field guides, flora and other sources and includes leaf thickness, leaf deciduousness, nitrogen-fixing (N-fixing) status and origin (introduced or not) [70]. We added wood density and mycorrhizal association (https://doi.org/10.2737/RDS-2023-0004). Wood density came from this study, a global dataset [94], or averages of those sources. If not available from the former, we used two other datasets [95, 96]. Mycorrhizal associations are based on species, genus, or family and came from several sources [97–104]. We also tested species maximum height (Ht_species_max), the maximum of the tallest example of a species in the inventory data or tallest individual in the PRVI inventory.

#### Stand and neighborhood

**Fig 1:**
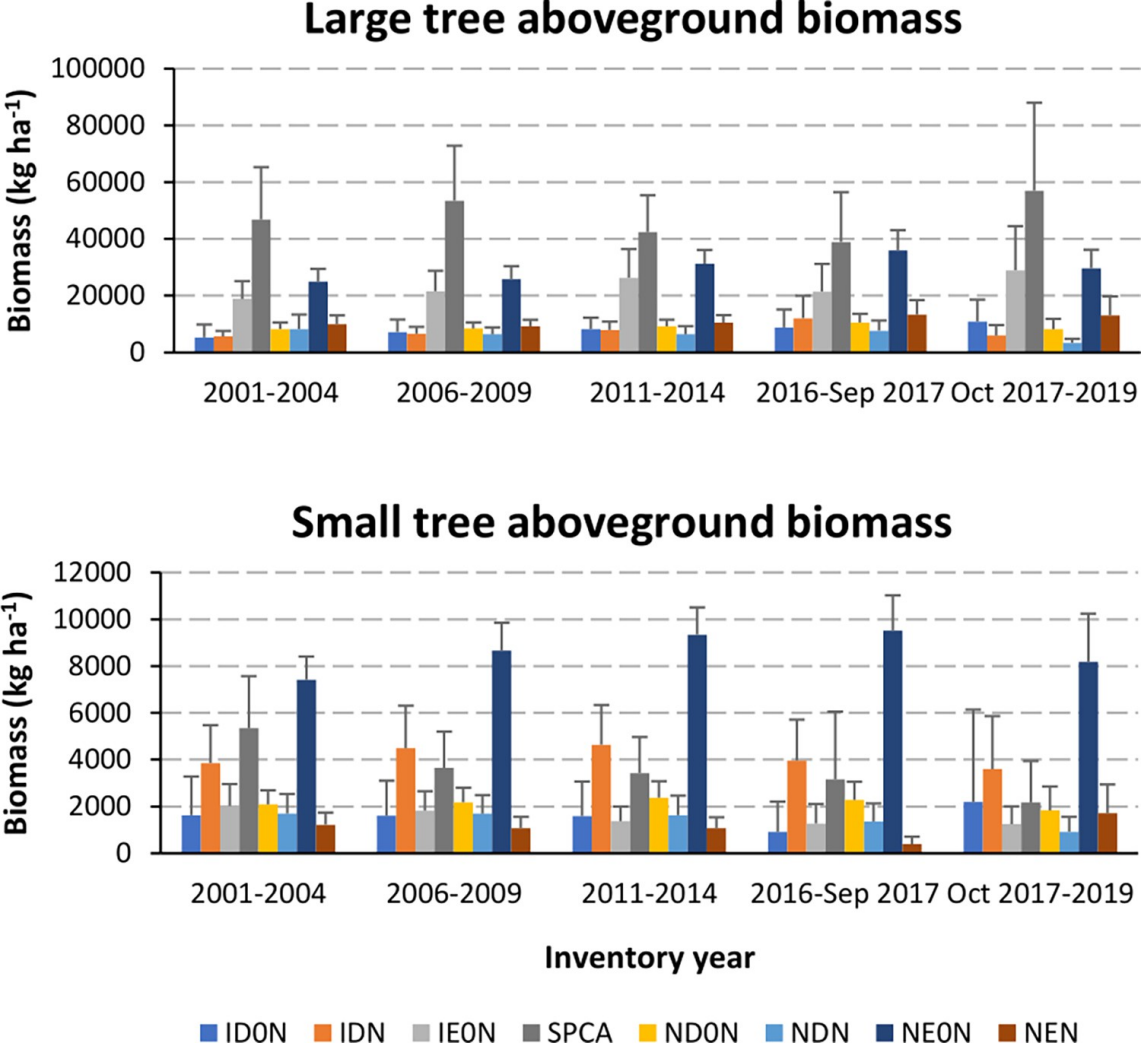
Trends in aboveground live biomass of tree functional groups. Aboveground live biomass (kg dry weight per ha of forest) of eight tree functional groups of large (≥12.7 cm dbh) and small (2.5–12.6 cm dbh) trees. Functional groups are by Origin (I = Introduced, N = Native), Leaf Habit (E = Evergreen, D = Deciduous or Facultatively Deciduous) and Nitrogen fixing status (N = N-fixer, 0N = non-N-fixer). Combined abbreviations are: ID0N = Introduced Deciduous non-N-fixers, IDN = Introduced Deciduous N-fixers, IE0N = Introduced Evergreen non-N-Fixers, SPCA = *Spathodea campanulata* (assigned its own group; it is an Introduced Deciduous non-N-fixer), ND0N = Native Deciduous non-N-fixers, NDN = Native Deciduous N-fixers, NE0N = Native Evergreen non-N-Fixers, NEN = Native Evergreen N-fixers. Introduced Evergreen N-fixers were rare. Surveys began in t1 (2001–2004); t2 ended in 2006–2009; t3 ended in 2011–2014; t4a ended in 2016 to September 2017, before Hurricane Maria; t4b ended in October 2017 to 2019.

We define the neighborhood functional groups by species traits that prior studies show can affect seedling, sapling, or tree neighbors. The traits include deciduousness [18, 28, 35], N-fixing status [29, 36–38], nativity [39] and whether the species is conspecific or heterospecific [18, 40, 41]. Some of these traits can be empirically modeled at the individual or stand level with available space borne remote sensing and, typically, other spatial data [70, 105–107]. Stand-level variables from field data included stand size class, field-noted disturbance type in the last five years, canopy cover in t4a and t4b, and variables describing neighboring trees (Table 2). These neighborhood variables included basal areas of 1) surrounding conspecific stems and 2) surrounding heterospecific stems, each divided into small trees *vs*. large trees, of the eight functional groups described above, in Table 2 and in the legend for Fig 1. The field-determined disturbance type in the last five years included multiple disturbances, like wind or rain plus fire, wind or rain plus human disturbance and wind or rain plus a landslide. Other indicators of stand-level disturbance severity are described below with Landscape predictors.

#### Landscape

Landscape variables represented climate, surficial geology, soil depth, water table depth, topography, disturbance history and shade environment (Table 3). Long-term climate measures included 30-yr potential evapotranspiration to precipitation ratio for the year (PET2Pannual) and the four driest months (PET2Pdry). Ongoing work is analyzing how climate indicators of drought and storm severity relate to mortality. Disturbance history, shade environment and seasonality variables included remote sensing products that now or likely soon will be available across the tropics that might improve spatially explicit modeling of forest dynamics. They included tree canopy cover for periods when not available from field data. They also included stand age since last disturbance and the red, shortwave infrared (swir), near infrared to swir ratio (r45) and normalized difference vegetation index (ndvi = (nir—red)/(nir + red)) from a time series of mostly Landsat satellite image composites from 1980, 1985, 1990, 1995 and contemporaneous to each period. Metrics of average greenness seasonality (phenology), were produced from Landsat images dated in the years 2010–2014 with Landsat image cloud-masking and reconstruction methods previously described [108]. We included simple phenology metrics to minimize error from having few clear observations of a pixel. The four metrics were minimum and maximum Enhanced Vegetation Index (EVI), their difference (amplitude), and a measure integrating dry season length and intensity (integral of the dry season). We assumed greenness in 2010–2014 was least affected by the climate disturbances in 1998 and 2015–2017. The recent and past image bands and metrics chosen can help distinguish forest type and gauge recent or past disturbance intensity or stand structure, based on previous studies of Caribbean forest [109–111].

We estimated stand age in early 2001 (age_2001) for mainland Puerto Rico plots with land-cover maps from the years 1951–52, 1977–78, 1991–92 and 2000–03 [67] and photointerpretation of forest vs. non-forest in air photos from the years 1936–37. We orthorectified air photo pairs from 1936–37 with an average of 27 manually verified reference points using ERDAS Imagine AutoSync [112]. Average root mean square error for the orthorectifications was 6.5 m. For other islands we modeled stand age in 2001 with a discriminant function model of age class in mainland Puerto Rico as predicted by year 2000–03 satellite imagery and forest cover (in 2000–03) in 90-, 270-, and 1,050-m windows around each training point, as surrounding forest cover is a top predictor of forest age [68] (a DOI link to https://www.fs.usda.gov/rds/archive/ will be placed here). Stand age (age) came from age in the year 2001 and whether a plot was forested in 2001 to 2019. We also included a variable from [67] indicating whether a stand was shade coffee in 1977–78 (coffee_77).

### Univariate statistics

We summarized the univariate impact of each predictor variable on small tree survival by calculating the effect size and testing for differences in the predictor variable between survivors and mortality. For numeric variables, we conducted a non-parametric Mann-Whitney U test, testing the hypothesis that for two randomly selected observations from the survivors and mortality, the probability that the numeric variable is greater in survivors than in mortality is equal to the probability that the numeric variable is greater in mortality than in survivors [113]. The effect size for numeric variables was calculated as the Cohen’s d statistic [114], the standardized difference between two means, $d=|\frac{\left({\bar{x}}_{1}-{\bar{x}}_{2}\right)}{s}|$ where ${\bar{x}}_{1}$ is the sample mean of the numeric variable for non-survivors, ${\bar{x}}_{2}$ is the sample mean of the numeric variable for survivors, and *s* is the pooled standard deviation, $s=\sqrt{\frac{(n_{1}-1)s_{1}^{2}+(n_{2}-1)s_{2}^{2}}{n_{1}+n_{2}-2}}$. For categorical variables, we tested the null hypothesis that the survival proportions of each level of the categorical variable were equal [115]. The effect size for categorical variables was represented by the Cohen’s h statistic using the minimum and maximum survival proportions (p_1_, p_2_) among the levels of the variable, *h* = |2*arcsin*√(p_1_) -2*arcsin*√(p_2_) |. Effect sizes are considered small if the statistic is less than 0.2, medium if between 0.2 and 0.8, and large if greater than 0.8 [50]. To account for multiple comparisons, we adjusted the *p-*values from the statistical tests within each time-period using the Bonferroni correction.

We used a one-way ANOVA to test for differences in mean wood density, mean species height, and mean species maximum height between geoclimate and neighbor groups. Pairwise comparisons were performed using Tukey’s Honest Significant Difference tests to obtain significantly different groupings within each response.

### Variable reduction and model fitting

We first determined which variables were stronger predictors of small tree survival with a random forest variable selection wrapper, VSURF [116], from the statistical software package R [117]. VSURF is a machine-learning algorithm based on fitting a series of random forests, which are non-parametric, have few assumptions, can accommodate correlated variable inputs and have high predictive accuracy [118]. It returns two subsets of variables: the interpretation set and a smaller, prediction set. The algorithm begins with ‘thresholding’ variables, sorting them by mean variable importance and omitting the weakest ones (*i*.*e*., the ones with variable importance measures below a threshold). The remaining variables are used in a set of nested models, where the OOB (out-of-bag) error rate is computed for each model by comparing the model with only the most important variables with a model with all of the variables included from the thresholding step. The variables included in the smallest model with an OOB error less than the minimal OOB error augmented by its standard deviation are chosen as the ‘interpretation’ set of variables. Then a stepwise approach is taken from this set of ordered variables, where a variable is added if the error decrease is larger than the threshold. The set of variables from the last model are chosen as the ‘prediction’ set of variables. Thus, after a screening of variables (‘thresholding’ step), the algorithm does a stepwise backward elimination (‘interpretation’ step) and then a forward selection procedure (‘prediction’ step).

We retained the variables from the interpretation step of VSURF for each period for potential selection in fitting a final random forest model, fit using the randomForestSRC package [119]. Permuted variable importance scores were computed for each variable that was included in the final models by randomly permuting values of the variable and measuring the resulting difference in classification accuracy. Minimal depth importance scores were calculated by measuring how far down each tree a variable was used for splitting, where variables that tend to be split first (higher in the tree) are believed to be more important. We ranked each variable based on the two variable importance measures and plotted the one-to-one relationship.

To evaluate the predictive accuracy for each final random forest model, we split the data 60/40 into two parts for training and testing and present the confusion matrix for the out-of-sample (testing) data. We also report a set of goodness-of-fit statistics calculated from the testing data using the caret package [120], including accuracy and it’s 95% confidence interval, Cohen’s Kappa [121], and the no information rate (NIR). Accuracy is the proportion of observations that were correctly classified as survived/died, and the NIR is the largest class percentage in the data and represents the possibility of correct classification by chance alone. Kappa is a common classification measure for quantifying agreement between predictions and observations much like accuracy, but also takes into account class imbalance with values near zero indicating agreement equivalent to chance and values near one indicating almost perfect agreement [121–123]. The *p-*value for a one-sided test of accuracy being greater than the NIR is also reported (*i*.*e*., testing whether the model performs any better than randomly guessing).

### Marginal plots

To explore the directions and forms of relationships between survival and predictor variables while considering predictor interactions, we used plots of marginal variable dependence [63]. They plot random forest-predicted survival probability for each observation against one predictor variable, showing uncertainty related to all observed combinations of other variables. They provide a way to show marginal dependence for variables in non-parametric models. We used a Loess smoother for all of the continuous predictors to graph the average probability of survival for each value of the predictor.

## Results

### Regional trends in functional groups

Within 2 yr after Hurricanes Irma and Maria in 2017 (t4b), average regional per hectare biomass of large trees of the introduced *S*. *campanulata* (SPCA) increased by almost 50% on mainland Puerto Rico (Fig 1). Introduced non-N-fixing evergreen (IE0N) species also increased. Before then, from 2006–09 to 2016–17, large tree biomass of these groups declined or changed little. Large *S*. *campanulata* also increased from 2001–04 to 2006–09, the interval after Hurricane Georges in 1998. Small trees of native N-fixing evergreen species (NEN) declined after the drought in 2015–16 (t4a) but increased after the hurricanes. Biomass of both large and small native evergreen non-N-fixing species (NE0N) increased from 2001–04 to 2016–17, before the recent hurricanes, but dropped afterwards. Regional shifts in per hectare basal areas paralleled those for biomass.

### Univariate differences in survival rates

**Fig 2:**
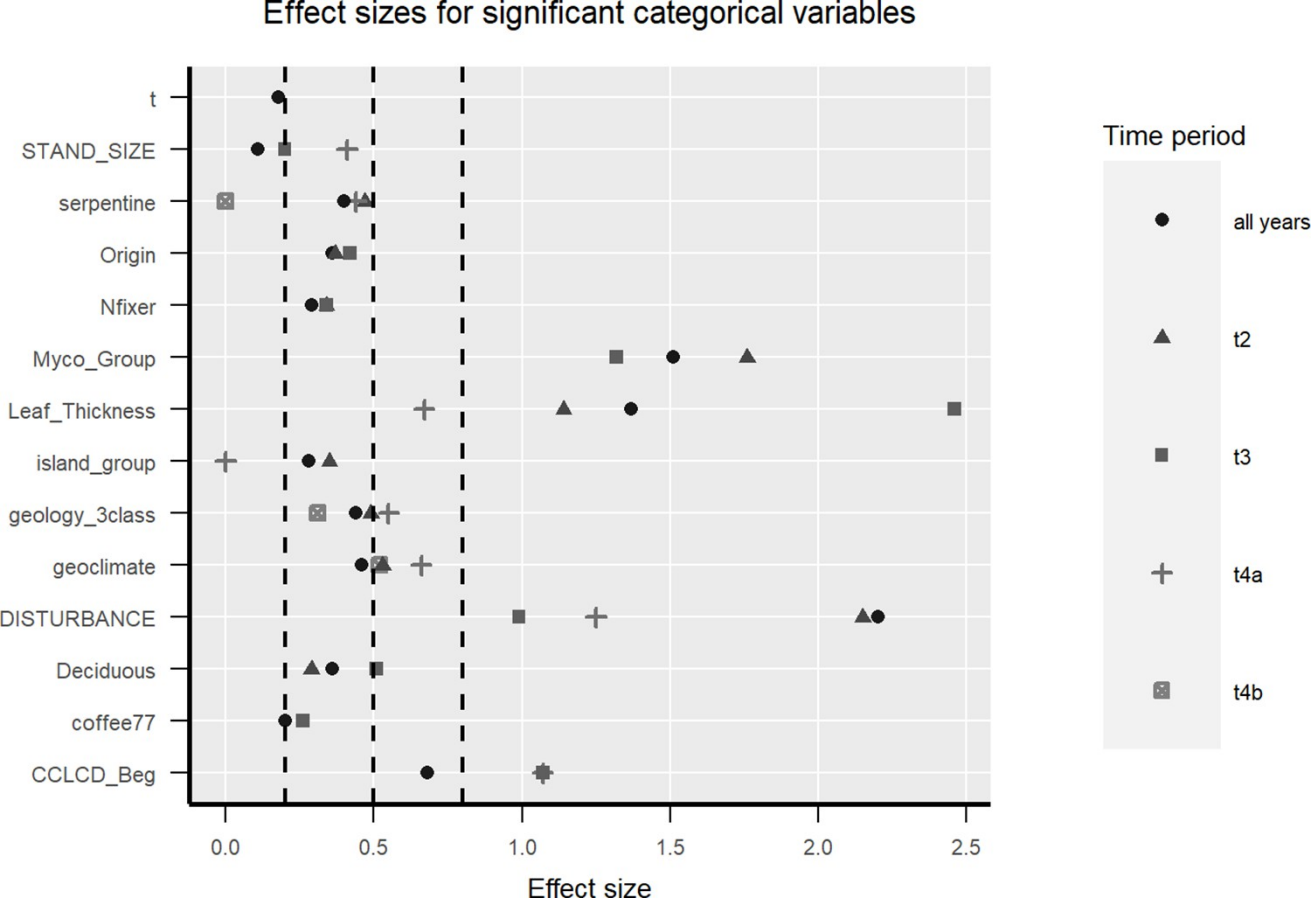
Univariate effect sizes for significant discrete variables. Class means for the data from all periods combined are shown in Fig 1 in S2 File. Dashed lines indicate categories of effect sizes. Effect sizes are considered small if <0.2, medium if between 0.2 and 0.8, and large if >0.8.

Overall survival rates were significantly lower in the intervals associated with hurricanes: one of these, t2, began in t1 in the years 2001–2004 after Hurricane Georges in 1998, and the other spanned Hurricanes Maria and Irma in 2017 (t4b end) (variable t in Fig 2 and Fig 1 in S2 File). As for spatial variables (Fig 2 in S2 File), across all periods survival rates were highest for older forests, forests on serpentine substrate, drier forests, and forests at higher elevation, on steeper slopes, or facing north. Stands that were coffee cultivation in 1977 had overall lower survival. The most common species in the data, with 1120 observations, is *Leucaena leucocephala*, followed by *Guarea guidonia* with 483 observations. Over all periods, most small trees survived (76%).

**Fig 3:**
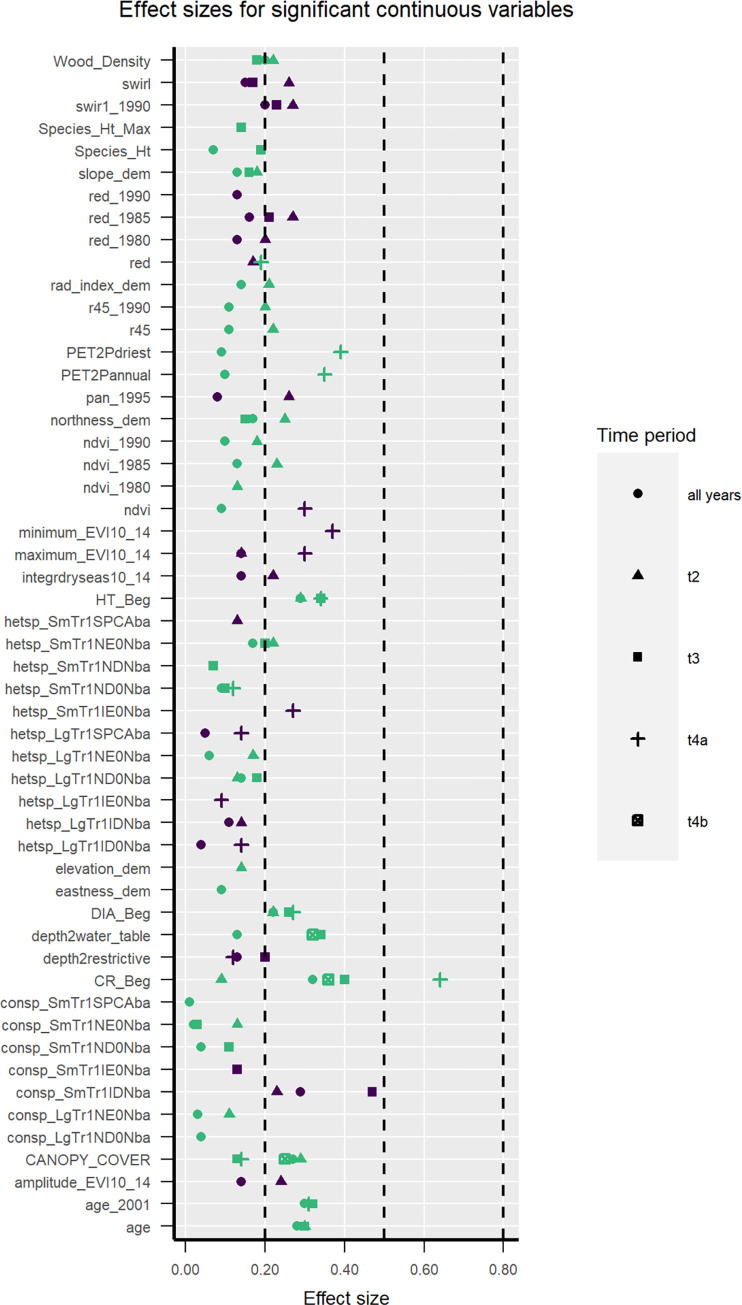
Univariate effect sizes for significant continuous variables. Green are positive relationships; purple are negative relationships. Class means for spatial variables for the data from all periods combined are shown in Fig 2 in S2 File. Dashed lines indicate categories of effect sizes. Effect sizes are considered small if <0.2, medium if between 0.2 and 0.8, and large if >0.8.

Of individual and species trait variables, the odds of a non-N-fixing individual surviving (3.8) were almost twice the odds of surviving for a N-fixing individual (2.0). Similarly, the odds of a native individual surviving (4.2) are over twice as high as the odds of an introduced individual surviving (1.9). Significantly lower survival rates also occurred for species with arbuscular mycorrhizal associations, thinner leaves, deciduous leaves, less dense wood, or shorter maximum heights (Fig 2, Fig 1 in S2 File). Facultatively deciduous species are significantly more likely to survive than evergreen ones. Categorical variables tend to have larger effect sizes than continuous ones. Effect sizes for leaf thickness, mycorrhizal association, disturbance type and canopy position are largest, followed by geoclimate and geology (Figs 2 and 3). The variable with the largest effect size and a significant univariate test was leaf thickness in the 2011–2014 period. For all years combined, canopy position, mycorrhizal group, leaf thickness and disturbance type significantly differed between survivors and mortality and had an effect size larger than 0.5. For the 2006–2010 period, mycorrhizal group, leaf thickness, disturbance type and geoclimate met those criteria. In the 2011–2014 period canopy position, deciduousness, mycorrhizal group, leaf thickness and disturbance type had significant differences with an effect size greater than 0.5. For the drought period, the variables mycorrhizal group, crown ratio, leaf thickness, disturbance type, geoclimate, and geology met the criteria. For the post-Maria/Irma period, geoclimate did.

Many neighbor variables differed significantly between survivors and mortality (Fig 3). Those which also have effect sizes >0.2 included small introduced deciduous N-fixing (IDN) trees, which correlated negatively with conspecific response tree survival; small, introduced evergreen non-N-fixing (IE0N) trees, which correlated negatively with heterospecific response trees; and small native evergreen non-N-fixing (NE0N) trees, which correlated positively with heterospecific response trees. Due to many zeroes and no variation within certain periods, we did not test neighbor variables for introduced N-fixing evergreens.

Landscape variables with effect sizes >0.2 and significant differences between survivors and mortality included stand age, canopy cover, water table depth and some satellite image bands or metrics (Fig 3, Figs 1, 2 in S2 File). The swir band from 15–25 yr previous most often met those criteria. During the interval spanning the drought (*i*.*e*., t4a end), satellite image greenness variables indicating evergreen forest zones have large negative effect sizes, while large effect sizes with positive relationships with survival occur for crown ratio and drier long-term climate (larger PET2P).

### Variable selection

Individual, species, stand and spatial variables had the most selection agreement across periods with the variable reduction step VSURF, and neighbor variables were least likely to be selected. Two individual and one species variable were selected across all periods: individual tree height (Ht_Beg), individual crown ratio (CR_Beg) and maximum species height (Species_Ht_Max). Wood density (Wood_Density) was selected in all but t4a, ending 2016–2017 and spanning the drought, while canopy layer (crown class code, CCLCD_Beg), tree canopy cover (CANOPY_COVER), individual diameter (DIA_Beg), species height (Species_Ht), and whether a species was native or introduced (Origin) were selected in 3 out of 5 periods. Of the spatial variables, elevation was the only variable selected in all 5 periods, but many spatial variables were selected in at least 2 periods. Only the shortwave infrared band from 1990 (swir1_1990), minimum and maximum EVI (min/max EVI10_14), soil depth to water table (depth2water_table), soil depth to restrictive layer (depth2restrictive), and stand age in 2001 (age_2001) were not selected in any periods.

### Final random forest models

The RF models for all years and for each interval have accuracies of 0.77–0.83 and are significantly more accurate than the NIR values of 0.71–0.79 for the all-periods, t1 and t2 models (p-values all <0.001), but not for the last two time periods (p-values of 0.19 for pre-Maria and 0.08 for post-Maria) (Table 4). Crown ratio, individual tree height, and a disturbance factor are top variables in random forest models.

**Table 4:** Accuracy measures from out-of-sample test data for final random forest models.

	All years	2006–2010	2011–2014	2016–2017	2017–2019
**Accuracy**	0.82	0.83	0.83	0.81	0.77
**Balanced accuracy**	0.67	0.74	0.65	0.65	0.65
**Sensitivity**	0.94	0.95	0.95	0.92	0.90
**Specificity**	0.40	0.52	0.36	0.38	0.40
**Kappa**	0.41	0.53	0.37	0.34	0.34
**NIR**	0.77	0.71	0.79	0.79	0.74
**p-value Accuracy>NIR**	0.00	0.00	0.00	0.19	0.08

In the all-periods model, variable importance rankings closely agreed. The most import variables according to both permuted importance and minimal depth were crown ratio, species origin and disturbance type in the last five years (DISTURBANCE), followed by individual height, species height and canopy cover (ordered from individual to species to landscape predictors) (Fig 3 in S2 File). Both measures also ranked the Landsat red band from 1985 (red_1985) relatively highly. Disturbance types included wind or rain, landslides, fire, grazing, flooding, unknown, human disturbance not otherwise defined (and excluding harvest), or multiple disturbances (e.g., wind_rain_fire) in the last five years.

**Fig 4:**
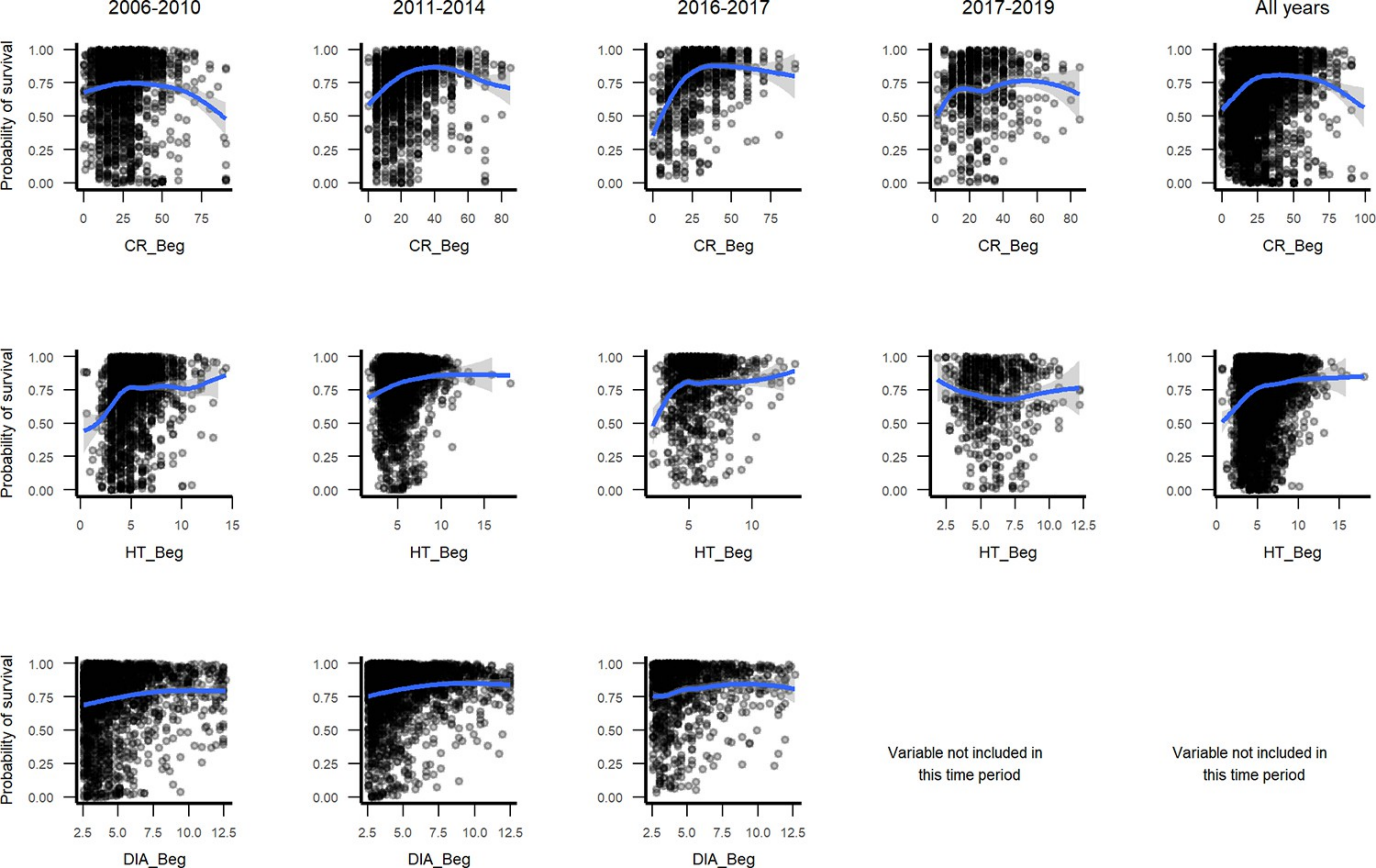
Marginal plots through time for individual tree crown ratio, height and diameter.

**Fig 5:**
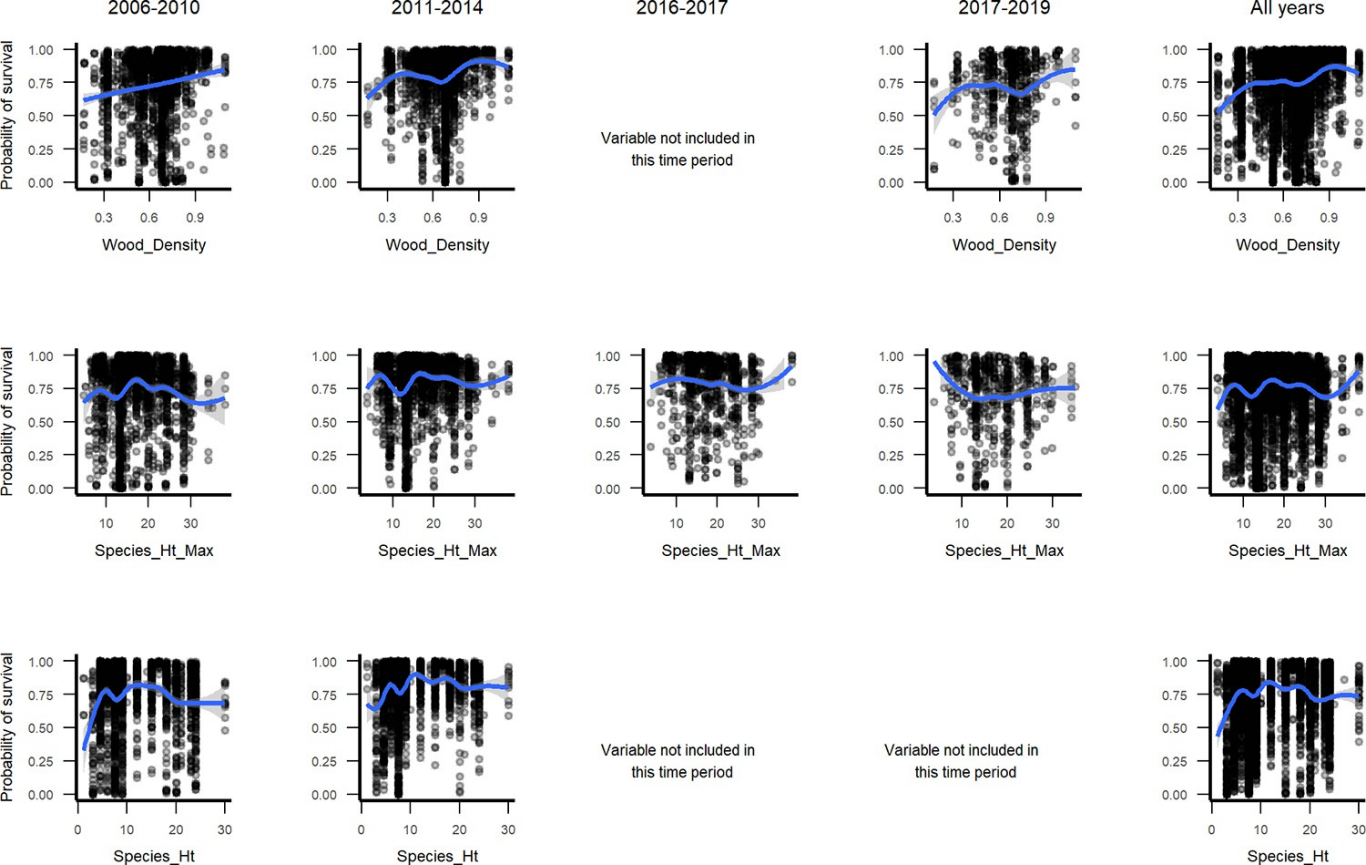
Marginal plots through time for wood density, maximum species height and typical species height.

Most important in 2006–2010 by both permuted importance and minimal depth was disturbance type in the last five years (Fig 4 in S2 File). Both rankings also highly rated wood density, northness, a topographic radiation index (rad_index_dem), red_1985, and vegetation greenness amplitude (amplitude_EVI10_14). In the period ending in 2011–2014 (pre-drought), the most important variables by both rankings included crown ratio, individual height, species height, origin, wood density, species maximum height, age and potential evapotranspiration to precipitation ratio of the four driest months (PET2Pdriest) (Fig 5 in S2 File).

**Fig 6:**
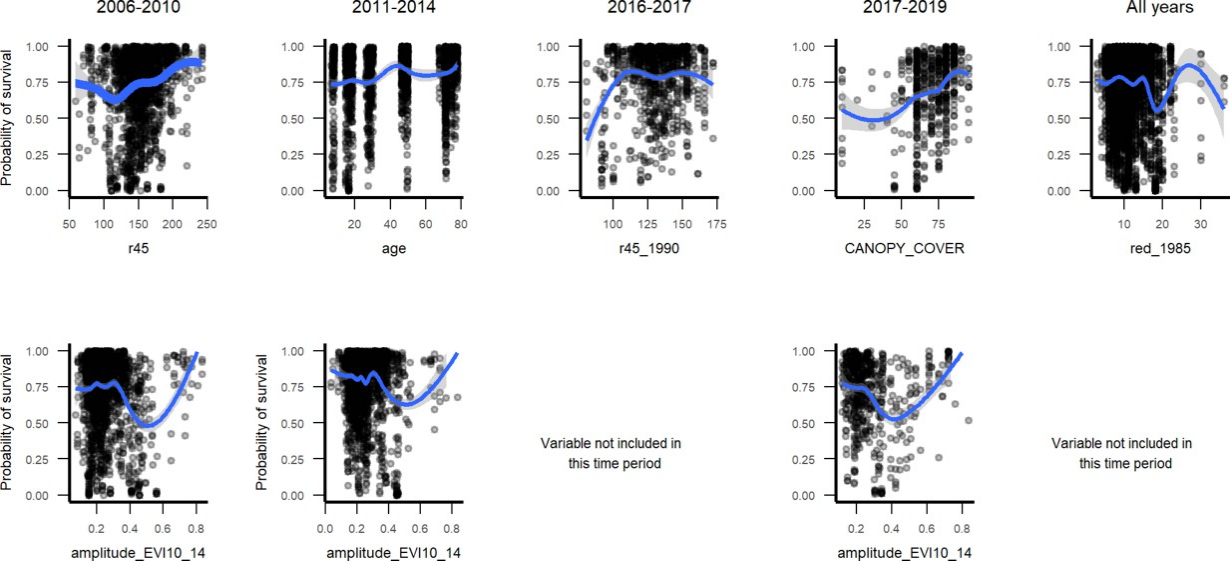
Marginal plots through time for metrics related to canopy development or deciduousness. The top row shows r45 for t2, age for t3, r45_1990 for t4a and tree canopy cover for t4b. The middle row shows the red band from Landsat Multispectral Scanner imagery dated in 1985 (red_1985). The bottom row shows greenness amplitude, derived from images dated from 2011–2014, and tree canopy cover. Tree canopy cover in 2006–2010 is modeled from remote sensing data but was field-determined for the interval ending in 2017–2019.

**Fig 7:**
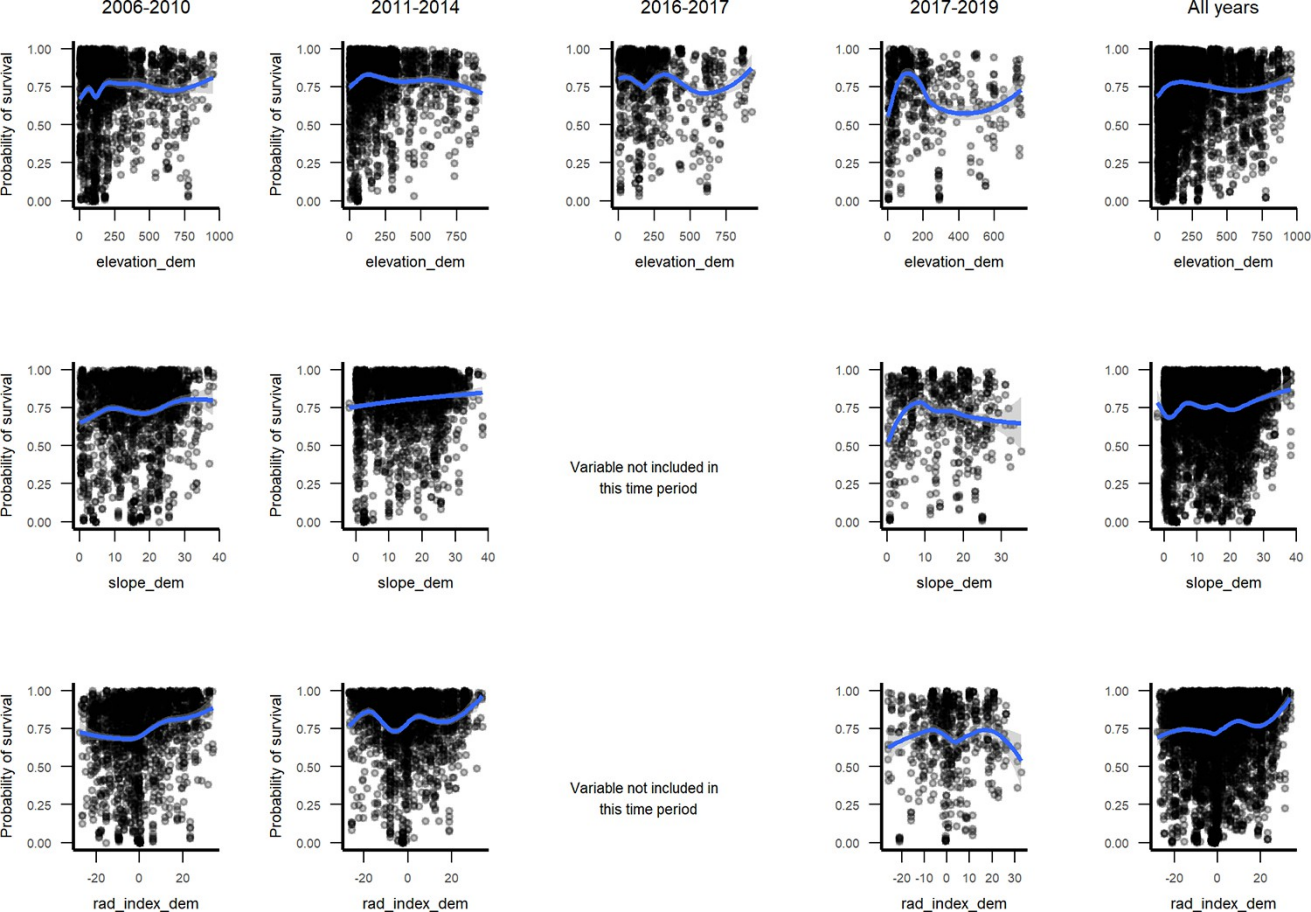
Marginal plots of elevation and slope through time, which had high rank for the interval ending in 2017–2019 (t4b) but lower rank otherwise.

The surveys in 2016–2017 (t4a) and 2017–2019 (t4b), at the end of the drought and after the hurricanes, respectively, had fewer samples, most from mainland Puerto Rico. For the survey spanning the drought, crown ratio and PET2Pdriest were most important by both minimal depth and permuted importance (Fig 6 in S2 File). Stand size, PET2Pannual the infrared to shortwave infrared ratio from the 1990 Landsat composite (r45_1990) and eastness were also among the most important variables. Post-Maria/Irma, in 2017–2019, crown ratio, elevation, conspecific small trees of native evergreen non-N-fixing species (consp_SmTr1NE0Nba), canopy cover, and wood density were the most important variables in both rankings (Fig 7 in S2 File).

### Marginal plots of survival probability

**Fig 8:**
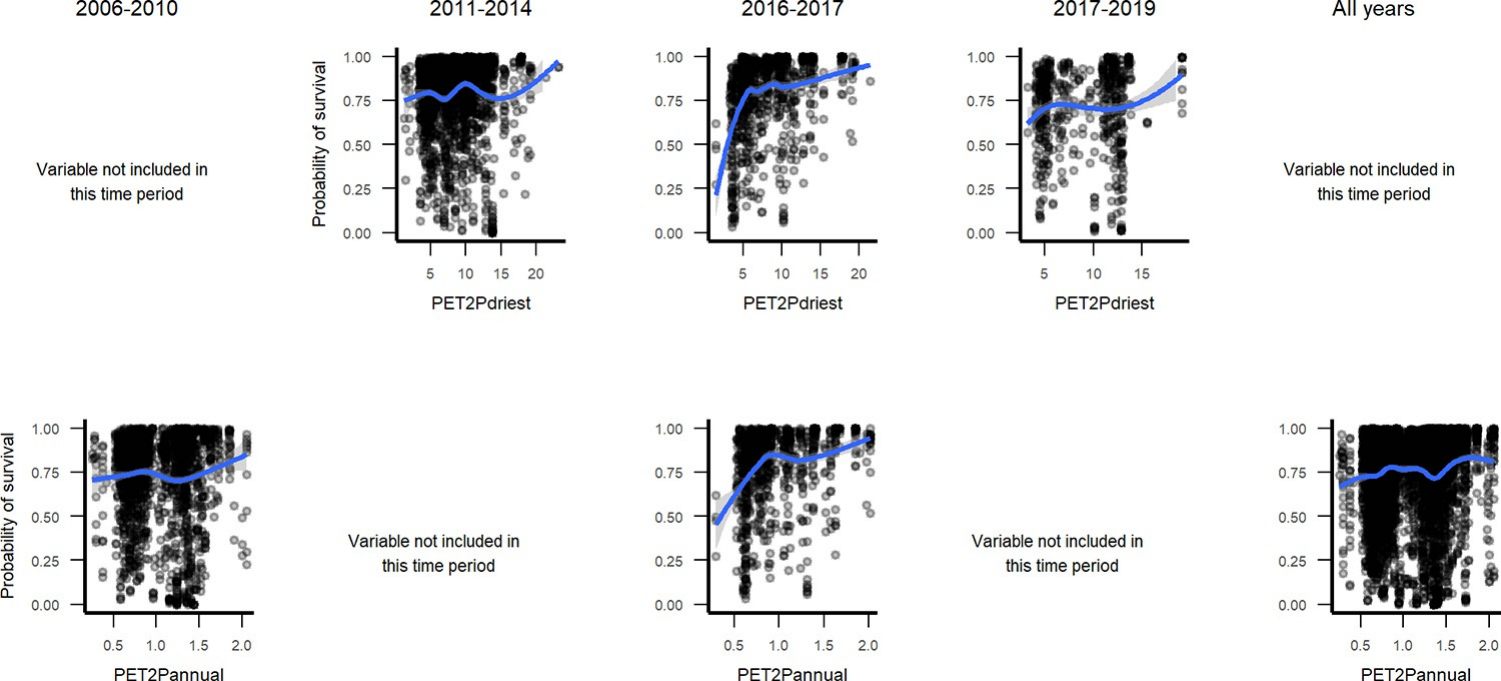
Marginal plots through time of long-term potential evapotranspiration to precipitation ratio for the year (PET2Pannual) and the driest quarter (PET2P driest). This index is inversely related to aridity.

**Fig 9:**
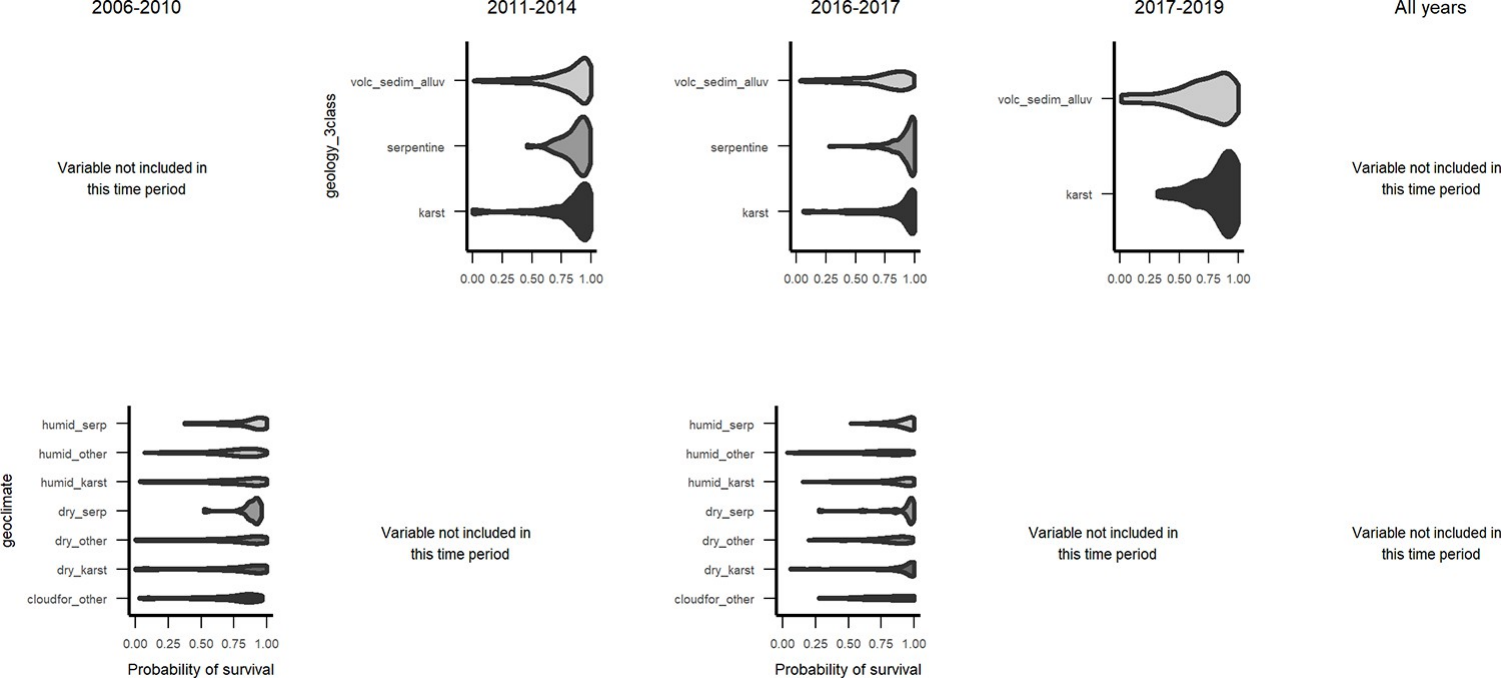
Marginal plots of geological substrate and geoclimate zones through time. Class names are in see Table 3.

**Fig 10:**
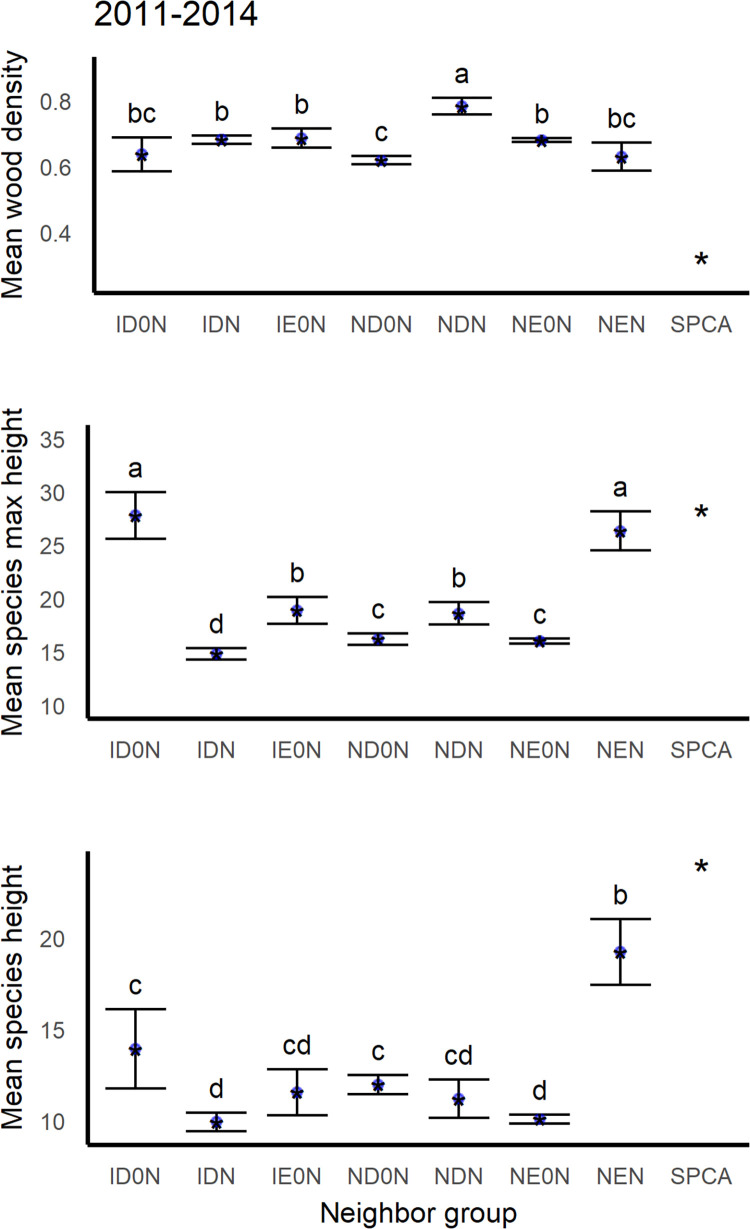
Mean wood density, maximum species height and typical species height of species in different neighborhood functional groups in the inventory data from the years 2011–2014. Functional groups are listed in Table 2.

Marginal plots depict changes in survival probabilities as predictor variables change while accounting for all other observed variable combinations. Most marginal plots agree with univariate tests, particularly for the top predictors as ranked by minimal depth or permuted importance (Figs 1–10 in S3 File). At the same time, they show important patterns. Evaluating individual tree variables (Fig 4), they show small tree survival peaking concavely at crown ratios of 25 to 60%. Survival increases asymptotically to an average individual height of ~5 m. However, shorter individuals survived best during the hurricanes in 2017. When diameter was selected, marginal plots suggest a slightly increasing, slightly concave relationship with survival. Crown ratio and tree height most often ranked highly in models (Figs 3–7 in S2 File).

Marginal plots for continuous species traits, when viewed across intervals (Fig 5), show survival increasing with wood density. Taller maximum species height corresponds to increasing survival, but not during the hurricanes in 2017. Then, shorter species survive better. Species with moderately tall maximum height survive better in t2. Species with intermediate typical height survive longer. Univariate tests and marginal plots also indicate that native species survive at higher rates than introduced ones (Fig 2, Figs 2–4 and 9, 10 in S3 File).

Marginal plots for disturbance types depict mortality rates as lowest with no disturbance in the last five years for the all periods model and highest for multiple recent disturbances (*e*.*g*., wind_rain_fire), fire, human causes, unknown causes, or grazing (Figs 1, 2 and 9, 10 in S3 File).

Survival probabilities in marginal plots changed as expected with important satellite image variables (Fig 6). Indicators of more developed forest canopy correspond to higher survival for the bulk of observations, while indicators of disturbance intensity or recency correspond to less survival. Meanwhile some indicators point to higher survival in some dry deciduous stands. For example, for interval t2 that began after the hurricane in late 1998, the survival probabilities trend upward with the Landsat near infrared to shortwave infrared ratio (r45). In 2011–2014, survival increased with stand age. In the drought interval, survival increased with r45_1990 and declined with swir1 (for the latter, see Figs 5, 6 in S3 File). For the interval spanning the hurricanes in 2017, less canopy cover corresponded to higher mortality. For the bulk of observations in the all-periods, t2 and t3 models, survival declined with red_1985. At the same time, convex curves show high survival in some dry deciduous stands, which correspond to high at the largest differences between maximum and minimum greenness (amplitudeEVI10_14). We note that the marginal plot for canopy cover across all intervals points to a small peak in survival just before canopy closure begins at about 25% canopy cover (Figs 9, 10 in S3 File).

**Fig 11:**
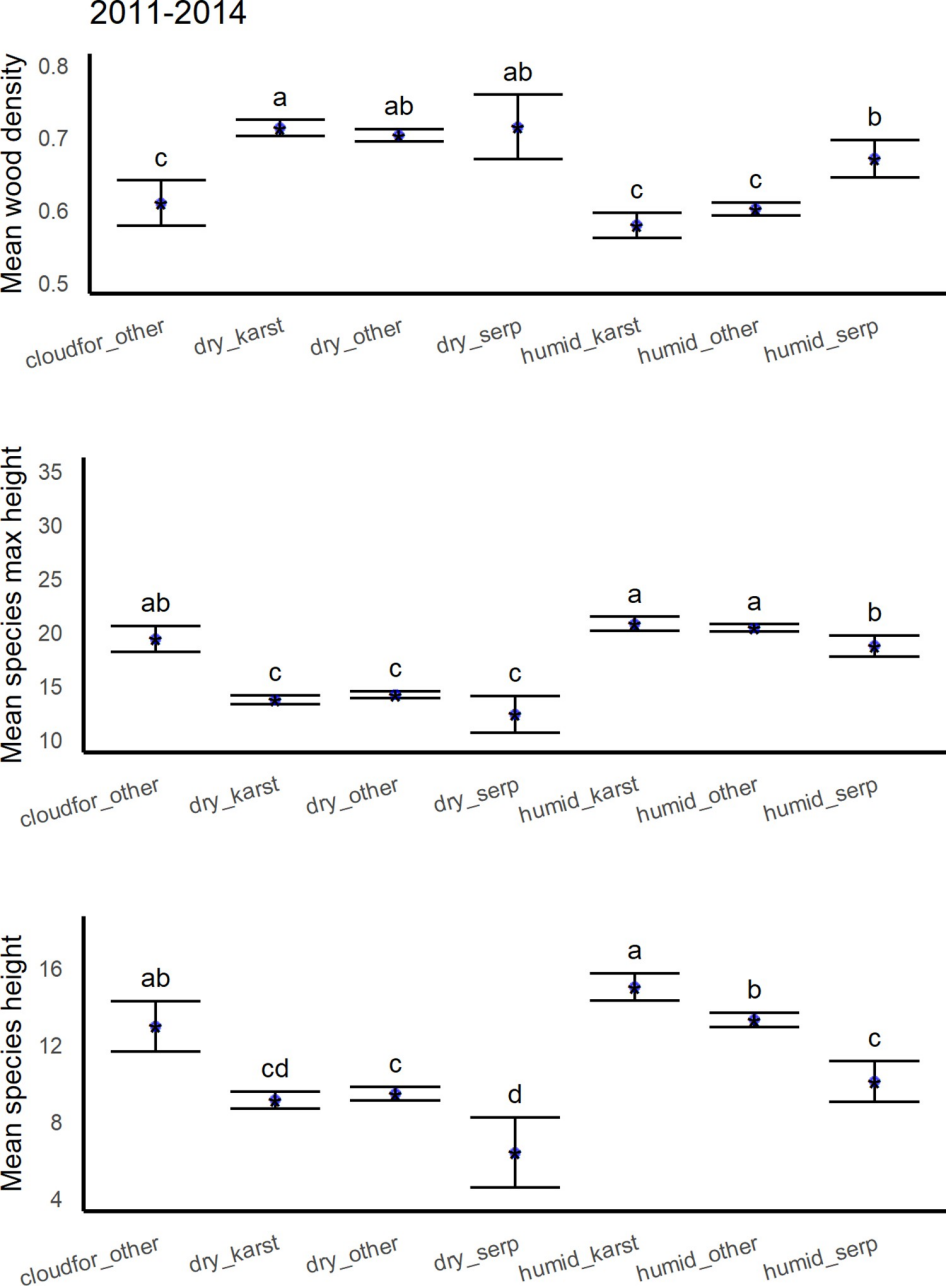
Mean wood density, maximum species height and typical species height of species in different geoclimatic vegetation zones (see Table 3) from the years 2011–2014 (cloudfor_other is cloud forest, dry_karst is dry forest on limestone substrate, dry_other is dry forest on other substrates, dry_serp is dry forest on serpentine substrate, humid_karst is moist to wet forest on limestone substrate, humid_other is humid forest on other substrates, humid_serp is moist to wet forest on serpentine substrates).

Marginal plots for relationships with topographic variables (Fig 7 and Fig 11 in S3 File), suggest that north-facing slopes and topographic positions with higher radiation, higher elevation and steeper slopes had higher survival over all years, but not during the interval spanning Hurricanes Maria and Irma. Then, survival was lowest at coastal elevations, peaked at about 100 m asl, declined for mid elevations and increased for higher-elevation cloud forests, above about 750 m asl. As for climate and geoclimate, marginal plots show increasing survival with drier climate (Fig 8), and for serpentine or karst substrates as compared with alluvial or other volcanic substrates (Fig 9). The increases in survival with drier climate with were steeper during the interval spanning the drought.

## Discussion

### Trends in neighbor functional groups and implications

The higher overall island-wide mortality rates in intervals associated with hurricanes agrees with studies of submontane and montane forests in Puerto Rico and Jamaica, respectively [53, 119]. They also help explain the short-term net increases in island-wide biomass of tall or light-wooded species groups (Fig 1). Both tall stature and light wood are associated with fast growth [121], which permits species to quickly fill canopy gaps created by tree damage or mortality from hurricanes. The island-wide increases in the biomass of fast-growing species also add perspective to previous findings that *S*. *campanulata* and other fast-growing species do not survive hurricanes as well [124, 125]. The groups with biomass increases included large trees of the light-wooded and tall *S*. *campanulata* (SPCA) and small trees of native N-fixing evergreen species, which on average are also tall (Fig 10), even though, as expected from past work [126], denser-wooded species were more likely to survive (Fig 5). *S*. *campanulata* biomass also increased island-wide after Hurricane Georges (Fig 1). Its biomass trended downward otherwise. In contrast, the biomass of native non-N-fixing evergreen species trended upward island-wide from 2001 until before the latest hurricanes.

Tree mortality and stem turnover drive losses of aboveground live biomass in tropical forests [23, 127]. Modeling in French Guiana suggests that long-term increases in forest disturbance could reduce forest ecosystem services by reducing forest height, biomass, and leaf area [128]. Findings in Amazonia that large-scale disturbances like storm events affect species composition suggest that more intense events could affect tropical forest resilience to extreme climate [16]. At the same time, in Amazonian forests drought may favor species with denser wood, which grow more slowly [8]. The Puerto Rico-wide biomass trends here are consistent with an outlook where stronger or more frequent hurricanes will favor fast-growing species early during hurricane recovery, some with lighter wood that store less carbon per unit of biomass and survive at lower rates. But they also show the decline in fast-growing species during later intervals while small trees of denser-wooded trees survive hurricanes better. In sum, drought and hurricanes might favor species with different traits in the short term. The future frequency of extreme events is projected to increase [129, 130] but is uncertain, so the outcomes of climate change for forest composition, carbon dynamics and ecosystem services remain highly uncertain.

### Overview of the multiscale factors important to tree survival rates

The RF models illustrate how predictors of small tree survival span scales of space and time, as they include individual tree, species trait, stand disturbance and environmental variables, and the predictors, or their relationships with survival, can change somewhat through time. At the individual scale, taller trees with larger crown ratios of 25–60% survive better (Fig 4). At the species scale, native species and species with denser wood, taller maximum heights, or intermediate typical heights survive better (Fig 5, Figs 2–4 and 9, 10 in S3 File), but severe events may change these patterns. At site and landscape scales and over time, disturbance-related variables, including disturbance type (Fig 5 and Figs 1, 2 and 9, 10 in S3 File), and variables indicating an older or less damaged canopy (Fig 6) led to higher survival rates. Otherwise, all else equal, trees survive longer in environments with slower growing conditions (drier or edaphically dry, or cloud forest later in recovery) (Figs 8, 9 and Fig 12 in S3 File), which agrees with studies showing drier or edaphically drier tropical forests have lower mortality rates [8, 11, 38]. And as we discuss below, covariation between species traits and landscape factors likely also affects which combinations of species traits and landscape factors are most important for predicting survival.

### Changes in importance of driving variables through time

The changes in variable importance linked to mortality seem to reflect regional disturbances, with earlier intervals (t2, t3) reflecting recovery from past large-scale deforestation and a hurricane in 1998, with continued effects of that hurricane (a recovery phase). A regional drought phase (t4a) followed, and then the data span a severe hurricane phase (t4b). Topographic variables are an example. Elevation and slope were top predictors for the interval spanning hurricanes in 2017 (t4b) (Fig 7 in S2 File). They show low survival for coastal and moderate elevation forests (Fig 7), agreeing with work showing the extensive damage in those areas across mainland Puerto Rico [14, 15, 131]. Canopy cover was also important then, with low canopy cover corresponding to low survival (Fig 6), reflecting greater hurricane damage. Survival increased with eastness (t4b), offset Hurricane Maria’s southeast approach (Fig 11 in S3 File). In t2, survival increased with northness, offset from the generally eastward path of Hurricane Georges, and with radiation (Fig 11 in S3 File and Fig 7), agreeing with review findings that tropical forest recovery benefits from moderately increased solar radiation [132]. During extreme drought, marginal plots depict steeper increases in survival with drier climate than during other intervals (Fig 8). Also during drought survival increased slightly at high values for eastness, facing trade winds (Fig 11 in S3 File).

Trait importance or influence also varies slightly through time. The direction of the relationship between survival and height, including both individual and species maximum height, changed in the hurricane interval (Figs 4 and 5). Then, species with shorter maximum height and shorter individuals survived better. During other times, shorter individuals survived at lower rates, and species with shorter maximum height survived slightly less or similarly to taller species. Note that species typical height is shorter for the average dry forest species on any substrate, and species maximum height is shorter for both dry forests on any substrate and for humid forests on serpentine substrate (Fig 11). Past work suggests that dry forests in the region may benefit from hurricane related rainfall [15, 133]. In addition, leaf thickness, species origin and mycorrhizal group appeared in RF models for earlier, recovery periods but not during intervals spanning extreme drought or severe hurricanes (Figs 3–7 in S2 File).

Denser wood and serpentine geology or geoclimate consistently predicted longer survival, and marginal plots for these variables and crown ratio are fairly consistent (Figs 4, 5 and 9). The link between denser wood and higher survival was expected and resulted even though the wood density estimates were from different sources or few samples. Wood density is correlated with a growth-survival trade-off, in which light-wooded species grow faster in high resource environments but die at higher rates [49, 134–136]. Wood density can vary within species and individual trees, and wood density estimates from a few small core samples are likely imprecise [137–140]. Considering the consistency of this variable for modeling tree survival, more and better estimates of the wood density of tropical tree species are warranted.

### Species trait-to-landscape growth-survival trade-off

Most of the species’ traits and landscape factors associated with higher survival are known to be associated with slower growth or reduced mortality rates and vice versa. Factors affecting tropical tree growth rates also operate over many scales [136]. Species-level average diameter growth may not always predict species mortality rates [41]. However, together the results in this study portray a species trait-to-landscape, multiscale trade-off between traits and places associated with a tendency for faster growth *vs*. those linked to higher survival. Serpentine or dry forest species have significantly denser wood and are shorter (Fig 11), indicating slower growth rates. In marginal plots, RF models predict higher individual survival on edaphically dry karst or serpentine geology and in drier conditions (Figs 8 and 9). Differences in biomass among forest age classes by geoclimate in Puerto Rico generally suggest slower growth [66, 141]. Results here also predict higher survival with stands that are less disturbed or less recently disturbed and have a better-developed canopy. A mature or, less disturbed canopy implies shadier conditions for small trees compared with recently disturbed or deforested conditions, and growth would be slower, as indicated by the bulk of observations in top-ranked remote sensing metrics (Fig 6).

A shallower water table corresponded to higher mortality in univariate tests (Fig 2 in S2 File). This finding agrees with results from Amazonian forests, where shallow water table forests have higher mortality (except with mild drought) [11]. There, lower forest productivity and biomass are associated with an excess or deficit of water availability, as determined by the combination of climate and edaphic conditions [42], also suggesting a landscape-level trade-off between higher survival and lower growth with harsher growing conditions. Depth to water table did not appear in RF models, and the frequency of observations in shallow water table forests is comparatively low. However, raw data suggest the possibility that mortality in shallow water table forests (depth to water table <50 cm) was relatively lower in the drought interval than other intervals (Fig 10 in S2 File).

Drier conditions correspond to higher survival rates according to marginal plots of potential evaporation to precipitation ratios (Fig 6, S3 File). But other work shows that severe drought may escalate mortality rates in seasonally dry forests more than in humid forests [4, 10, 38, 142]. During the drought period in this study, moist and submontane wet forests on alluvial and extrusive volcanic substrates had the highest mortality rates, as in other intervals. They dominate the central part of the island, where the drought was most severe. Further, much of the dry forest on mainland Puerto Rico occurs on fast-draining karst or serpentine substrates and in old stands, suggesting that the trees in these dry forest zones are particularly drought-tolerant [143]. To better characterize Caribbean dry forest resilience to drought, future research could incorporate drought severity into analyses of tree survial.

### Tree height and crown ratio

With tree height and crown ratio being important in RF models, crown ratio being at the top of model trees (smallest minimal depth), and canopy position and crown ratio having large effect sizes, results strongly support tree demography models driven primarily by light availability and stand disturbance [61, 144–147], as disturbance-related variables are also important. Results also support recent interest in calibrating or validating forest demography models with lidar metrics of canopy structure [6, 34]. Lidar profiles foliage volume. Crown ratio indicates tree vigor, growth potential and light environment and together with height is a foliage profile. While survival increases overall with increasing crown ratio, marginal plots suggest that small tree survival peaks at crown ratios of 25 to 60% in a concave curve (Fig 4). Tree diameter is more commonly available from inventory data. As a result, most demography studies use one of the following to less directly index tree light environment: tree diameters of the response tree and surrounding trees [17, 39, 52, 58, 62], metrics of light exposure or growth scaled from diameters to heights and sometimes calibrated with lidar [44, 144, 148–150], or previous diameter growth [44, 45, 50, 151, 152]. Tree diameter may gauge tree size better than height alone for large trees and appears with lower rank in some models. It has a power law relationship with crown size, while height increases asymptotically with tree diameter [147, 153, 154]. But for small trees, these results suggest that canopy profile measurements like crown ratio might improve mortality models. A question related to the crown ratio measurements we test here is the extent to which they represent storm-related tree damage that leads to mortality. The consideration is important, because lagged mortality from damage to tree crowns, trunks or leaves, from a variety of agents, is an important risk factor for mortality [19].

### Tree species stature, wood density and species origin

The RF models support wood density and tree species stature as important demographic indicators [43, 52, 134, 136, 155], as they are the most important species traits other than species origin. Differing here from past work is that these inventory data span dry to cloud forests, deep alluvial to shallow karst or serpentine soil substrates and a range of land-use histories, regional events and introduced species, all within the same species pool (excepting introduced species). Insofar as wood density and species stature both rank highly, the RF models are also consistent with the concept [131, 134] that species stature has importance to survival that differs from a trade-off with traits characteristic of faster growth. That work used both a growth-survival trade-off and a trade-off between tall stature and high recruitment to represent tree demography [131, 134]. If the tendency in these results for survival to increase with wood density (Fig 5) is reflecting a growth-survival trade-off, the high rank of species height in some of the models may reflect some independent importance of species height to survival even across this varied landscape. Caveats to this interpretation are that RF models can select correlated variables (see Caveats section below), the only demographic trait we analyze is small tree survival, and wood density is not selected in the drought interval. Smaller sample size may explain that difference, or wood density may not predict drought-related mortality. It did not predict tree survival during the drought event in 2015 in Costa Rican seasonally dry forests, though specific hydraulic traits did [10].

Introduced species origin, being highly ranked in RF models for all periods, t2 and t3, points to lower overall survival in univariate tests and marginal plots (Figs 2–4 and 9, 10 in S3 File). The average introduced species has lighter wood than the average native species (Fig 8 in S2 File). The two most common neighbor groups of introduced species are the single-species group *S*. *campanulata* (SPCA) and introduced N-fixing legumes (IDN species). The low wood density and tall stature of SPCA (Fig 10), both indicate fast growth, and this species survival rate is lower than average (63–68%) except in t3 when survival was near average at 75%. Fast growth also likely contributes to its spike in biomass after the hurricanes in 2017 despite only 63% of small individuals surviving. Common IDN species include *Prosopis pallida*, *Albizia Lebbeck*, *A*. *procera*, *Erythrina berteroana*, *E*. *poeppigiana and Leucaena leucocephala*. The average IDN species has short maximum height (Fig 10) and low survival, of 57–68%, despite moderate wood density but (Fig 10), except during the drought when 78% survived. Some IDN species can have high recruitment rates, like *P*. *pallida* and *L*. *leucocephala* [11, 156, 157]. What these species and SPCA all have in common is that they are considered shade-intolerant pioneers [158].

### Mycorrhizal groups and N-fixing status

Mycorrhizal associations are not often considered in tree survival studies. In this study, trees hosting arbuscular mycorrhizae have higher mortality rates in univariate tests, while strictly ectomycorrhizal and nonmycorrhizal species have the lowest mortality rates. Despite high effect size, mycorrhizal association, together with leaf thickness, was only selected in the model from t2. In marginal plots, nonmycorrhizal (NM) and NM or ectomycorrhizal (EM) species (“NM + EM”) are most distinct. Other factors are apparently more predictive, with the largest group, that forming arbuscular mycorrhizal associations (AM), including a wide range of species. All but one of the strictly ectomycorrhizal stems are native species. They are more likely to have coriaceous or subcoriaceous leaves, and 60% of the stems are found in dry forests, cloud forests or on serpentine substrate, where growing conditions are harsher, hard-leaved evergreen species are more common and forests are older (Figs 1, 2 in S1 File). Ectomycorrhizal associations and thicker leaves are common among “resource conservative” species [159, 160] that we expect to survive longer. Still, the strictly ectomycorrhizal species in the inventory also encompass different groups. Outside of places with harsher growing conditions, twenty-five percent of the strictly ectomycorrhizal stems are *Andira inermis*, a widespread N-fixing legume. It is a bat-dispersed, deciduous canopy tree of lowland humid Neotropical forests with intermediate wood density and an average survival rate (77%). It is common in mid-successional former pastures in Puerto Rico in lowland areas [161]. In African dry forest, deciduous ectomycorrhizal legumes similarly functionally group with tall-statured tree species with intermediate mortality and growth rates [52].

The species groups used to test neighbor effects illustrate the wide range of traits of N-fixing species. The N-fixing neighbor groups include the shortest (IDN), tallest typical height (NEN) and densest-wood (NDN) neighbor groups (Fig 10). Like mycorrhizal associations, N-fixing status likely encompasses too broad of a range of species to be a top predictor in RF models in this diverse landscape, even though N-fixing species had lower survival rates.

### Neighborhood species composition

In univariate results, survival generally declines with surrounding basal area of introduced species but increases with surrounding natives, whether conspecific or heterospecific. Despite many significant univariate correlations between survival and nearby conspecifics or heterospecifics (Fig 3), though, few neighbor variables were in the RF models. Shade intolerance and self-thinning can explain many of the negative correlations between survival and conspecifics, often called conspecific negative density dependence (CNDD), for seedlings or small trees in other tropical forests [47, 162–166] and may help explain this result.

That succession and shade tolerance explain much CNDD may be true here for introduced deciduous N-fixing legumes (IDN), which as mentioned are mainly shade-intolerant pioneers. They had the largest CNDD effect sizes (Fig 3), but conspecific neighbor variables were not in RF models. Though some late successional species here are N-fixing, basal area or abundance of N-fixers declines overall with stand age in Puerto Rico forests and in many other successional tropical forests [70, 73, 167] (Fig 1 in S1 File). As specialists in early succession in temperate forests, mortality of N-fixers exceeds that of other species in Eastern US forests as they age [59].

One high-ranked neighbor variable was small conspecific native non-N-fixing evergreens (consp_SmTrNE0N) in the marginally significant RF model spanning the hurricanes in 2017 (t4b). This species group is prominent in older stands across the study region (Figs 1, 2 in S1 File) [70]. The negative arm in the convex marginal plot for this variable (Fig 9 in S2 File) is consistent with explanations for CNDD (self-thinning or negative species-specific contagion). Alternatively, lower survival at higher basal areas of small NE0N conspecifics could represent both a disturbance and habitat gradient of increasing NE0N mortality and basal areas with increasing elevation and hurricane damage. We know from past work that relative basal areas of native, evergreen, and non-N-fixing species are higher at higher elevations (Figs 1–5 in S1 File). The positive arm in the marginal plot represents high survival rates for two species in old but small-statured stands on tall karst hilltops with shallow soils, characteristic of the higher survival rates in these habitats.

A low-ranked neighbor variable in the RF models was small *S*. *campanulata* trees. This species survival is high when basal area of small conspecifics is high in the all-periods univariate test, but the effect size is small. Simple association of *S*. *campanulata* with high small conspecific basal area in disturbed areas might explain the pattern, as the high survivorship in marginal plots for this variable corresponds to low tree canopy cover. *S*. *campanulata*, with a wood density similar to balsa (*Ochroma pyramidale*) and high growth rates, spiked after the hurricanes in 2017 (Fig 1). It also resprouts strongly after hurricanes. Seedlings of native species can colonize underneath this species [168]. Still, as mentioned, seedlings of *S*. *campanulata* can be relatively shade tolerant [80]. It dominates stands across karst lands of Northwest Puerto Rico and some parts of Central Puerto Rico [70].

### Landsat phenology, multidecadal imagery and disturbance

Multiple disturbances can increase tree mortality in both additive and synergistic ways [18–21, 110]. We tested field-determined disturbance type in the last five years including multiple disturbances, and field or remotely sensed indicators of disturbance and its severity from the last five years and from before the year 2000. We did not test successive field-noted disturbances. However, we note that during hurricanes, both wind and heavy rain are associated with high tree mortality and may reduce tree stability in tropical forests. Fire may also reduce tree stability [21]. In Caribbean dry forests, fire can increase steeply after hurricanes [110]. We also did not test forest fragmentation indices as survival predictors, because most plots are connected in a few large patches. But fragmentation can increase both tropical forest fire frequency and tree mortality and damage from wind [20]. Further, fragmentation plus fire magnify tree damage and mortality from either disturbance [18]. Consequently, future research could consider testing as survival predictors successive field-noted disturbances, remote sensing indicators from previous inventory intervals, drought and storm severity and perhaps indicators of past fragmentation.

Image bands, indices and phenology metrics were among the top-ranked predictors in all models. We attribute their frequent selection to their sensitivity to disturbance severity and vegetation type at high spatial resolution. Marginal plots of remote sensing metrics point to longer survival with less disturbance, or more time since disturbance, according to expected relationships. At the same time, the metrics are sensitive to higher survival rates for some drier forests, especially during drought.

Remote sensing disturbance metrics ranked in the top nine variables by minimum depth or permuted importance (Fig 6 and S3 File). Marginal plots show increasing survival with increasing near infrared:shortwave infrared (nir:swir1, *i*.*e*., r45) ratios after Hurricane Georges (t2) and for r45 from 1990 for the drought (t4a). They show reduced survival at the brightest swir1 values in t4a. These trends likely reflect recent or past disturbance severity or type. The shortwave infrared (swir) bands are important to detecting partial forest disturbance [169]. Spectral data from long image time series can predict current tropical forest structure better than stand age alone [110, 170–173]. Band indices contrasting near-infrared with swir bands are often more sensitive than other band combinations to tropical forest disturbance, structure, successional stage, canopy openness, or selective logging [158, 160–162]. Unvegetated, unsaturated soils or burned areas reduce r45 as they are bright in swir bands; tree shadows from larger trees darken swir bands, increasing r45. Vegetation greenness increases nir bands, increasing r45.

Disturbance history may also explain declining survival for the bulk of observations with brighter MSS red band values from 1985 for the all-periods, t2 and t3 models (Figs 1–4 in S3 File and Fig 6). The Landsat red band may be preferred for detecting tropical dry forest disturbance [174]. It is also important to detecting humid forest disturbance in older, Landsat multispectral (MSS) data, as they have no swir bands [175]. Vegetation darkens reflectance in visible bands like the red band, wavelengths that vegetation absorbs, as does tree shadow. Consequently, brighter red band reflectance corresponds to shorter, sparser, or less developed forest canopies or increased disturbance.

High-ranked image metrics likely also reflect survival differences related to vegetation type. Vegetation types change over distances shorter than the grid size of climate maps. Finer-scale edaphic, topographic, or atmospheric differences change species composition. We know that the 30-m cells of multiseason Landsat imagery greatly improve distinction of deciduous and semi-deciduous Caribbean vegetation types, even when climate maps are included as predictors [109], and that Caribbean vegetation phenology [176] and physiognamy [177] can vary among geological substrates. High spatial resolution may account for survival peaks where satellite image bands or phenology metrics imply sparser, shorter, or more deciduous canopies, in drier conditions. The increases in survival at the largest values in greenness amplitudes in t2, t3 and t4b (Fig 6), and for swir1 during the hurricane interval (t4b) (Fig 8 in S3 File), are highly dense and deciduous dry forest observations, and sparse coastal dry forest observations, respectively. The largest greenness amplitudes are from Vieques, where there are extensive areas dominated by deciduous species, as illustrated in a map of deciduous and N-fixing deciduous species across the region (Figs 1, 2 in S1 File).

The satellite image composites have residual noise from differences in image dates among pixels, like atmospheric differences. Compositing methods reduce this noise but are imperfect. In addition, uncertainty in the phenology metrics rises with cloud frequency and across imaging paths. Before the year 2013, the number of clear observations from Landsat was limited in Puerto Rico, even after combining five years of images [108]. Using greenness maxima and minima and their difference reduces this uncertainty. However, acquisitions of high- and medium-resolution optical imagery are now more frequent. Given these uncertainties, more research is needed to evaluate the consistency of relationships between phenology metrics and forest dynamics across complex tropical landscapes.

### Caveats

It should be noted that the univariate tests between numeric and categorical predictors are not comparable because different variable types necessarily required different statistical tests. Moreover, in large samples it is relatively easy to find statistical significance, but that does not always equate to a meaningful difference. Thus, we chose to include effect size calculations as well as the results of the statistical tests to find biological or ‘practical’ significance. We note the tests and calculations we performed assume independence among observations, which may not be met spatially for individuals within the same plots. By analyzing the data across and within periods we ensured temporal independence as no individuals were sampled twice within the same period, and effects of repeated measures in the all-years analyses were likely trivial. Nevertheless, we admit spatial variation was not accounted for in our analyses which may lead to bias in some cases. However, the potential bias due to the correlated nature of our predictor variables was assumed to be greater risk and thus a machine-learning approach was more appropriate. Furthermore, using a machine-learning method allowed for interaction effects and non-linear relationships to drive the model without the need for any specific a priori assumptions for model formulation.

Although the random forest models perform better than using the class proportions, Kappa values were not large showing fair to moderate agreement at most between model predictions and observed survival outcomes. Machine-learning algorithms such as random forests are increasingly being used for large datasets with correlated variables due to their lack of stringent parametric assumptions. Random forests have been shown to have high accuracy and can handle interactions between predictor variables that are unknown a priori. However, the reliability of several possible variable importance measures produced by this algorithm continues to be evaluated [178–182]. For example, random forest variable importance may tend to inflate the importance of continuous variables that have more unique values [183] and may introduce bias with correlated predictor variables [180]. Nonetheless, machine-learning algorithms such as random forests prove to be a valuable tool for finding potential patterns of important variables among many possible predictors in large datasets.

## Conclusions

Top predictors of tropical small tree survival in these data, when excluding species as a predictor, are typically tree crown ratio and height, a stand or landscape variable related to disturbance or regrowth, and two to three each of species traits and other landscape factors. This result applied across tropical dry to cloud forests ranging widely in age, disturbance history, geology and topography. Other things equal, small tree survival rates are highest in older or least-disturbed forests. Survival increased fairly consistently with wood density, and it was the trait most often ranked highly.

Covariation between species traits and stand or landscape factors may affect which variables are most important when predicting survival. Wood density and survival are high, species are shorter, and stand-level growth is slower in harsher environments including dry forest on any substrate and humid forest on edaphically dry and nutrient poor serpentine substrate.

The importance of some factors, and the forms of some variable relationships with survival, can change somewhat through time, reflecting the regional system state of widespread recovery, drought or disturbance. For example, radiation index was important in intervals characterized by forest recovery. Elevation and slope were important with recent hurricanes. Some changes suggest resistance to drought or storms for landscape conditions where trees survive longer. The tendency for longer survival in drier conditions was accentuated during drought. During hurricanes, short individuals and species survived at higher rates though they survived at lower or similar rates otherwise.

The covariation between species traits, survival and landscape factors, including those related to disturbance, suggests a multiscale, species trait-to-landscape trade-off between traits and places with higher base mortality rates *vs*. those where species are adapted to slow-growing conditions and survive longer. Neighborhood composition or conspecificity seems to have low or no importance, whether neighbors are introduced or native species. Univariate correlations between survival and neighborhood composition seem related to disturbance, succession, and other habitat factors, including in the one case of an important neighbor variable. An example being high mortality in stands of some pioneer species with high conspecific basal areas.

Variables from satellite imagery can be important to predicting tree survival beyond disturbance timing. Satellite image spectra, including from past decades, phenology metrics, and potentially canopy cover, can help gauge disturbance intensity and vegetation type and have higher spatial resolution than climate maps, improving their ability to detect forests in dry to moist zones that are edaphically dry or moist.

With tree crown ratio and height being the strongest individual predictors of small tree survival, remotely sensed canopy height profiles might help predict small tree mortality in stands with uniform structure. With species height and wood density being two of the three most important species traits, they may well reflect different gradients in life history strategies. In these island forests, whether a species is native is also a strong predictor. Introduced species died at twice the rate of natives and on-average have lighter wood. After two severe storms in 2017, however, a light-wooded introduced species spiked. Light-wooded species are favored during forest recovery from hurricanes, while drought may favor thicker-leaved or other species types. If these events favor different traits, climate change influences on forest composition, carbon dynamics and other ecosystem services remains highly uncertain and may depend on the frequency and severity of extreme events.

## Supporting information

S1 File Spatial distributions across Puerto Rico and the Virgin Islands of the relative basal areas of species that are N-fixing, deciduous, deciduous N-fixing, deciduous, evergreen, evergreen coriaceous, native, introduced and endemic. Also included are maps of geological substrate, long-term annual potential evapotranspiration to precipitation ratio, elevation, Puerto Rico forest stand age in about the year 2000, stand age by geoclimate (cloud forests included with humid zones), and hurricane tracks.
(PDF)

S2 File Univariate survival rates across all periods by inventory interval and predictor variables, variable importance plots, wood density of native *vs*. introduced tree species, marginal plot for small conspecific NE0N neighbors in t4b and survival by depth to water table and geoclimate. (PDF)

S3 File Marginal plots for the top nine predictors, as ranked by minimal depth and permuted importance, for the RF models, and marginal plots across time for topographic radiation index and northness, and marginal plots across time for the cloud forest geoclimate zone. (PDF)
