# Supplementary material for: Multiscale predictors of small tree survival across a heterogeneous tropical landscape
Source: PLoS One. 2023 Mar 15;18(3):e0280322. doi: 10.1371/journal.pone.0280322 (PMC10016699; doi:10.1371/journal.pone.0280322)
Supplement: S1 File — Also included are maps of geological substrate, long-term annual potential evapotranspiration to precipitation ratio, elevation, Puerto Rico forest stand age in about the year 2000, stand age by geoclimate (cloud forests included with humid zones), and hurricane tracks. (PDF) [file pone.0280322.s001.pdf]

S1 Supporting Maps for "Multiscale predictors of small tree mortality across a heterogeneous tropical landscape"

https://doi.org/10.1371/journal.pone.0280322

Maps of Tree Functional and Species Characteristics, Geology, Climate, Elevation, Forest Age, and Hurricane Tracks for Puerto Rico and the U.S. Virgin Islands

Contents

Fig 1 in S1 Supporting. Forest Age and Basal Areas of Nitrogen-fixing and Deciduous Nitrogen-fixing Trees 1

Fig 2 in S1 Supporting. Basal Areas of Deciduous, Evergreen and Thick-leaved Evergreen Trees 2

Fig 3 in S1 Supporting. Native, Introduced and Endemic Tree Basal Areas 3

Fig 4 in S1 Supporting. Puerto Rico Geoclimate Zones by Forest Age 4

Fig 5 in S1 Supporting. Geology, PET to Precipitation Ratio and Elevation 5

Fig 6 in S1 Supporting. Distributions of Four Example Tree Species... 6

Fig 7 in S1 Supporting. Paths of Recent Major Hurricanes Affecting the Study Area 7

Fig 8 in S1 Supporting. Historic hurricane tracks over Puerto Rico. 8

Sources 9

Fig 1 in S1 Supporting. Forest Age and Basal Areas of Nitrogen-fixing and Deciduous Nitrogen-fixing Trees

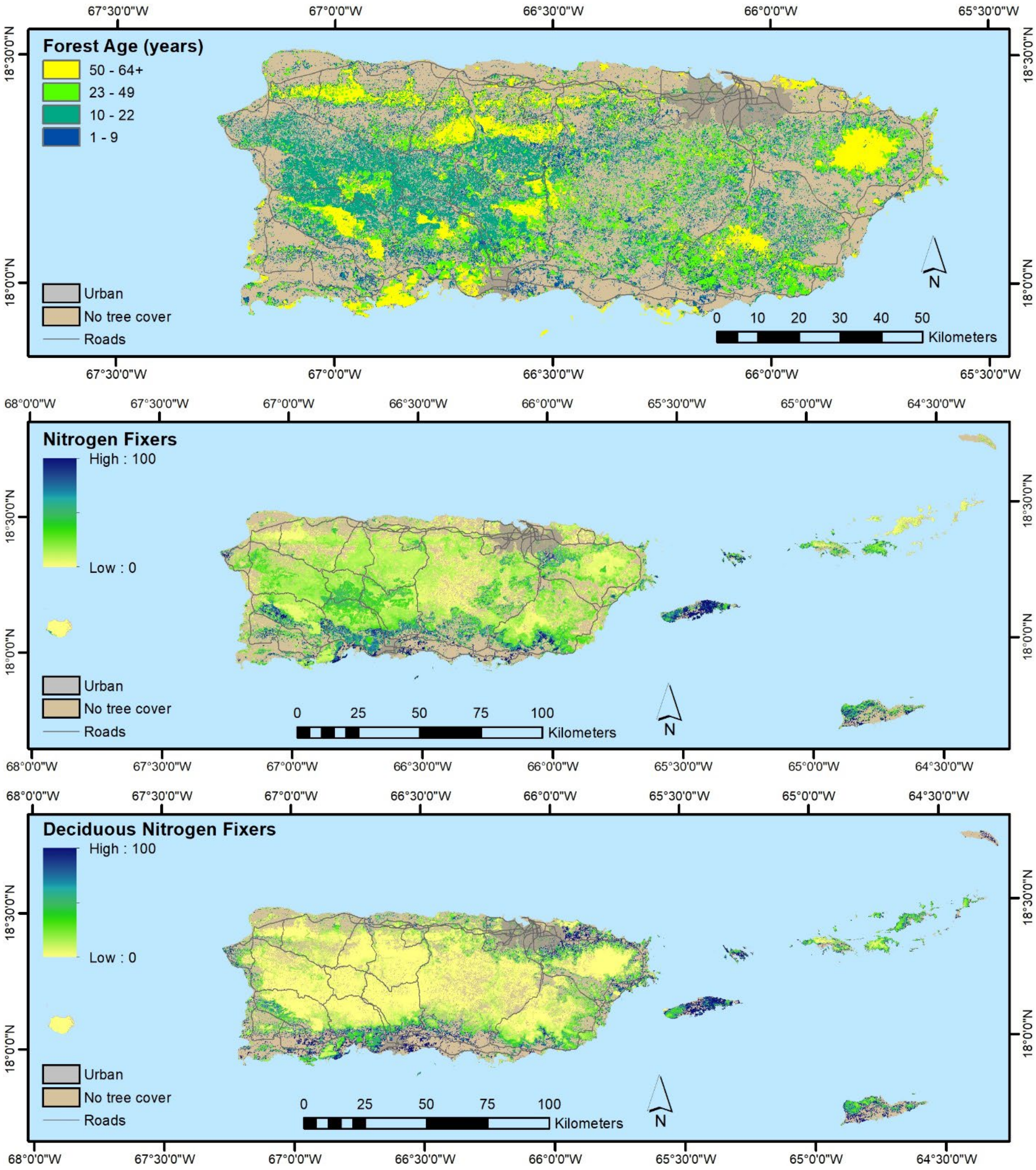

Fig 1 in S1 Supporting. Stand age in Puerto Rico circa the year 2000 (a) and relative basal areas, as mapped with Cubist regression tree models, of (b) N-fixing species and (c) deciduous N-fixing species (source: Helmer et al. 2018).

Fig 2 in S1 Supporting. Basal Areas of Deciduous, Evergreen and Thick-leaved Evergreen Trees

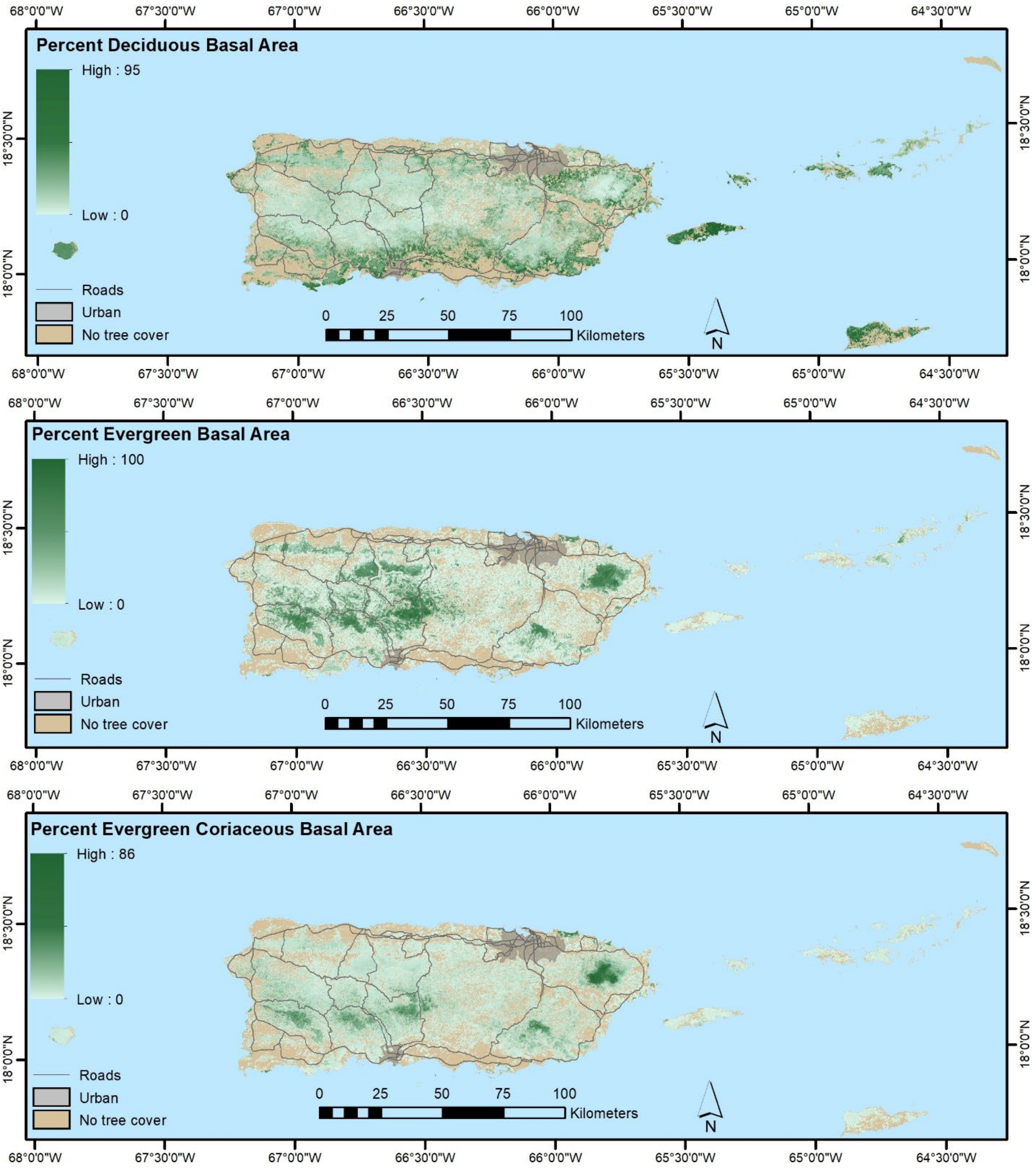

Fig 2 in S1 Supporting. Relative basal areas, as mapped with Cubist regression tree models, of (a) deciduous tree species, (b) evergreen tree species, and (c) hard-leaved (coriaceous) evergreen tree species (source: Helmer et al. 2018).

Fig 3 in S1 Supporting. Native, Introduced and Endemic Tree Basal Areas

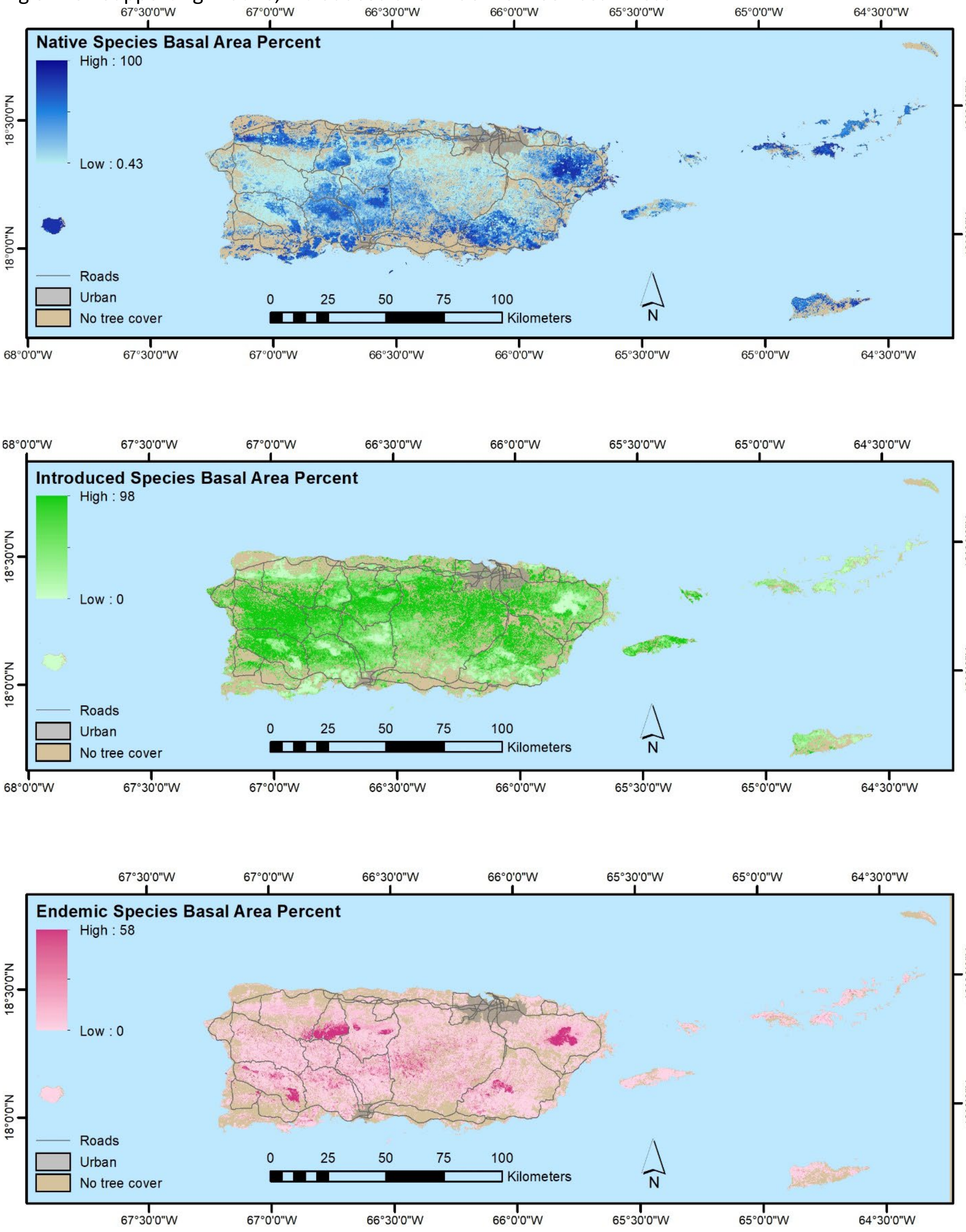

Fig 3 in S1 Supporting. Relative basal areas of (a) native species, (b) introduced species, and (c) endemic species as mapped with Cubist regression tree models (source: Helmer et al. 2018).

Fig 4 in S1 Supporting. Puerto Rico Geoclimate Zones by Forest Age

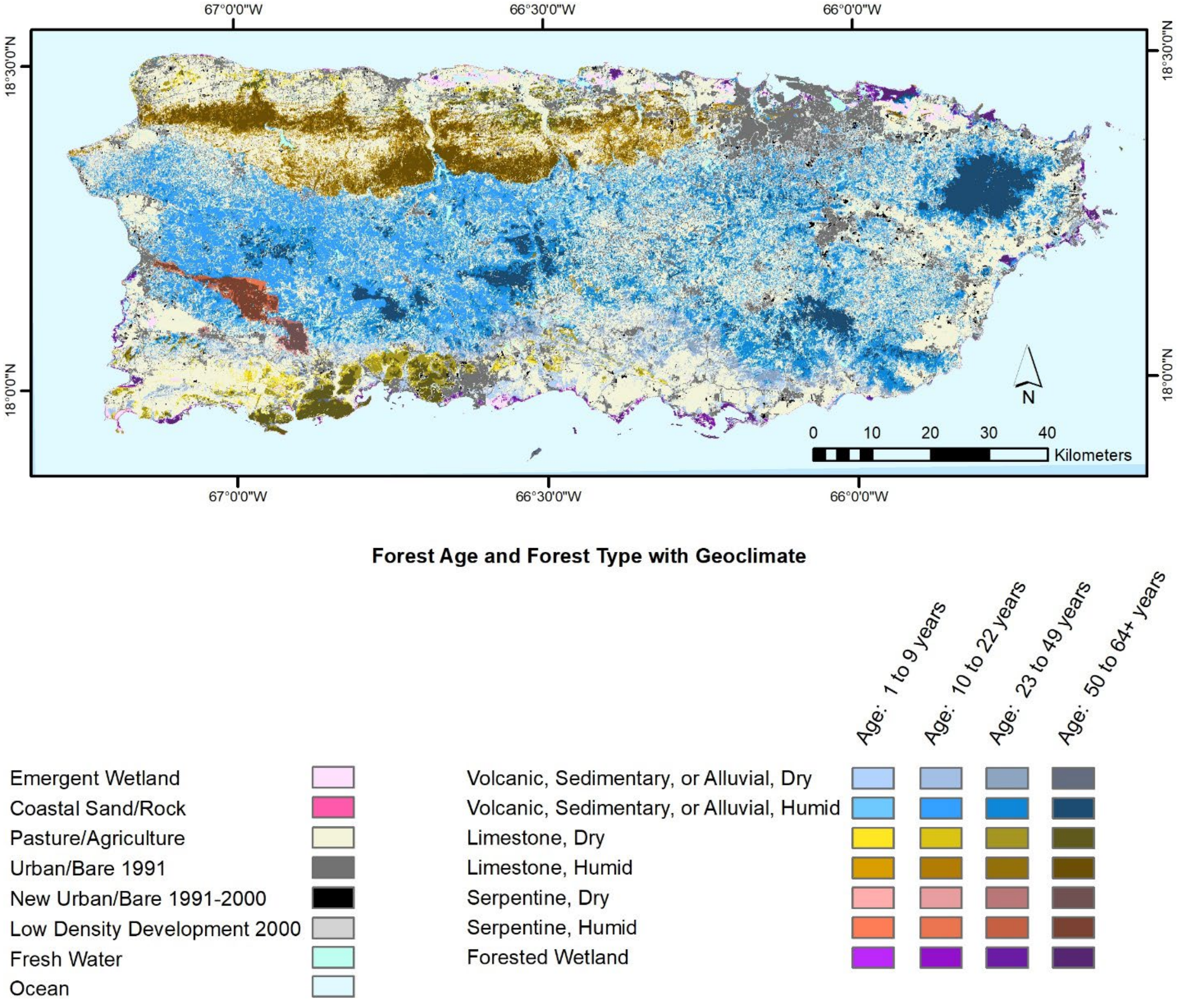

Fig 4 in S1 Supporting. Puerto Rico dry *vs.* humid zones by geology and forest age class. Cloud forests occur within fhe largest patches of the oldest forests on volcanic, sedimentary, or alluvial geological substrates (dark blue) (source: Helmer et al. 2008).

Fig 5 in S1 Supporting. Geology, PET to Precipitation Ratio and Elevation

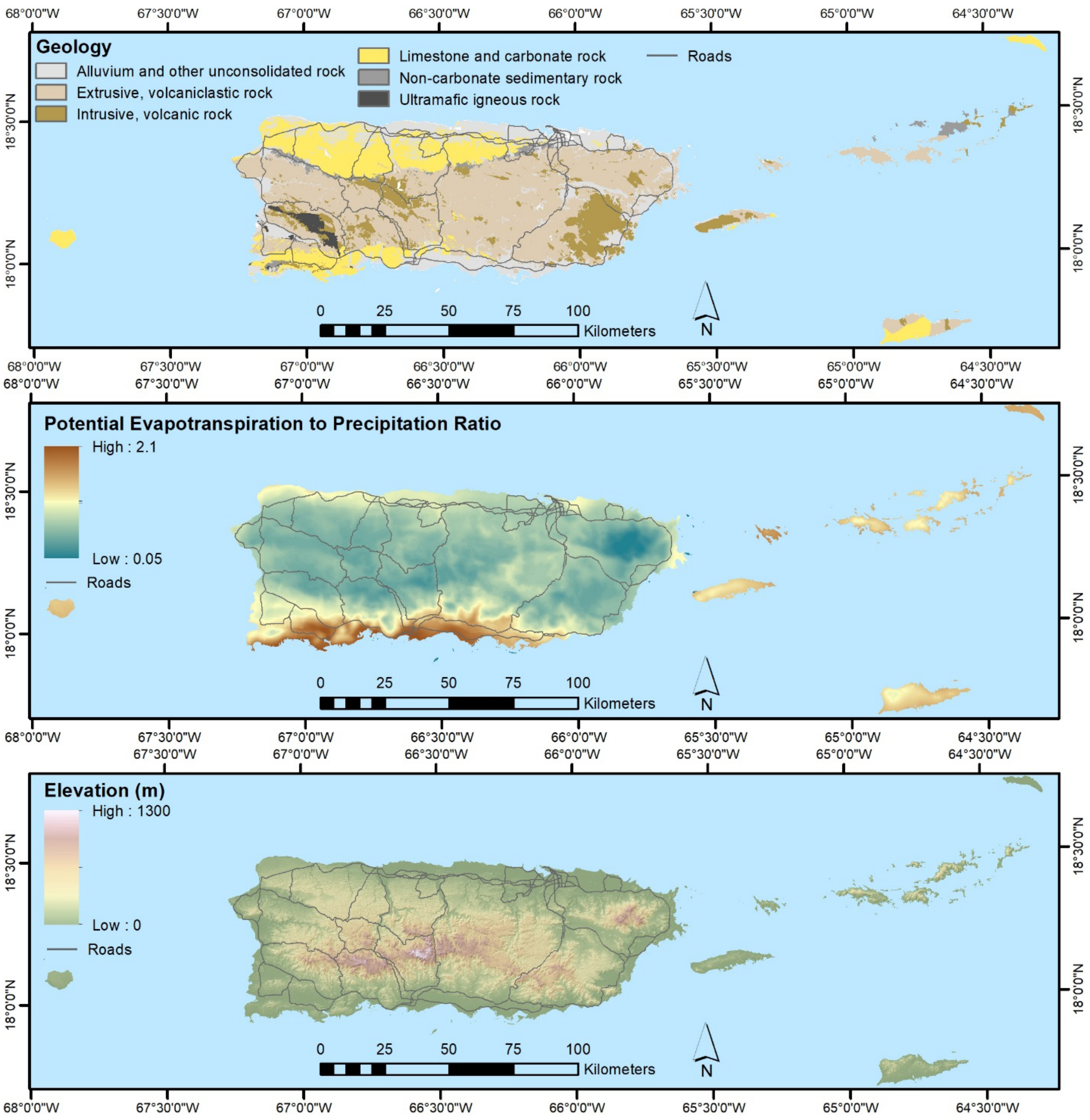

Fig 5 in S1 Supporting. Puerto Rico and the Virgin Islands: (a) geological substrate; (b) potential evapotranspiration to precipitation ratio, a climatic indicator of moisture; and (c) elevation (source: Helmer et al. 2018, https://doi.org/10.3390/rs10111724) S6 Fig. Relative basal areas of four species as mapped with PGNN modeling, including (a) a deciduous N-fixing species found in young or disturbed dry forests; (b) a deciduous native found in dry and edaphically dry places; (c) an evergreen native typical of humid karst areas; and (c) Spathodea campanulata, a common introduced species (Source: Helmer et al. 2018).

Fig 6 in S1 Supporting. Distributions of Four Example Tree Species

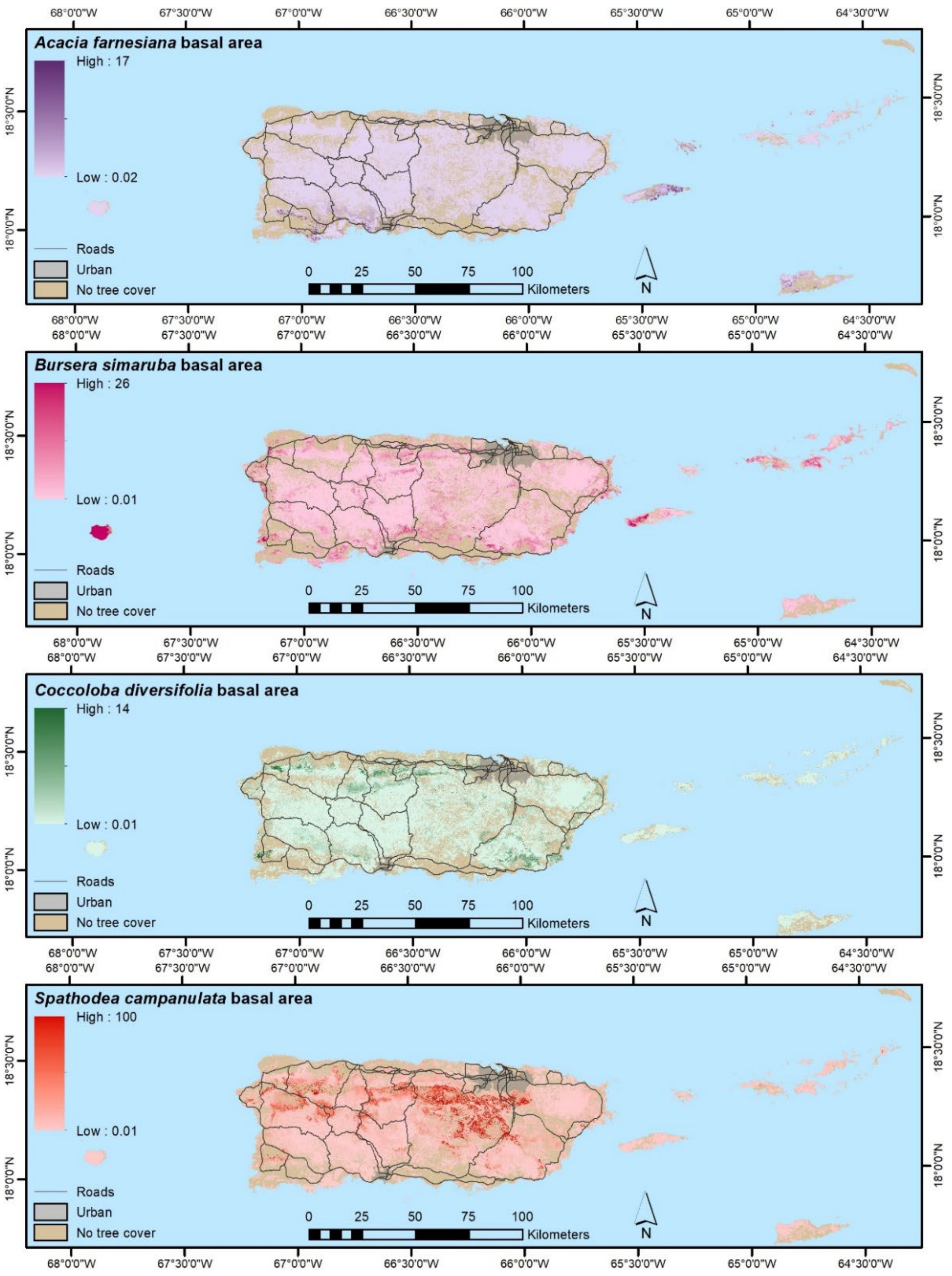

Fig 6 in S1 Supporting. Relative basal areas of four species as mapped with PGNN modeling, including (a) a deciduous N-fixing species found in young or disturbed dry forests; (b) a deciduous native found in dry and edaphically dry places; (c) an evergreen native typical of humid karst areas; and (c) *Spathodea campanulata*, a common introduced species (Source: Helmer et al. 2018).

Fig 7 in S1 Supporting. Paths of Recent Major Hurricanes Affecting the Study Area

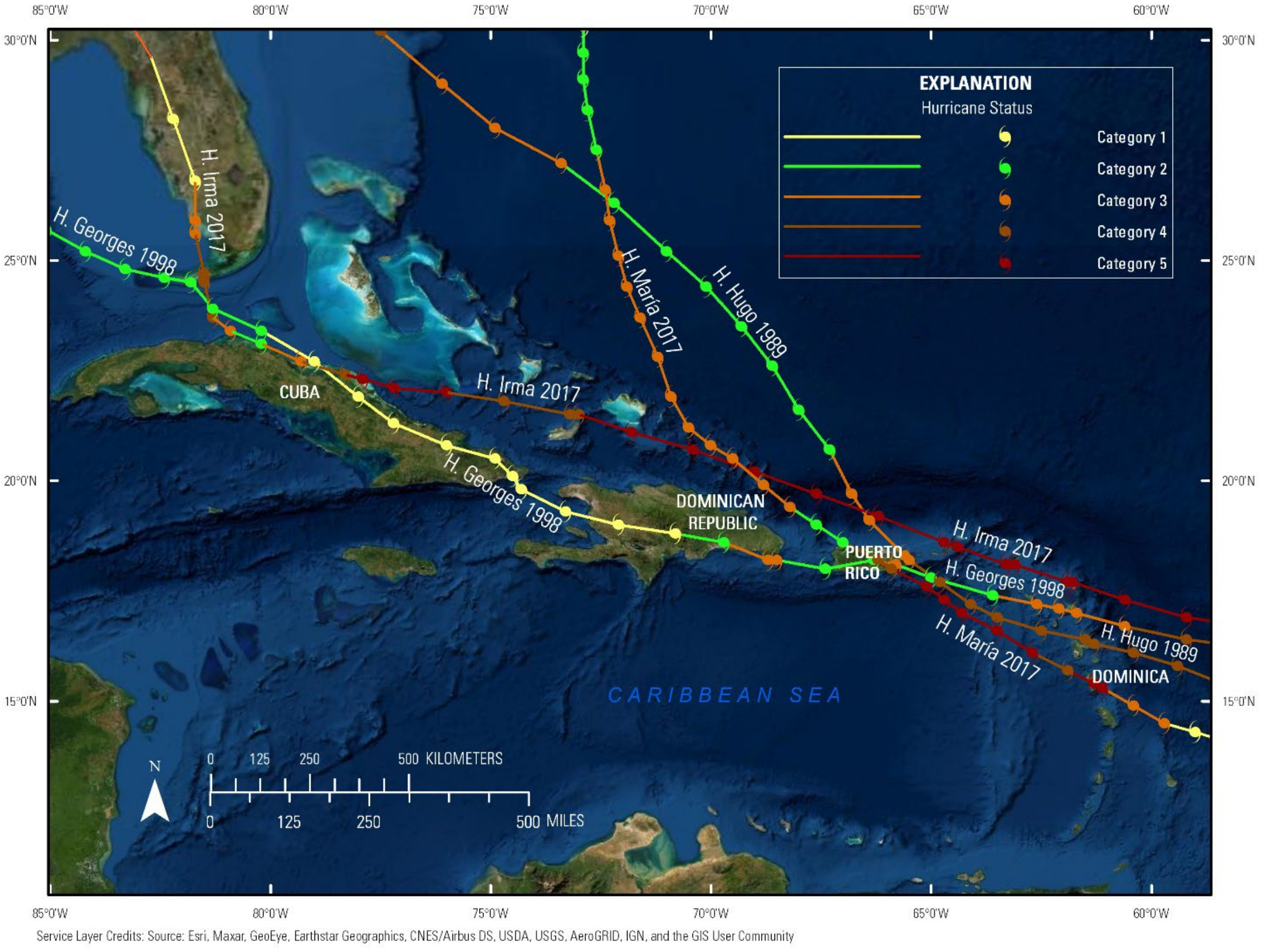

Fig 7 in S1 Supporting. Tracks for recent major hurricanes crossing the study region, including Hurricanes Hugo (1989), Georges (1989), Irma (2017) and Maria (2017) (Source: Karwandyar 2022).

Fig 8 in S1 Supporting. Historic hurricane tracks over Puerto Rico.

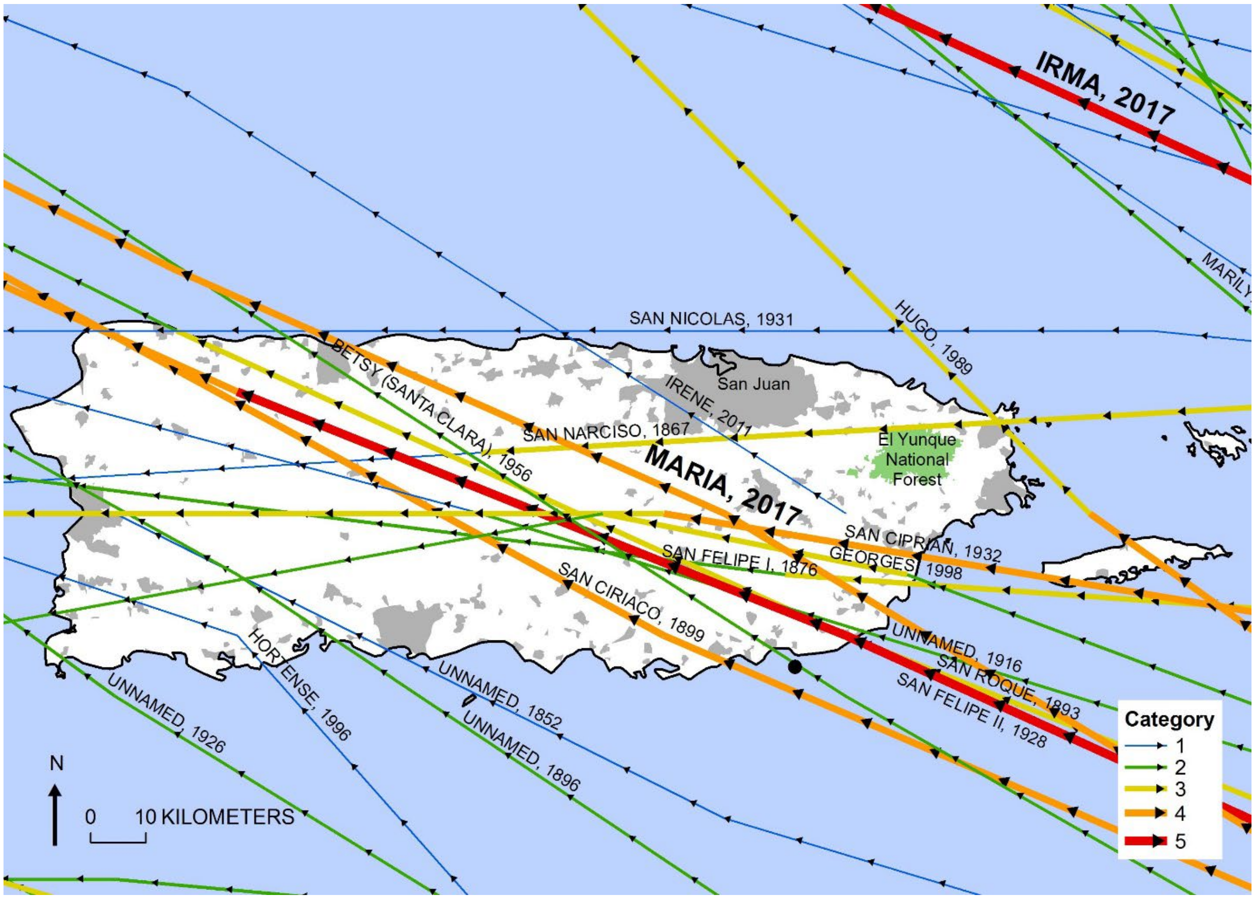

Fig 8 in S1 Supporting. Tracks of selected historic hurricanes hitting Puerto Rico (Source Murphy et al. 2012).

Sources

Helmer, E.H., Ruzycki, T.S., Wilson, B.T., Sherrill, K.R., Lefsky, M.A., Marcano-Vega, H., Brandeis, T.J., Erickson, H.E. and Ruefenacht, B., 2018. Tropical deforestation and recolonization by exotic and native trees: Spatial patterns of tropical forest biomass, functional groups, and species counts and links to stand age, geoclimate, and sustainability goals. Remote Sensing, 10(11), p.1724 (https://doi.org/10.3390/rs10111724).

Helmer, E.H., Brandeis, T.J., Lugo, A.E. and Kennaway, T., 2008. Factors influencing spatial pattern in tropical forest clearance and stand age: Implications for carbon storage and species diversity. Journal of Geophysical Research: Biogeosciences, 113(G2) (https://doi.org/10.1029/2007JG000568).

Karwandyar, S. 2022. Hurricane tracks for Hugo (1989), Georges (1998), Irma (2017) and Maria (2017). Base image is intellectual property of Esri and is used herein under license. Copyright Esri and its creator. St. Petersburg Coastal and Marine Science Center, St. Petersburg, Florida, USA (Available November 12, 2022 https://www.usgs.gov/media/images/hurricane-tracks-puerto-rico-0).

Murphy, S.F., and Stallard, R.F., eds., 2012, Water quality and landscape processes of four watersheds in eastern Puerto Rico: U.S. Geological Survey Professional Paper 1789, 292 p. https://pubs.usgs.gov/pp/1789/. Figure downloaded at https://www.usgs.gov/media/images/puerto-rico-hurricanes-map, November 2022.
