# Supplementary material for: Multiscale predictors of small tree survival across a heterogeneous tropical landscape
Source: PLoS One. 2023 Mar 15;18(3):e0280322. doi: 10.1371/journal.pone.0280322 (PMC10016699; doi:10.1371/journal.pone.0280322)
Supplement: S2 File — (PDF) [file pone.0280322.s002.pdf]

S2 Supporting Information for “Multiscale predictors of tropical small tree mortality across a heterogeneous tropical landscape https://doi.org/10.1371/journal.pone.0280322”

Contents

Fig 1 in S2 Supporting. Survival rates by time period and individual, species and stand predictors 2
Fig 2 in S2 Supporting. Survival rates by spatial predictors 3
Fig 3 in S2 Supporting. Variable importance for the all-periods model 4
Fig 4 in S2 Supporting. Variable importance for the model interval ending in 2006-2010 (t2) . . . 5
Fig 5 in S2 Supporting. Variable importance for the model interval ending in 2011-2014 (t3) . . . 6
Fig 6 in S2 Supporting. Variable importance for the model interval ending in 2016-2017 (t4a) . . . 7
Fig 7 in S2 Supporting. Variable importance for the model interval ending in 2017-2019 (t4b) . . . 8
Fig 8 in S2 Supporting. Wood density of introduced vs. native tree species 9
Fig 9 in S2 Supporting. Survival probability vs. small conspecific NE0N trees 10
Fig 10 in S2 Supporting. Survival by depth to water table and geoclimate 11

Fig 1 in S2 Supporting. Survival rates by time period and individual, species and stand predictors

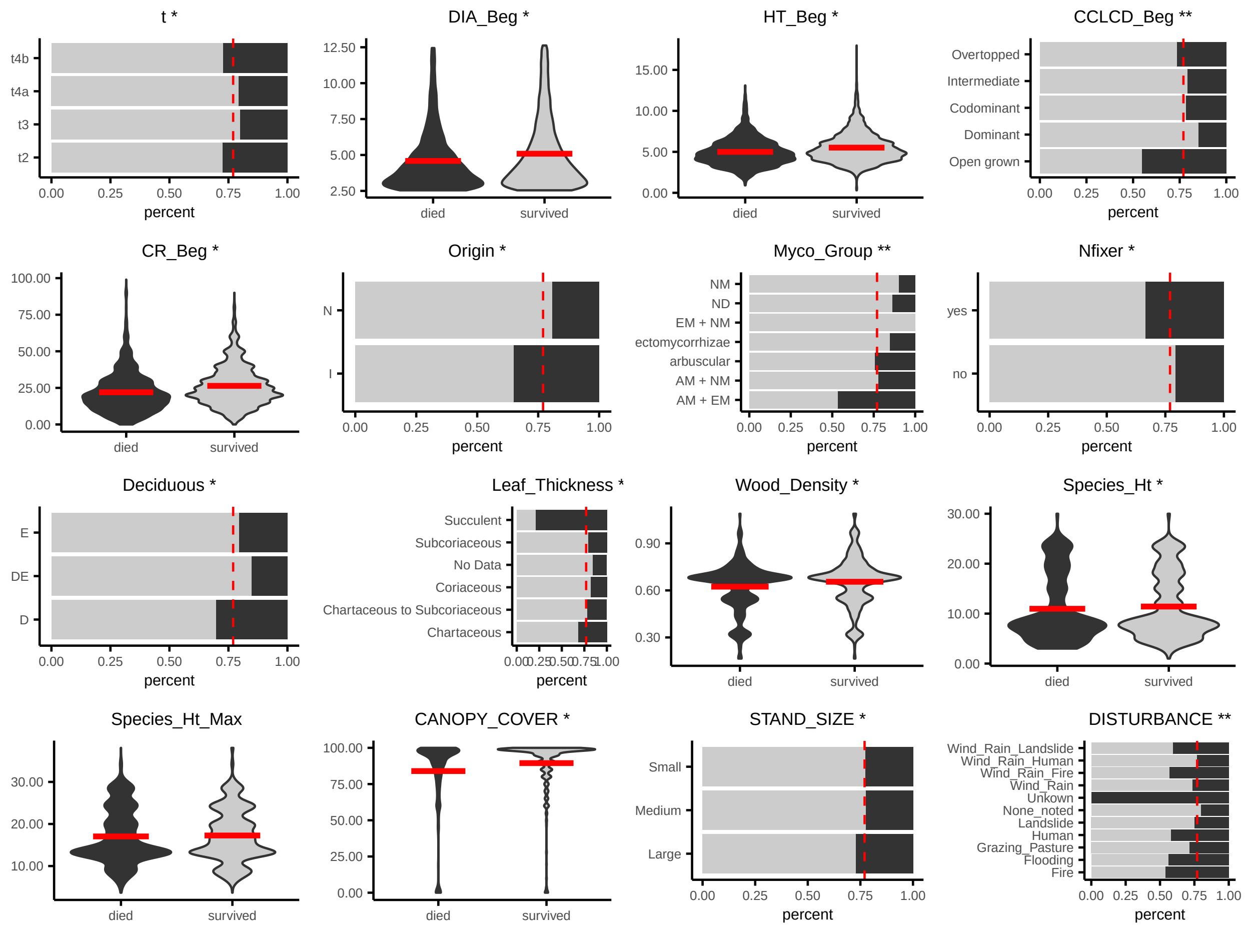

Figure 1: Raw distributions survival rates (grey is survival, black is mortality) by inventory interval (t) and individual tree, tree species trait and stand-level predictors (Table 1) across all intervals.

Fig 2 in S2 Supporting. Survival rates by spatial predictors

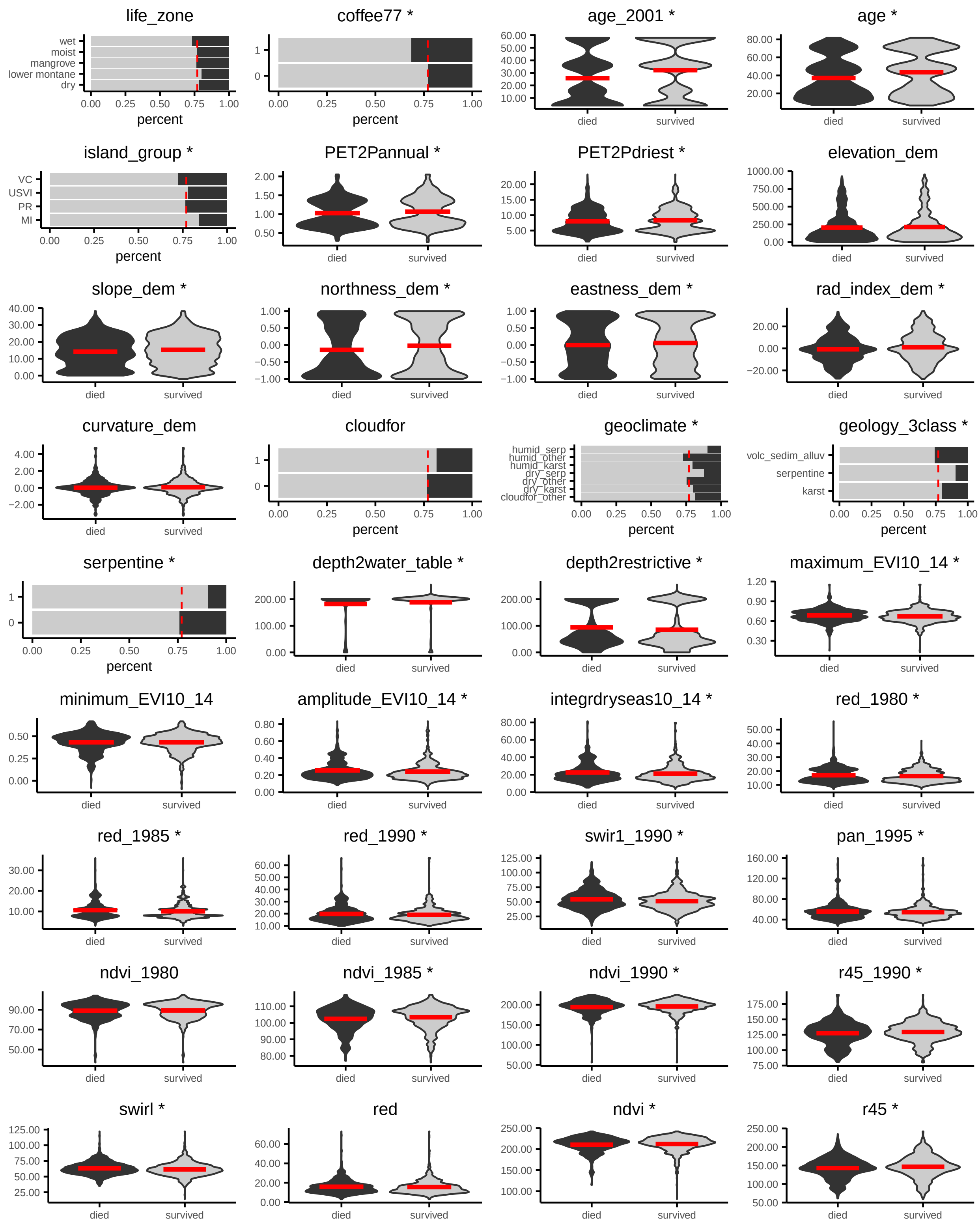

Figure 2: Raw distributions of spatial predictors (Table 3) for the all-periods model.

Fig 3 in S2 Supporting. Variable importance for the all-periods model

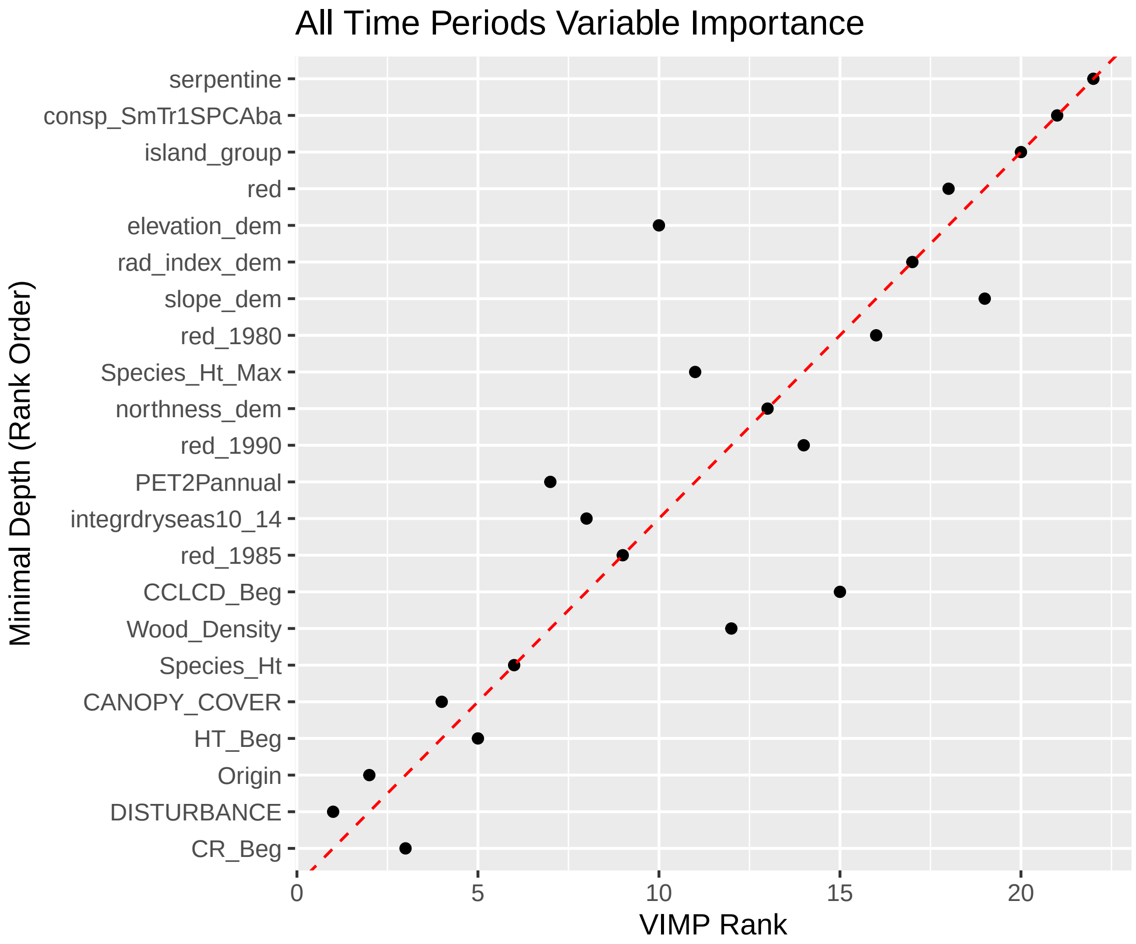

Figure 3: Ranked variable importance for all time periods combined. Ranked variable importance plot for permuted importance (x-axis) and minimal depth (y-axis) in the random forest model across all time periods.

Fig 4 in S2 Supporting. Variable importance for the model interval ending in 2006-2010 (t2)

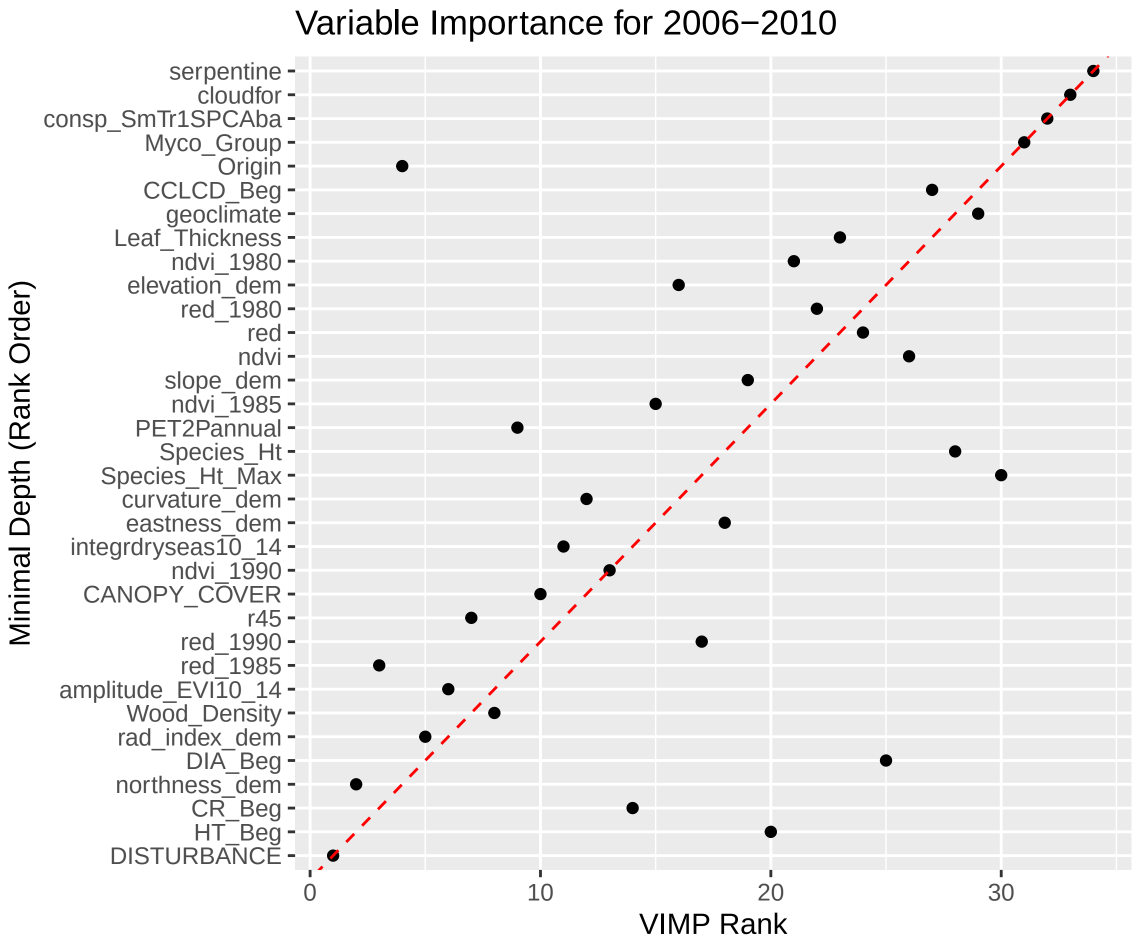

Figure 4: Ranked variable importance for 2006-2010 (t2). Ranked variable importance plot for permuted importance (x-axis) and minimal depth (y-axis) in the random forest model in t2, for surveys that began in 2001-2004, two years after Hurricane George, and ended in 2006-2009.

Fig 5 in S2 Supporting. Variable importance for the model interval ending in 2011-2014 (t3)

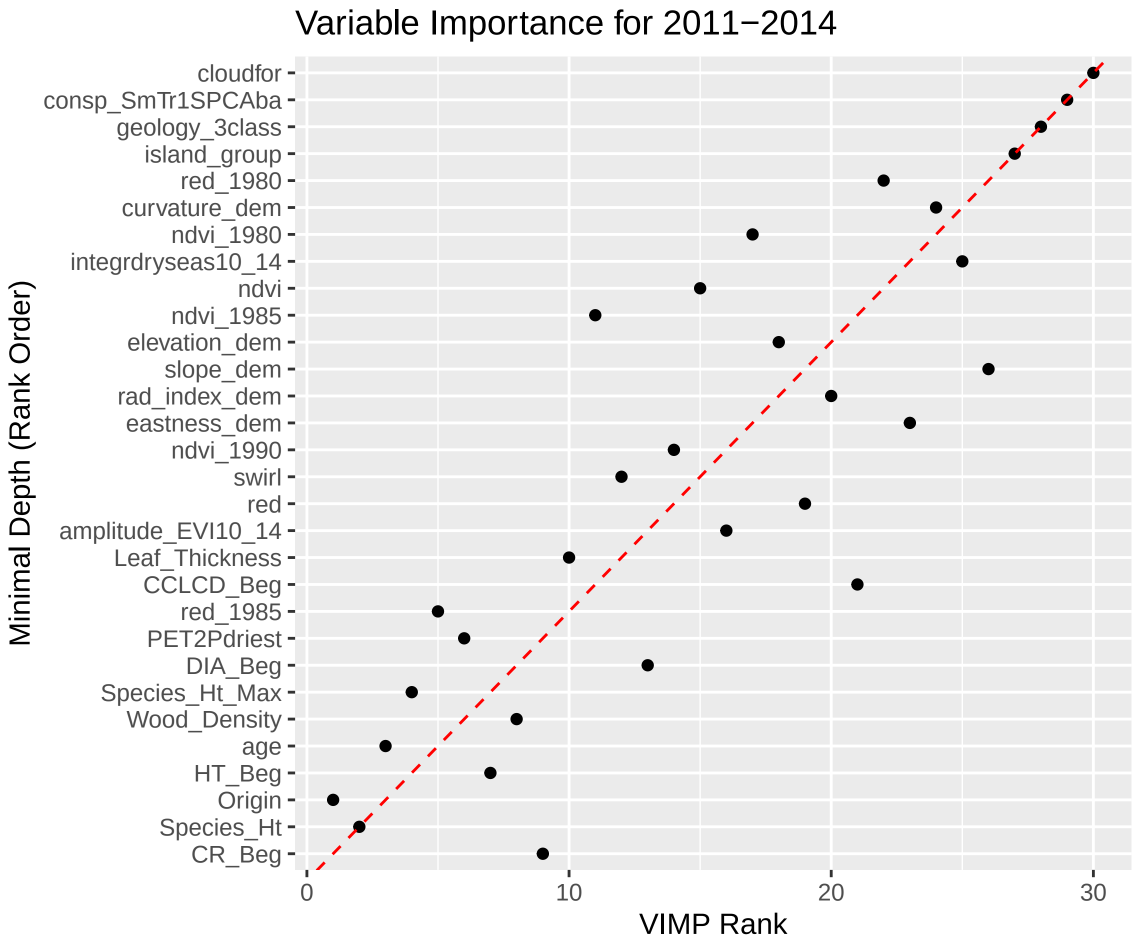

Figure 5: Ranked variable importance for 2011-2014 (t3). Ranked variable importance plot for permuted importance (x-axis) and minimal depth (y-axis) in the random forest model in t3, for surveys ending in 2011-2014.

Fig 6 in S2 Supporting. Variable importance for the model interval ending in 2016-2017 (t4a)

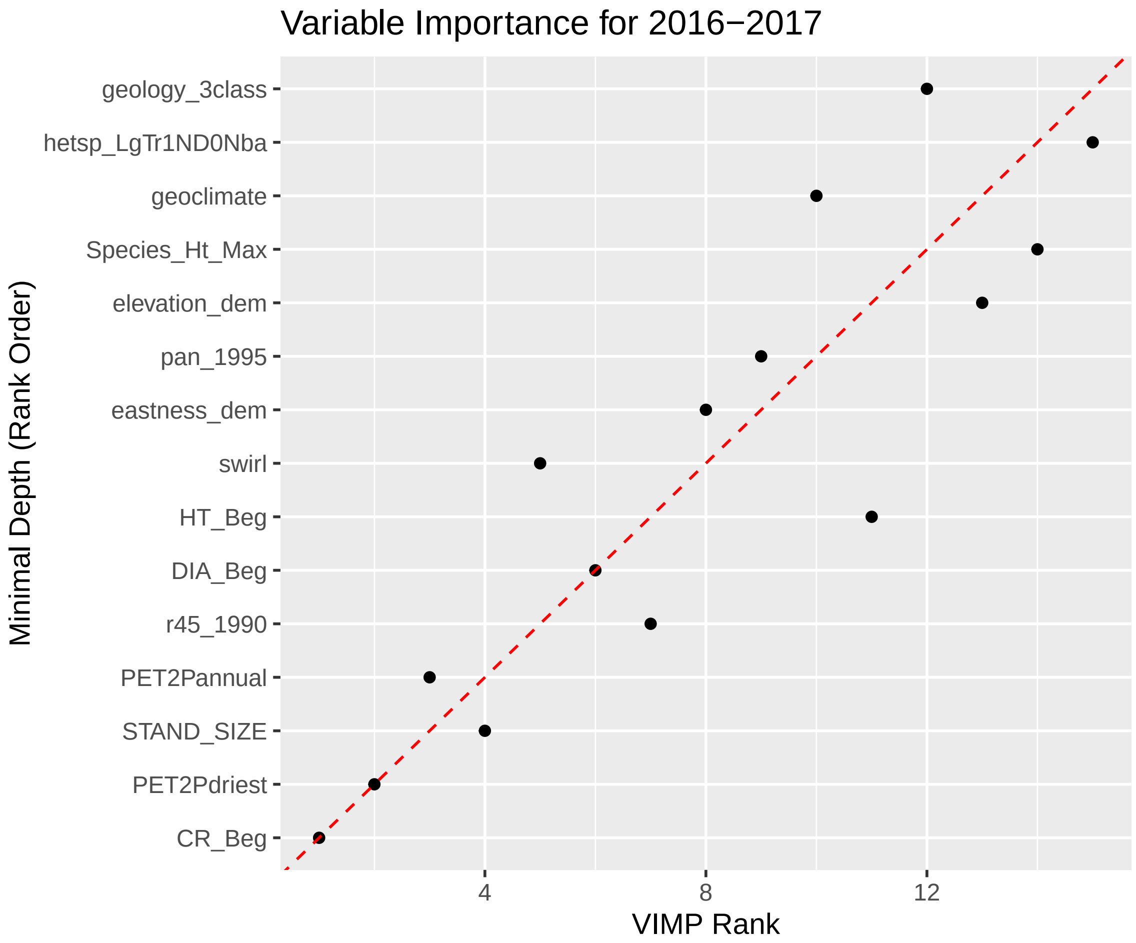

Figure 6: Ranked variable importance plot for interval spanning severe drought, ending in 2016-2017, but before Hurricanes Maria and Irma. Permuted importance (x-axis) and minimal depth (y-axis) in the random forest model in t4a.

Fig 7 in S2 Supporting. Variable importance for the model interval ending in 2017-2019 (t4b)

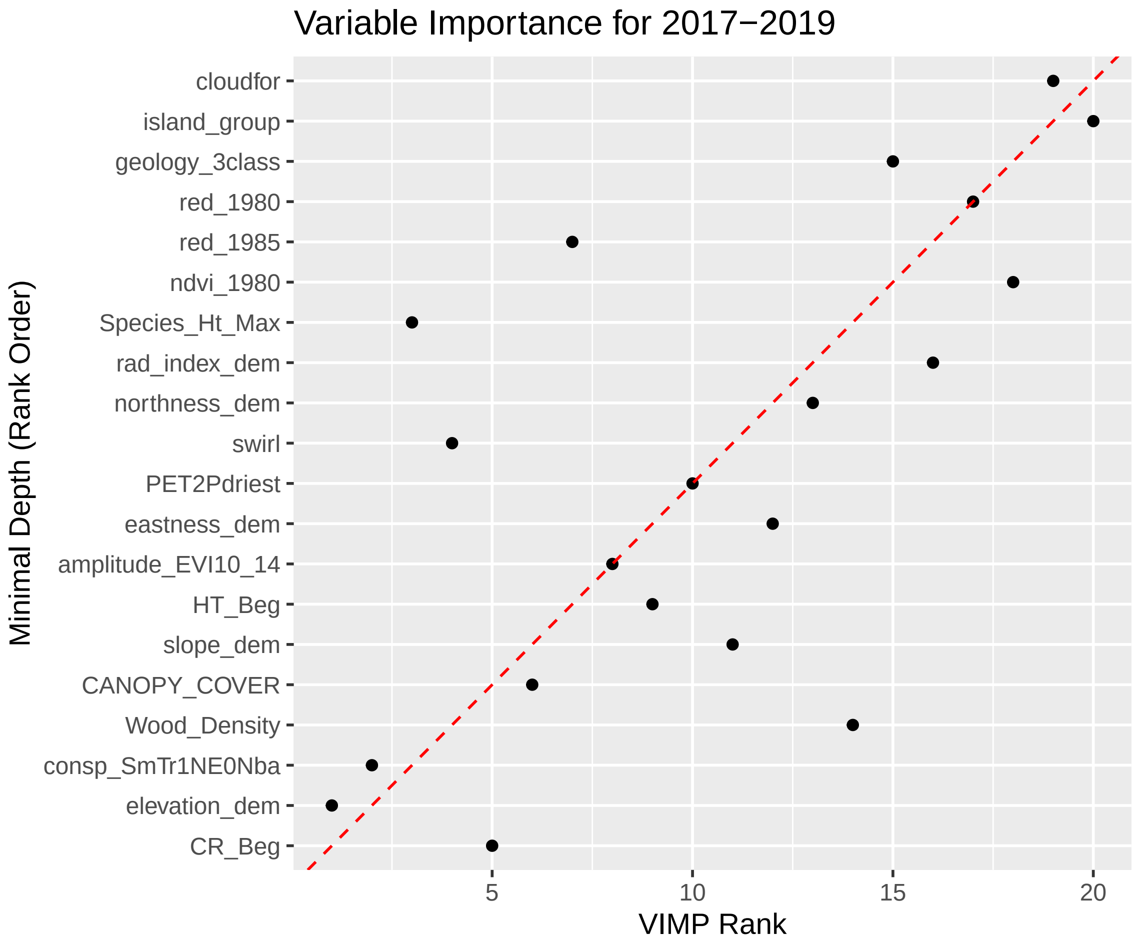

Figure 7: Ranked variable importance plot for the hurricane interval. Permuted variable importance plot (x-axis) and minimal depth (y-axis) for the random forest model for interval t4b, ending in 2017-2019, and spanning Hurricanes Maria and Irma.

Fig 8 in S2 Supporting. Wood density of introduced vs. native tree species

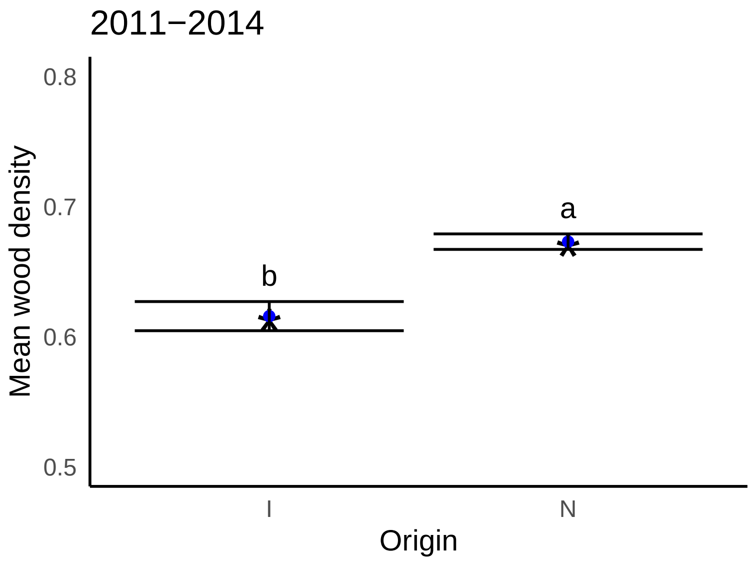

Figure 8: Mean wood density of small tree observations of introduced vs. native species in 2011-2014. On average, small trees of native species have denser wood.

Fig 9 in S2 Supporting. Survival probability vs. small conspecific NE0N trees

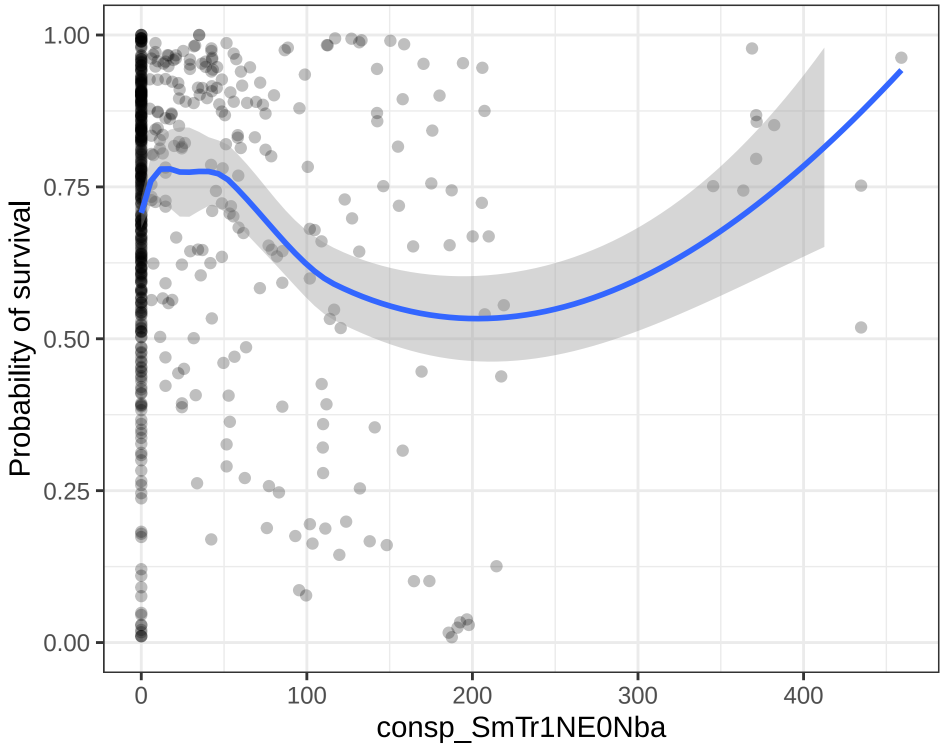

Figure 9: Marginal plot of predicted probability of survival NE0N species (Native Evergreen Non-N-Fixing) vs. small conspecifics, post-hurricanes model (t4b, 2017-2019). Survivors at hig NE0N basal areas are on humid karst hilltops.

Fig 10 in S2 Supporting. Survival by depth to water table and geoclimate

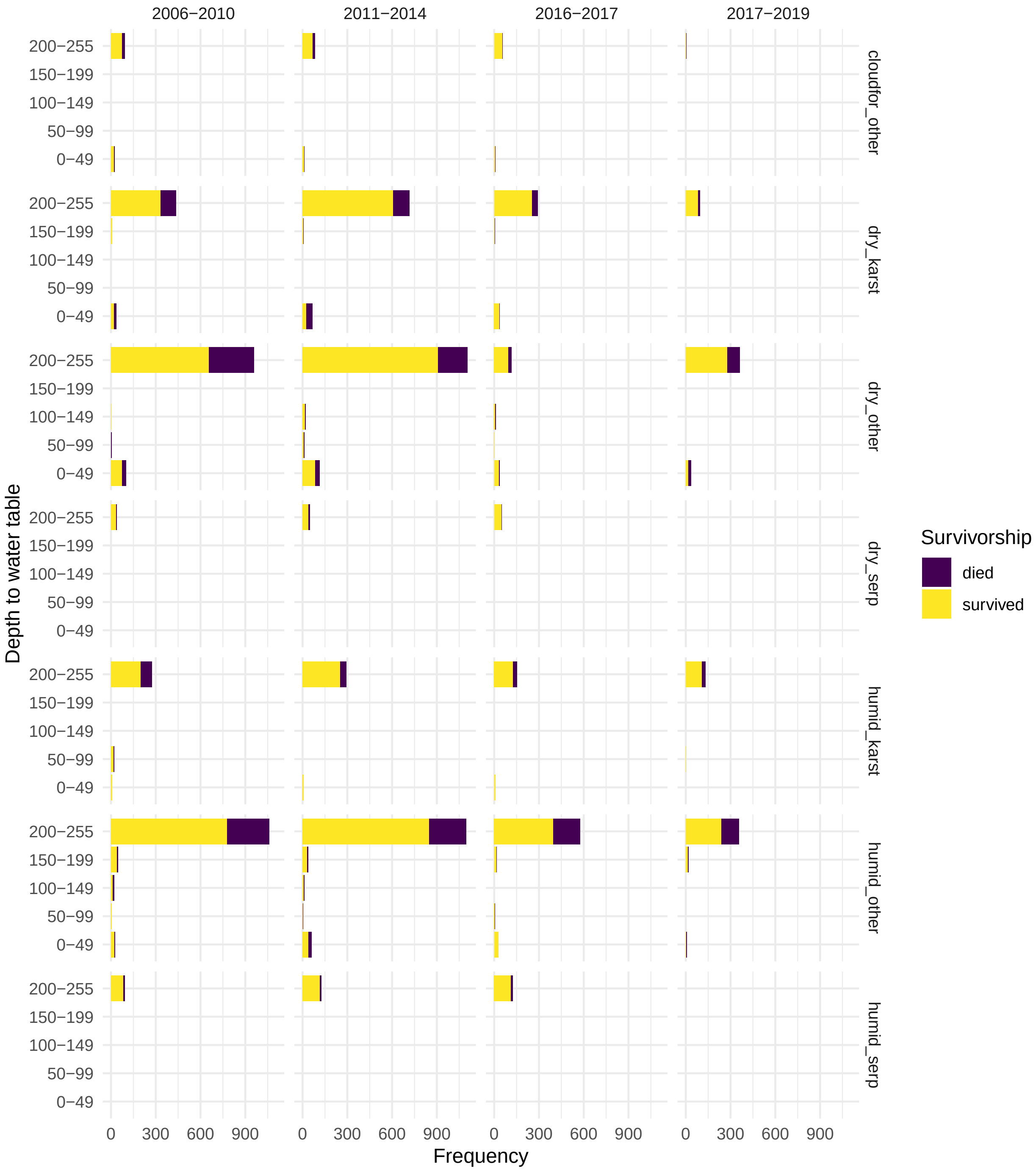

Figure 10: Counts of survived and died observations by interval, depth to water table and geoclimate.
