# Supplementary material for: Multiscale predictors of small tree survival across a heterogeneous tropical landscape
Source: PLoS One. 2023 Mar 15;18(3):e0280322. doi: 10.1371/journal.pone.0280322 (PMC10016699; doi:10.1371/journal.pone.0280322)
Supplement: S3 File — (PDF) [file pone.0280322.s003.pdf]

S3 Supporting Information for "Multiscale predictors of small tree mortality across a heterogeneous tropical landscape https://doi.org/10.1371/journal.pone.0280322"

Contents

Fig 1 in S3 Supporting. Top variables for t2, ending 2006-2009, by minimal depth rank 2
Fig 2 in S3 Supporting. Top variables for t2, ending 2006-2009, by permuted importance rank . . . 3
Fig 3 in S3 Supporting. Top variables for t3, ending 2011-2014, by minimal depth rank 4
Fig 4 in S3 Supporting. Top variables for t3, ending 2011-2014, by permuted importance rank . . . 5
Fig 5 in S3 Supporting. Top variables for t4a, ending 2016-2017, by minimal depth rank 6
Fig 6 in S3 Supporting. Top variables for t4a, ending 2016-2017, by permuted importance rank . . 7
Fig 7 in S3 Supporting. Top variables for t4b, ending 2017-2019, by minimal depth rank 8
Fig 8 in S3 Supporting. Top variables for t4b, ending 2017-2019, by permuted importance rank . . 9
Fig 9 in S3 Supporting. Top variables for the all-periods model, by minimal depth rank 10
Fig 10 in S3 Supporting. Top variables for the all-periods model, by permuted importance rank . . 11
Fig 11 in S3 Supporting. Marginal plots through time for additional topographic variables 12
Fig 12 in S3 Supporting. Marginal plots through time for the cloud forest geoclimate zone. 13

Fig 1 in S3 Supporting. Top variables for t2, ending 2006-2009, by minimal depth rank

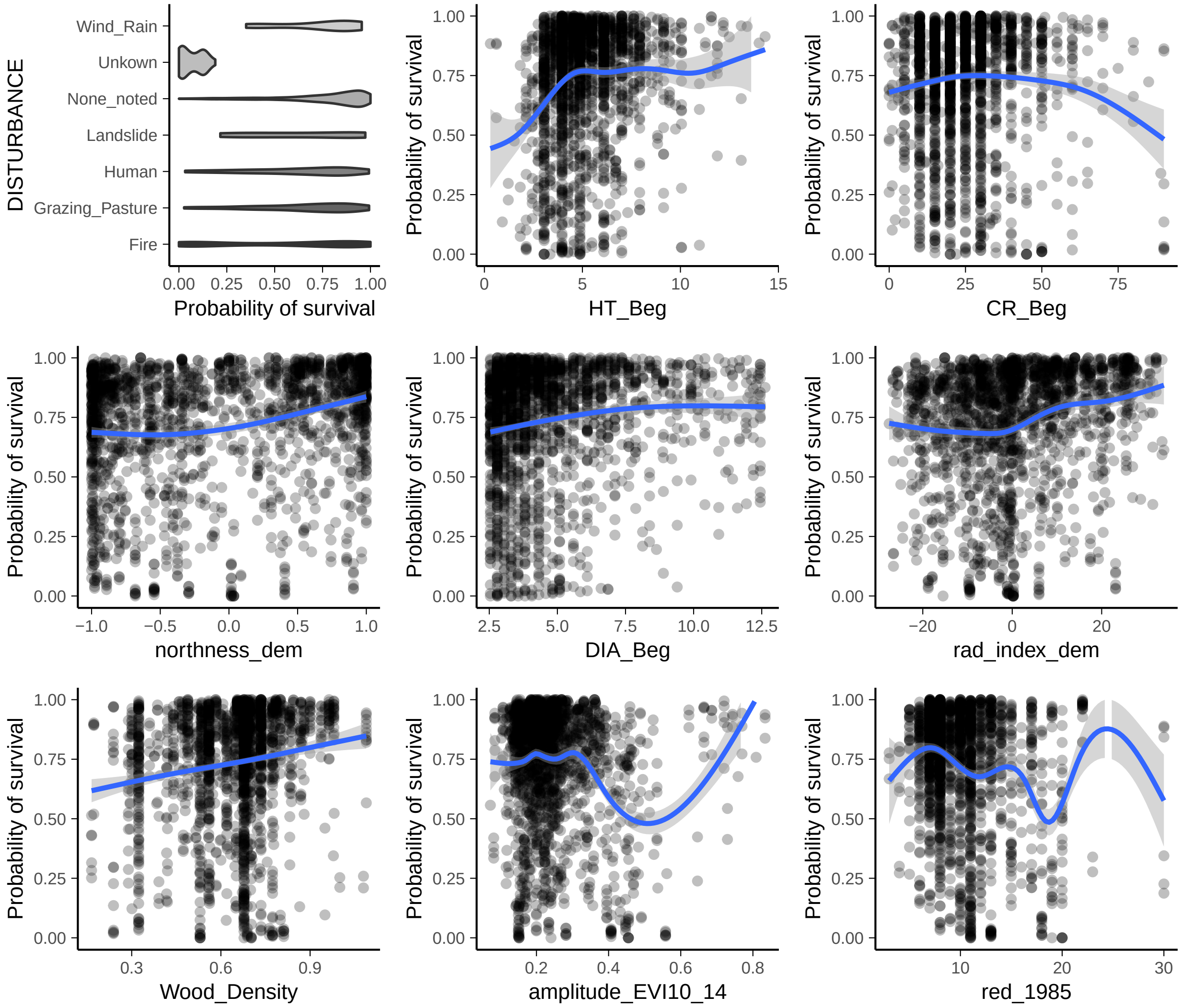

Figure 1: Marginal plots for the top nine variables in the t2 Random Forests model (ending in 2006-2009) as ranked by minimal depth.

Fig 2 in S3 Supporting. Top variables for t2, ending 2006-2009, by permuted importance rank

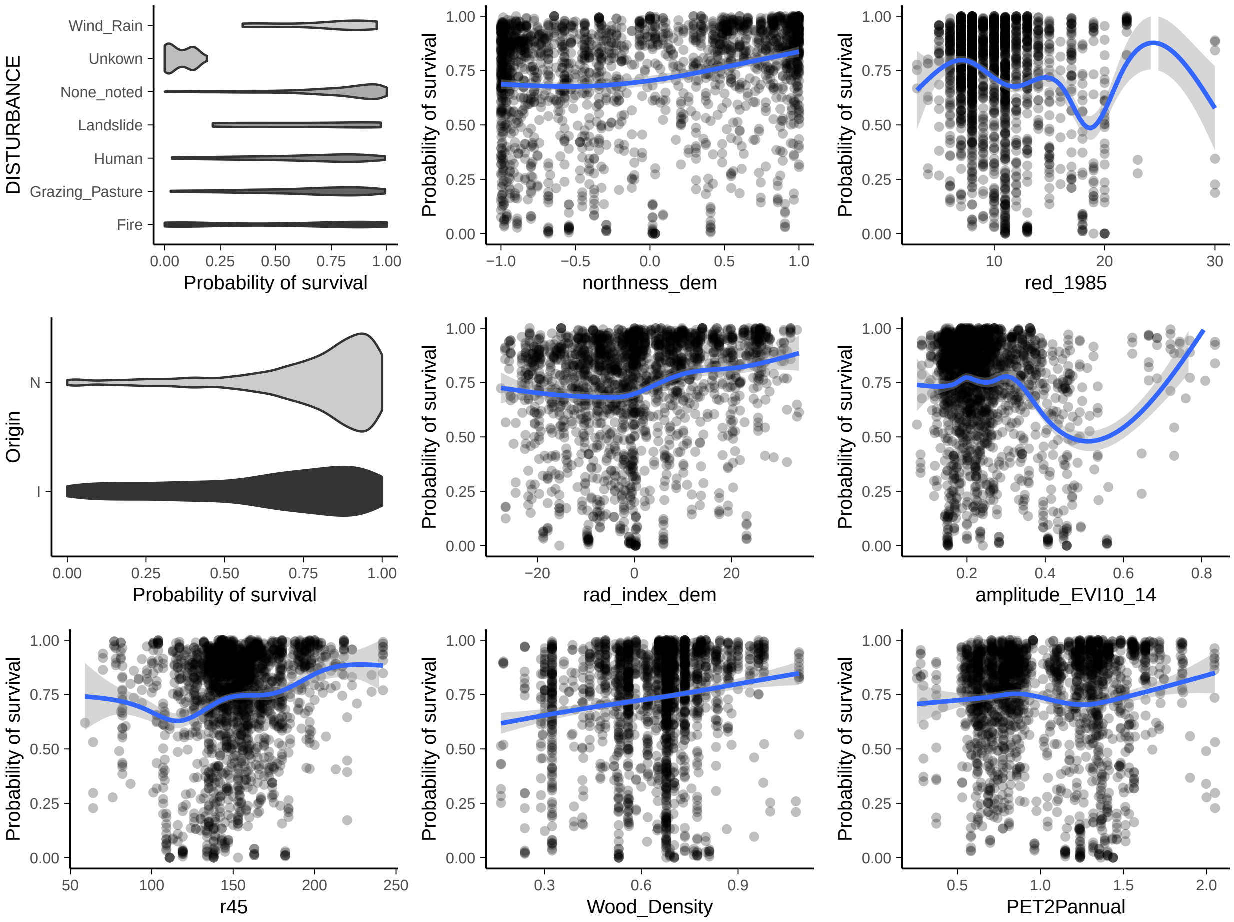

Figure 2: Marginal plots for the top nine variables in the t2 Random Forests model (ending in 2006-2009) as ranked by permuted importance.

Fig 3 in S3 Supporting. Top variables for t3, ending 2011-2014, by minimal depth rank

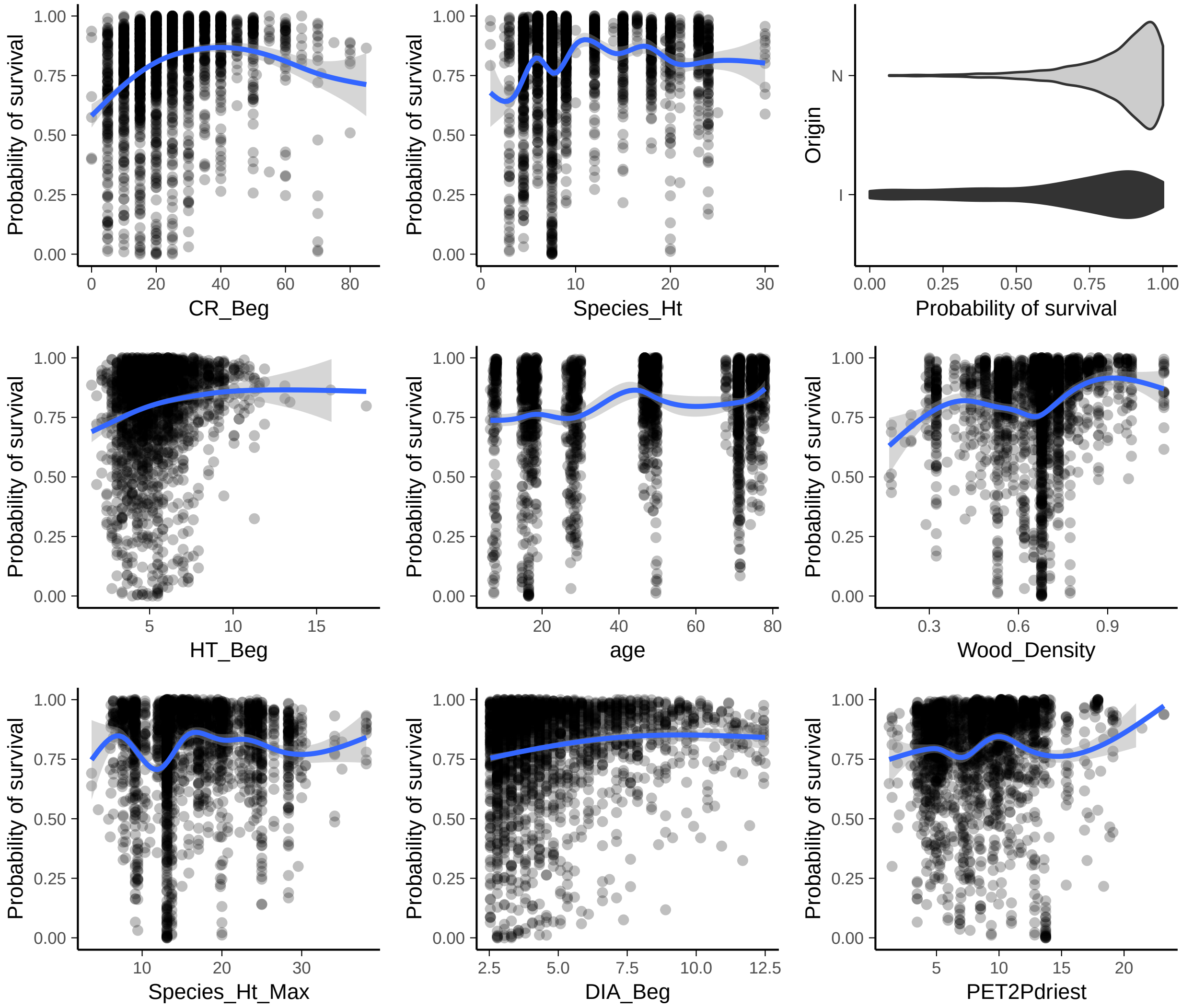

Figure 3: Marginal plots for the top nine variables in the t3 RF model (ending in 2011-2014) as ranked by minimal depth.

Fig 4 in S3 Supporting. Top variables for t3, ending 2011-2014, by permuted importance rank

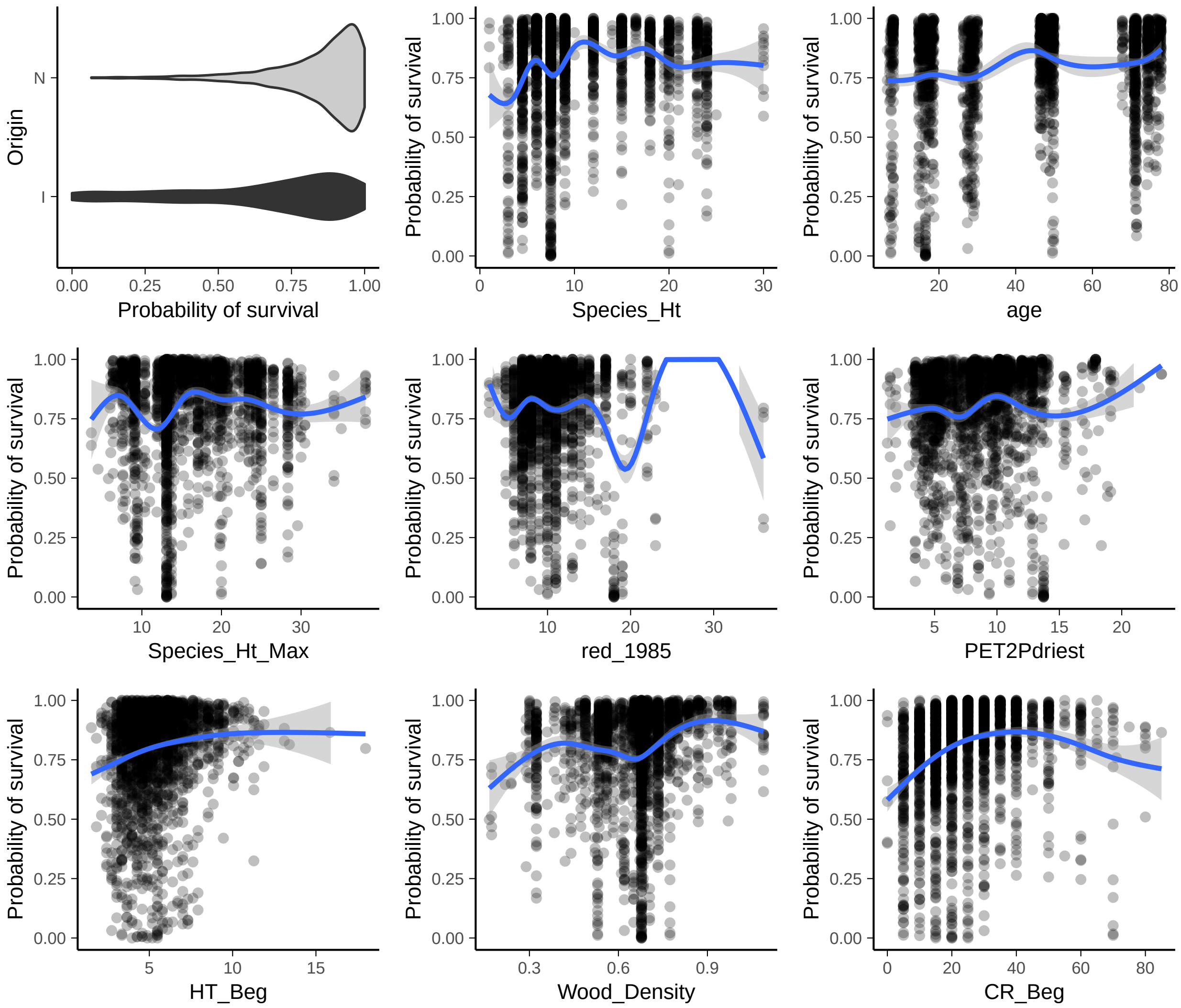

Figure 4: Marginal plots for the top nine variables in the t3 RF model (ending in 2011-2014) as ranked by permuted importance.

Fig 5 in S3 Supporting. Top variables for t4a, ending 2016-2017, by minimal depth rank

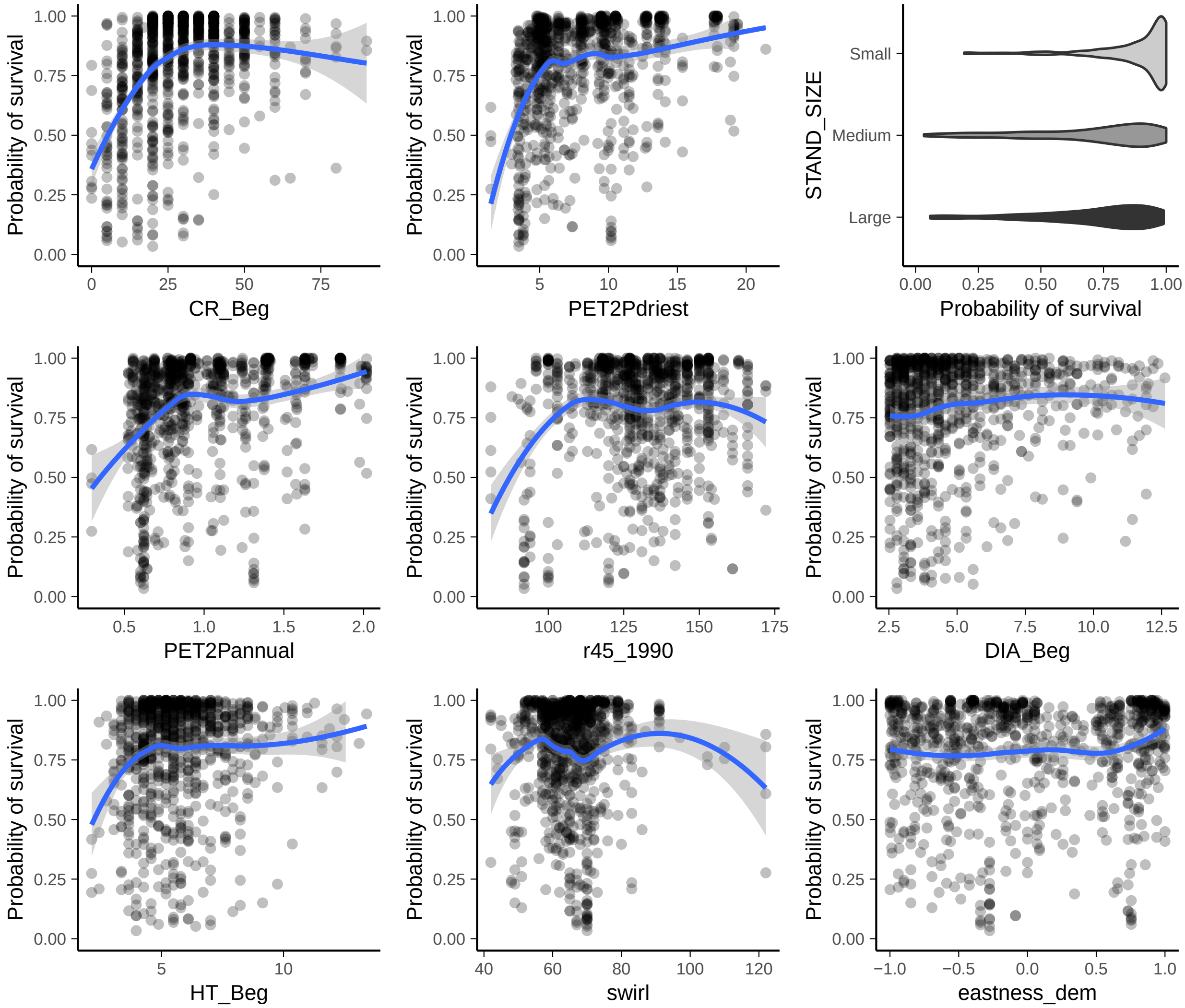

Figure 5: Marginal plots for the top nine variables in the t4a RF model (ending in 2016-2017) as ranked by minimal depth.

Fig 6 in S3 Supporting. Top variables for t4a, ending 2016-2017, by permuted importance rank

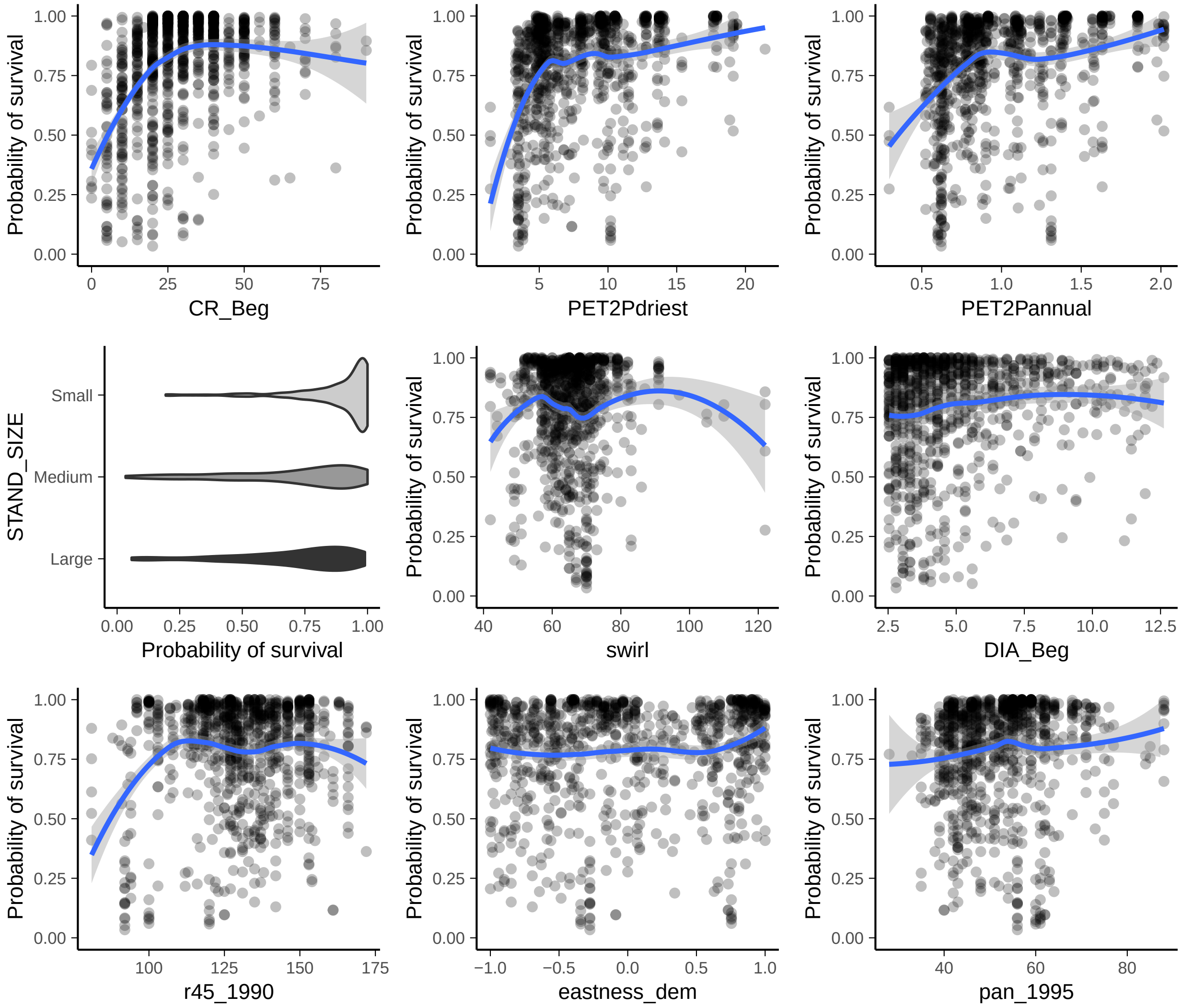

Figure 6: Marginal plots for the top nine variables in the t4a RF model (ending in 2016-2017) as ranked by permuted importance.

Fig 7 in S3 Supporting. Top variables for t4b, ending 2017-2019, by minimal depth rank

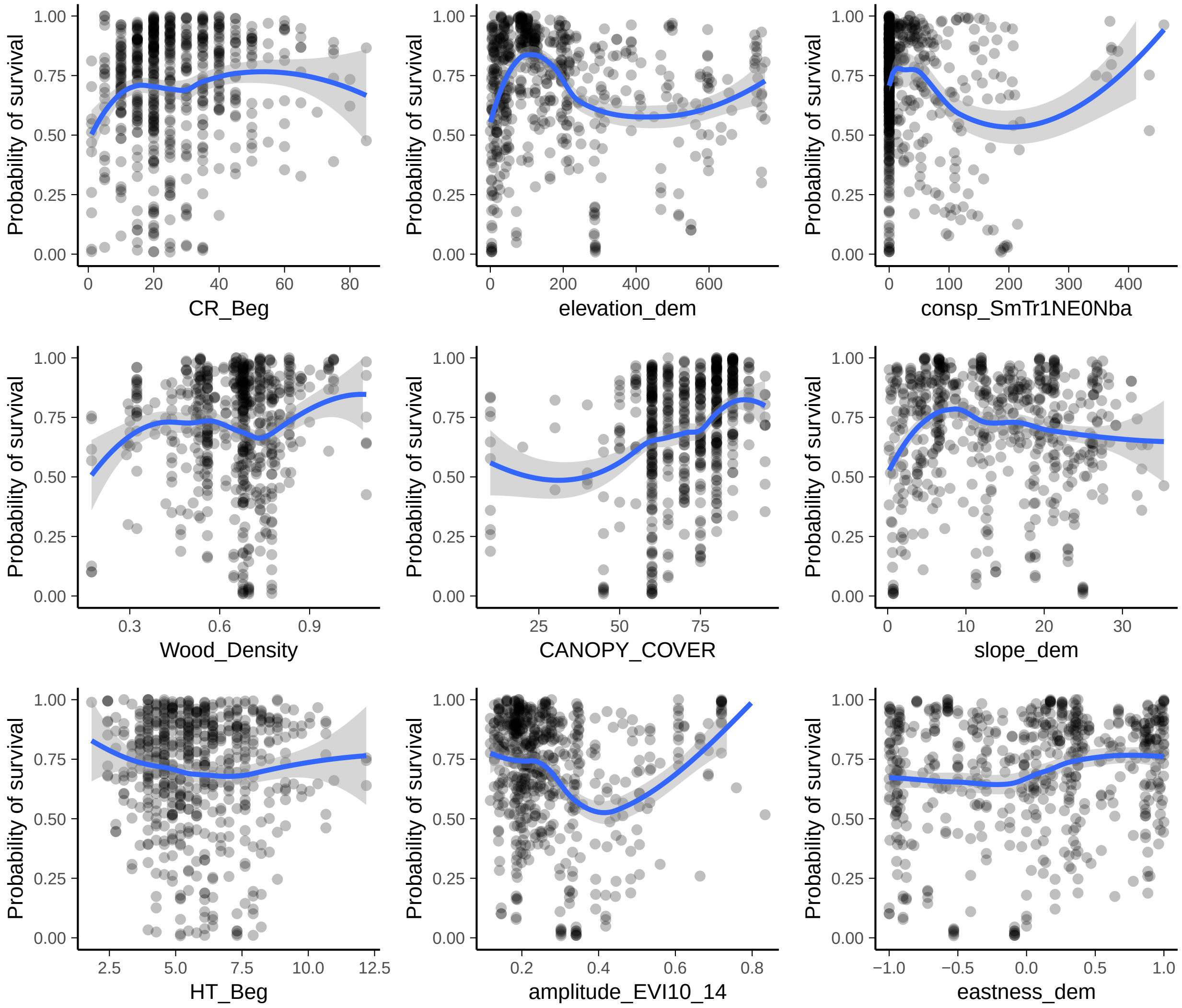

Figure 7: Marginal plots for the top nine variables in the t4b RF model (ending in 2017-2019) as ranked by minimal depth.

Fig 8 in S3 Supporting. Top variables for t4b, ending 2017-2019, by permuted importance rank

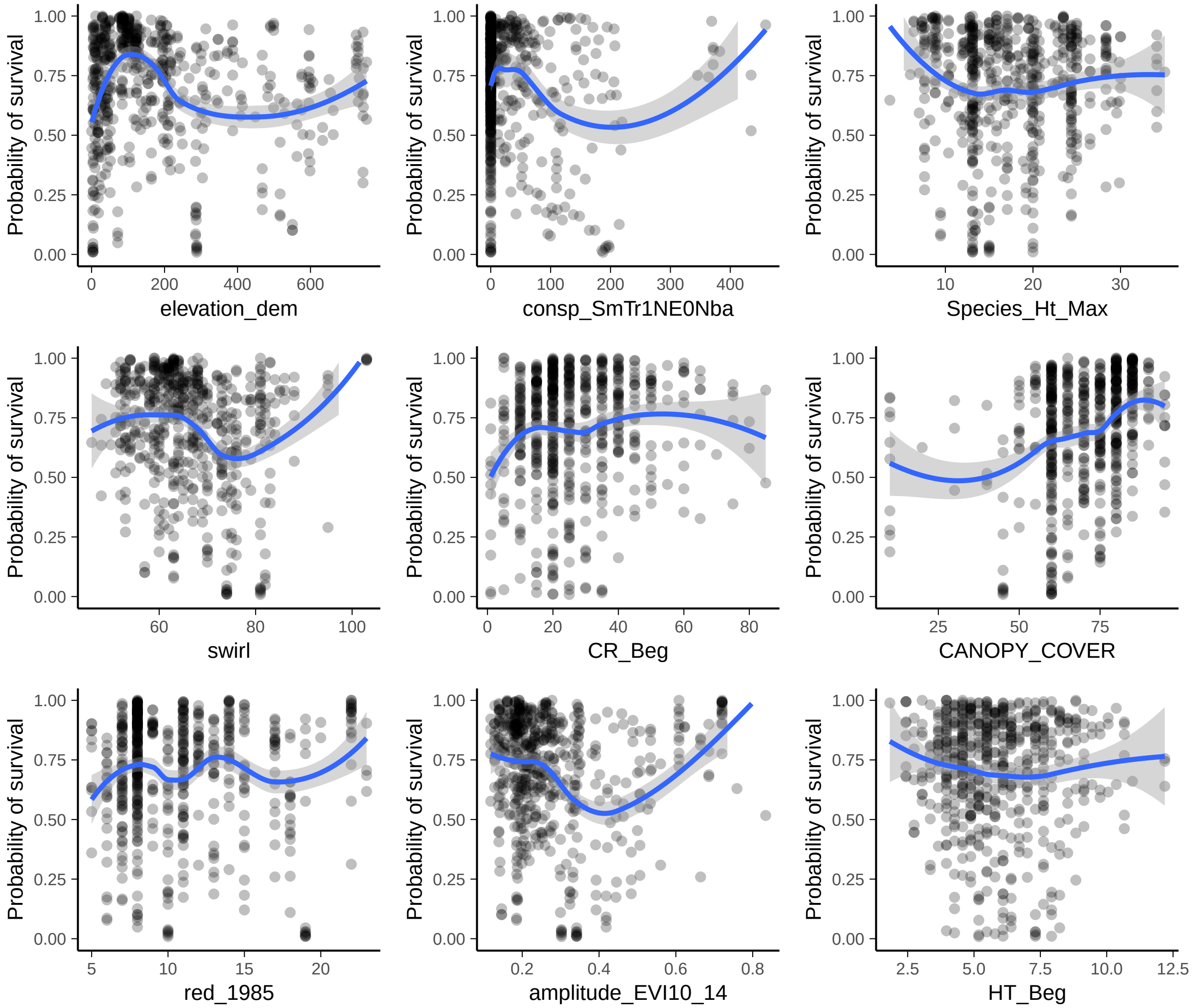

Figure 8: Marginal plots for the top nine variables in the t4b RF model (ending in 2017-2019) as ranked by permuted importance.

Fig 9 in S3 Supporting. Top variables for the all-periods model, by minimal depth rank

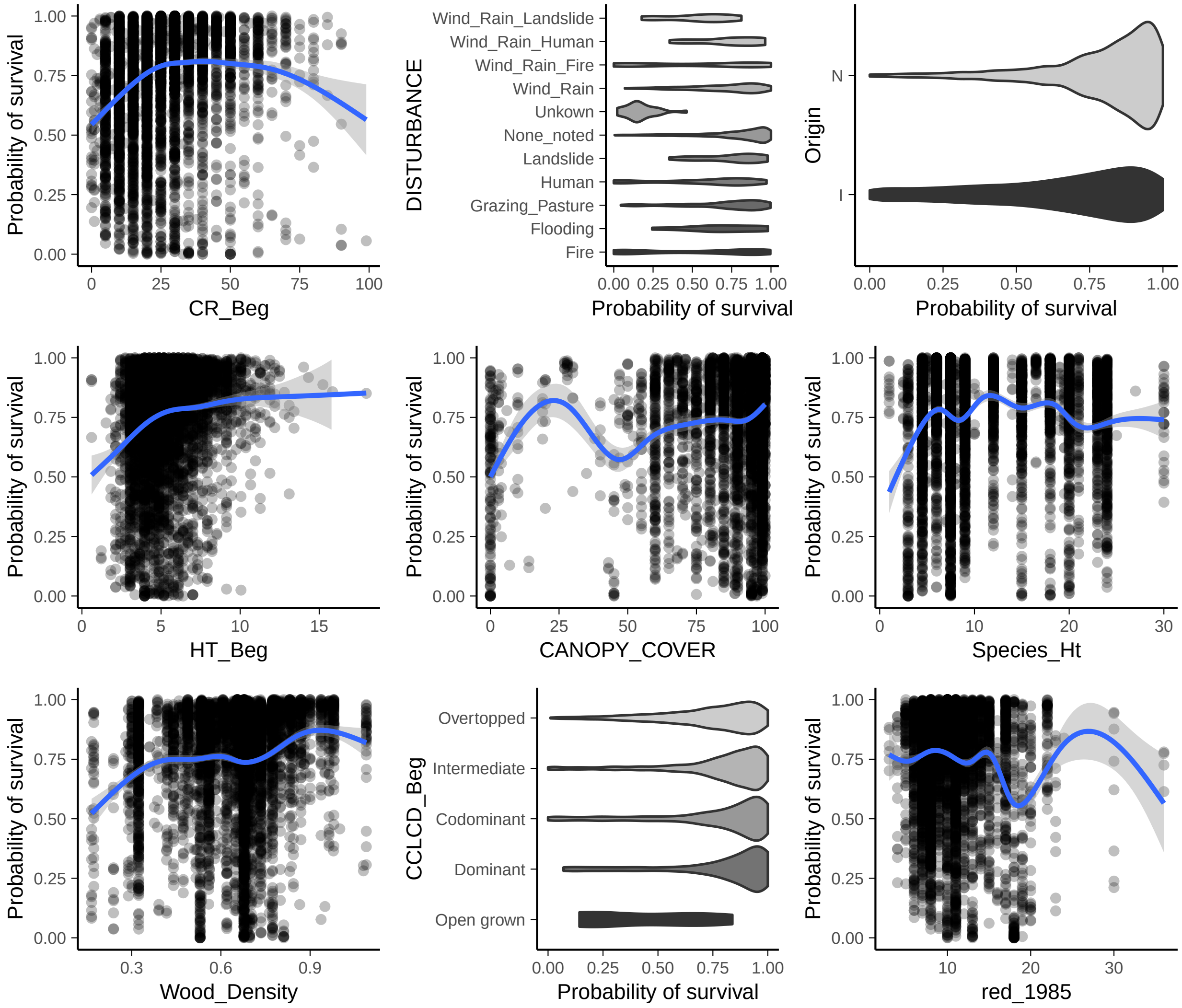

Figure 9: Marginal plots for the top nine variables in the all-periods model as ranked by minimal depth.

Fig 10 in S3 Supporting. Top variables for the all-periods model, by permuted importance rank

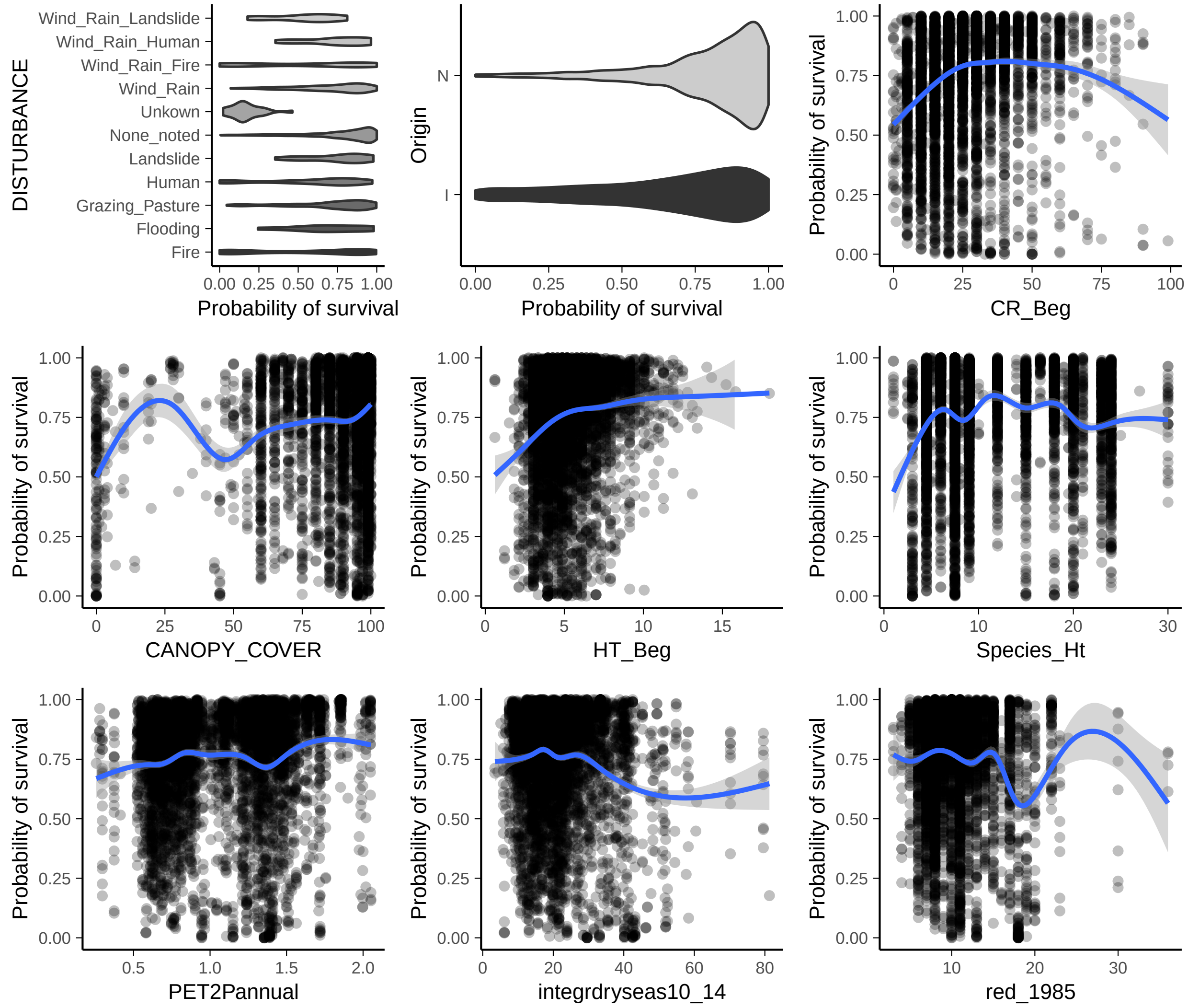

Figure 10: Marginal plots for the top nine variables in the all-periods RF model as ranked by permuted importance.

Fig 11 in S3 Supporting. Marginal plots through time for additional topographic variables

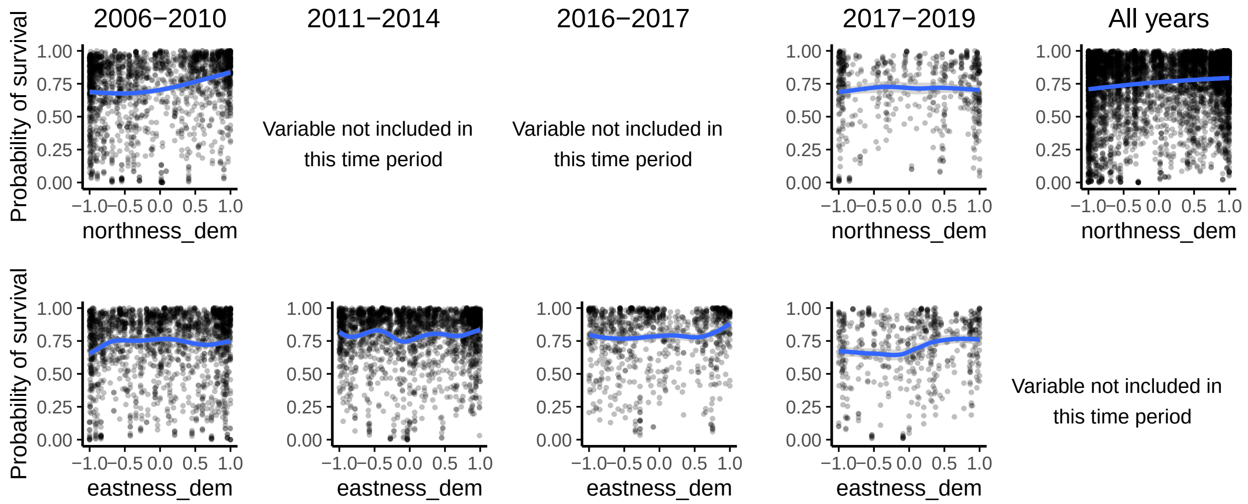

Figure 11: Marginal plots through time for northness and eastness, which were ranked among top predictors in 2006-2010.

Fig 12 in S3 Supporting. Marginal plots through time for the cloud forest geoclimate zone.

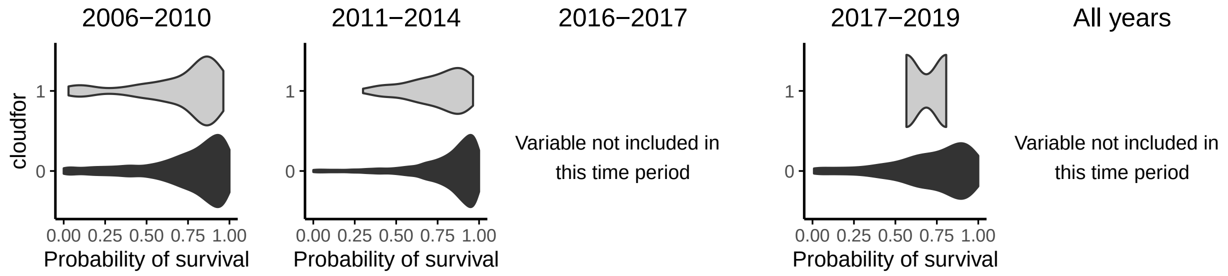

Figure 12: Marginal plots through time for the cloud forest geoclimate zone.
